# An expanded evaluation of protein function prediction methods shows an improvement in accuracy

**DOI:** 10.1186/s13059-016-1037-6

**Published:** 2016-09-07

**Authors:** Yuxiang Jiang, Tal Ronnen Oron, Wyatt T. Clark, Asma R. Bankapur, Daniel D’Andrea, Rosalba Lepore, Christopher S. Funk, Indika Kahanda, Karin M. Verspoor, Asa Ben-Hur, Da Chen Emily Koo, Duncan Penfold-Brown, Dennis Shasha, Noah Youngs, Richard Bonneau, Alexandra Lin, Sayed M. E. Sahraeian, Pier Luigi Martelli, Giuseppe Profiti, Rita Casadio, Renzhi Cao, Zhaolong Zhong, Jianlin Cheng, Adrian Altenhoff, Nives Skunca, Christophe Dessimoz, Tunca Dogan, Kai Hakala, Suwisa Kaewphan, Farrokh Mehryary, Tapio Salakoski, Filip Ginter, Hai Fang, Ben Smithers, Matt Oates, Julian Gough, Petri Törönen, Patrik Koskinen, Liisa Holm, Ching-Tai Chen, Wen-Lian Hsu, Kevin Bryson, Domenico Cozzetto, Federico Minneci, David T. Jones, Samuel Chapman, Dukka BKC, Ishita K. Khan, Daisuke Kihara, Dan Ofer, Nadav Rappoport, Amos Stern, Elena Cibrian-Uhalte, Paul Denny, Rebecca E. Foulger, Reija Hieta, Duncan Legge, Ruth C. Lovering, Michele Magrane, Anna N. Melidoni, Prudence Mutowo-Meullenet, Klemens Pichler, Aleksandra Shypitsyna, Biao Li, Pooya Zakeri, Sarah ElShal, Léon-Charles Tranchevent, Sayoni Das, Natalie L. Dawson, David Lee, Jonathan G. Lees, Ian Sillitoe, Prajwal Bhat, Tamás Nepusz, Alfonso E. Romero, Rajkumar Sasidharan, Haixuan Yang, Alberto Paccanaro, Jesse Gillis, Adriana E. Sedeño-Cortés, Paul Pavlidis, Shou Feng, Juan M. Cejuela, Tatyana Goldberg, Tobias Hamp, Lothar Richter, Asaf Salamov, Toni Gabaldon, Marina Marcet-Houben, Fran Supek, Qingtian Gong, Wei Ning, Yuanpeng Zhou, Weidong Tian, Marco Falda, Paolo Fontana, Enrico Lavezzo, Stefano Toppo, Carlo Ferrari, Manuel Giollo, Damiano Piovesan, Silvio C.E. Tosatto, Angela del Pozo, José M. Fernández, Paolo Maietta, Alfonso Valencia, Michael L. Tress, Alfredo Benso, Stefano Di Carlo, Gianfranco Politano, Alessandro Savino, Hafeez Ur Rehman, Matteo Re, Marco Mesiti, Giorgio Valentini, Joachim W. Bargsten, Aalt D. J. van Dijk, Branislava Gemovic, Sanja Glisic, Vladmir Perovic, Veljko Veljkovic, Nevena Veljkovic, Danillo C. Almeida-e-Silva, Ricardo Z. N. Vencio, Malvika Sharan, Jörg Vogel, Lakesh Kansakar, Shanshan Zhang, Slobodan Vucetic, Zheng Wang, Michael J. E. Sternberg, Mark N. Wass, Rachael P. Huntley, Maria J. Martin, Claire O’Donovan, Peter N. Robinson, Yves Moreau, Anna Tramontano, Patricia C. Babbitt, Steven E. Brenner, Michal Linial, Christine A. Orengo, Burkhard Rost, Casey S. Greene, Sean D. Mooney, Iddo Friedberg, Predrag Radivojac

**Affiliations:** 1 Department of Computer Science and Informatics, Indiana University, Bloomington, IN USA; 2 Buck Institute for Research on Aging, Novato, CA USA; 3 Department of Molecular Biophysics and Biochemistry, Yale University, New Haven, CT USA; 4 Department of Microbiology, Miami University, Oxford, OH USA; 5 University of Rome, La Sapienza, Rome, Italy; 6 Computational Bioscience Program, University of Colorado School of Medicine, Aurora, CO USA; 7 Department of Computer Science, Colorado State University, Fort Collins, CO USA; 8 Department of Computing and Information Systems, University of Melbourne, Parkville, Victoria, Australia; 9 Health and Biomedical Informatics Centre, University of Melbourne, Parkville, Victoria, Australia; 10 Department of Biology, New York University, New York, NY USA; 11 Social Media and Political Participation Lab, New York University, New York, NY USA; 12 CY Data Science, New York, NY USA; 13 Department of Computer Science, New York University, New York, NY USA; 14 Simons Center for Data Analysis, New York, NY USA; 15 Center for Genomics and Systems Biology, Department of Biology, New York University, New York, NY USA; 16 Department of Electrical Engineering and Computer Sciences, University of California Berkeley, Berkeley, CA USA; 17 Department of Plant and Microbial Biology, University of California Berkeley, Berkeley, CA USA; 18 Biocomputing Group, BiGeA, University of Bologna, Bologna, Italy; 19 Computer Science Department, University of Missouri, Columbia, MO USA; 20 ETH Zurich, Zurich, Switzerland; 21 Swiss Institute of Bioinformatics, Zurich, Switzerland; 22 Bioinformatics Group, Department of Computer Science, University College London, London, UK; 23 European Molecular Biology Laboratory, European Bioinformatics Institute, Cambridge, UK; 24 Department of Information Technology, University of Turku, Turku, Finland; 25 University of Turku Graduate School, University of Turku, Turku, Finland; 26 Turku Centre for Computer Science, Turku, Finland; 27 University of Bristol, Bristol, UK; 28 Institute of Biotechnology, University of Helsinki, Helsinki, Finland; 29 Institute of Information Science, Academia Sinica, Taipei, Taiwan; 30 Department of Computational Science and Engineering, North Carolina A&T State University, Greensboro, NC USA; 31 Department of Computer Science, Purdue University, West Lafayette, IN USA; 32 Department of Biological Chemistry, Institute of Life Sciences, The Hebrew University of Jerusalem, Jerusalem, Israel; 33 School of Computer Science and Engineering, The Hebrew University of Jerusalem, Jerusalem, Israel; 34 Centre for Integrative Systems Biology and Bioinformatics, Department of Life Sciences, Imperial College London, London, UK; 35 Centre for Cardiovascular Genetics, Institute of Cardiovascular Science, University College London, London, UK; 36 Department of Electrical Engineering, STADIUS Center for Dynamical Systems, Signal Processing and Data Analytics, KU Leuven, Leuven, Belgium; 37 iMinds Department Medical Information Technologies, Leuven, Belgium; 38 Inserm UMR-S1052, CNRS UMR5286, Cancer Research Centre of Lyon, Lyon, France; 39 Université de Lyon 1, Villeurbanne, France; 40 Centre Léon Bérard, Lyon, France; 41 Institute of Structural and Molecular Biology, University College London, London, UK; 42 Cerenode Inc., Boston, MA USA; 43 Molde University College, Molde, Norway; 44 Department of Computer Science, Centre for Systems and Synthetic Biology, Royal Holloway University of London, Egham, UK; 45 Department of Molecular, Cell and Developmental Biology, University of California at Los Angeles, Los Angeles, CA USA; 46 School of Mathematics, Statistics and Applied Mathematics, National University of Ireland, Galway, Ireland; 47 Stanley Institute for Cognitive Genomics Cold Spring Harbor Laboratory, New York, NY USA; 48 Graduate Program in Bioinformatics, University of British Columbia, Vancouver, Canada; 49 Department of Psychiatry and Michael Smith Laboratories, University of British Columbia, Vancouver, Canada; 50 Department for Bioinformatics and Computational Biology-I12, Technische Universität München, Garching, Germany; 51 DOE Joint Genome Institute, Walnut Creek, CA USA; 52 Bioinformatics and Genomics, Centre for Genomic Regulation, Barcelona, Spain; 53 Universitat Pompeu Fabra, Barcelona, Spain; 54 Institució Catalana de Recerca i Estudis Avançats, Barcelona, Spain; 55 Division of Electronics, Rudjer Boskovic Institute, Zagreb, Croatia; 56 EMBL/CRG Systems Biology Research Unit, Centre for Genomic Regulation, Barcelona, Spain; 57 State Key Laboratory of Genetic Engineering, Collaborative Innovation Center of Genetics and Development, Department of Biostatistics and Computational Biology, School of Life Science, Fudan University, Shanghai, China; 58 Children’s Hospital of Fudan University, Shanghai, China; 59 Department of Molecular Medicine, University of Padua, Padua, Italy; 60 Research and Innovation Center, Edmund Mach Foundation, San Michele all’Adige, Italy; 61 Department of Information Engineering, University of Padua, Padova, Italy; 62 Instituto De Genetica Medica y Molecular, Hospital Universitario de La Paz, Madrid, Spain; 63 Spanish National Bioinformatics Institute, Spanish National Cancer Research Institute, Madrid, Spain; 64 Structural and Computational Biology Programme, Spanish National Cancer Research Institute, Madrid, Spain; 65 Control and Computer Engineering Department, Politecnico di Torino, Torino, Italy; 66 National University of Computer & Emerging Sciences, Islamabad, Pakistan; 67 Anacleto Lab, Dipartimento di informatica, Università degli Studi di Milano, Milan, Italy; 68 Applied Bioinformatics, Bioscience, Wageningen University and Research Centre, Wageningen, Netherlands; 69 Biometris, Wageningen University, Wageningen, Netherlands; 70 Center for Multidisciplinary Research, Institute of Nuclear Sciences Vinca, University of Belgrade, Belgrade, Serbia; 71 Department of Computing and Mathematics FFCLRP-USP, University of Sao Paulo, Ribeirao Preto, Brazil; 72 Institute for Molecular Infection Biology, University of Würzburg, Würzburg, Germany; 73 Department of Computer and Information Sciences, Temple University, Philadelphia, PA USA; 74 University of Southern Mississippi, Hattiesburg, MS USA; 75 School of Biosciences, University of Kent, Canterbury, Kent, UK; 76 Institut für Medizinische Genetik und Humangenetik, Charité - Universitätsmedizin Berlin, Berlin, Germany; 77 Department of Electrical Engineering ESAT-SCD and IBBT-KU Leuven Future Health Department, Katholieke Universiteit Leuven, Leuven, Belgium; 78 California Institute for Quantitative Biosciences, University of California San Francisco, San Francisco, CA USA; 79 Department of Chemical Biology, The Hebrew University of Jerusalem, Jerusalem, Israel; 80 Department of Systems Pharmacology and Translational Therapeutics, University of Pennsylvania, Philadelphia, PA USA; 81 Department of Biomedical Informatics and Medical Education, University of Washington, Seattle, WA USA; 82 Department of Computer Science, Miami University, Oxford, OH USA; 83 Department of Veterinary Microbiology and Preventive Medicine, Iowa State University, Ames, IA USA; 84 Department of Biomedical Sciences, University of Padua, Padova, Italy; 85 Department of Biological Sciences, Purdue University, West Lafayette, IN USA; 86 Department of Biological and Environmental Sciences, Universitity of Helsinki, Helsinki, Finland; 87 University of Lausanne, Lausanne, Switzerland; 88 Swiss Institute of Bioinformatics, Lausanne, Switzerland

**Keywords:** Protein function prediction, Disease gene prioritization

## Abstract

**Background:**

A major bottleneck in our understanding of the molecular underpinnings of life is the assignment of function to proteins. While molecular experiments provide the most reliable annotation of proteins, their relatively low throughput and restricted purview have led to an increasing role for computational function prediction. However, assessing methods for protein function prediction and tracking progress in the field remain challenging.

**Results:**

We conducted the second critical assessment of functional annotation (CAFA), a timed challenge to assess computational methods that automatically assign protein function. We evaluated 126 methods from 56 research groups for their ability to predict biological functions using Gene Ontology and gene-disease associations using Human Phenotype Ontology on a set of 3681 proteins from 18 species. CAFA2 featured expanded analysis compared with CAFA1, with regards to data set size, variety, and assessment metrics. To review progress in the field, the analysis compared the best methods from CAFA1 to those of CAFA2.

**Conclusions:**

The top-performing methods in CAFA2 outperformed those from CAFA1. This increased accuracy can be attributed to a combination of the growing number of experimental annotations and improved methods for function prediction. The assessment also revealed that the definition of top-performing algorithms is ontology specific, that different performance metrics can be used to probe the nature of accurate predictions, and the relative diversity of predictions in the biological process and human phenotype ontologies. While there was methodological improvement between CAFA1 and CAFA2, the interpretation of results and usefulness of individual methods remain context-dependent.

**Electronic supplementary material:**

The online version of this article (doi:10.1186/s13059-016-1037-6) contains supplementary material, which is available to authorized users.

## Background

Accurate computer-generated functional annotations of biological macromolecules allow biologists to rapidly generate testable hypotheses about the roles that newly identified proteins play in processes or pathways. They also allow them to reason about new species based on the observed functional repertoire associated with their genes. However, protein function prediction is an open research problem and it is not yet clear which tools are best for predicting function. At the same time, critically evaluating these tools and understanding the landscape of the function prediction field is a challenging task that extends beyond the capabilities of a single lab.

Assessments and challenges have a successful history of driving the development of new methods in the life sciences by independently assessing performance and providing discussion forums for the researchers [1]. In 2010–2011, we organized the first critical assessment of functional annotation (CAFA) challenge to evaluate methods for the automated annotation of protein function and to assess the progress in method development in the first decade of the 2000s [2]. The challenge used a time-delayed evaluation of predictions for a large set of target proteins without any experimental functional annotation. A subset of these target proteins accumulated experimental annotations after the predictions were submitted and was used to estimate the performance accuracy. The estimated performance was subsequently used to draw conclusions about the status of the field.

The first CAFA (CAFA1) showed that advanced methods for the prediction of Gene Ontology (GO) terms [3] significantly outperformed a straightforward application of function transfer by local sequence similarity. In addition to validating investment in the development of new methods, CAFA1 also showed that using machine learning to integrate multiple sequence hits and multiple data types tends to perform well. However, CAFA1 also identified challenges for experimentalists, biocurators, and computational biologists. These challenges include the choice of experimental techniques and proteins in functional studies and curation, the structure and status of biomedical ontologies, the lack of comprehensive systems data that are necessary for accurate prediction of complex biological concepts, as well as limitations of evaluation metrics [2, 4–7]. Overall, by establishing the state-of-the-art in the field and identifying challenges, CAFA1 set the stage for quantifying progress in the field of protein function prediction over time.

In this study, we report on the major outcomes of the second CAFA experiment, CAFA2, that was organized and conducted in 2013–2014, exactly 3 years after the original experiment. We were motivated to evaluate the progress in method development for function prediction as well as to expand the experiment to new ontologies. The CAFA2 experiment also greatly expanded the performance analysis to new types of evaluation and included new performance metrics. By surveying the state of the field, we aim to help all direct and indirect users of computational function prediction software develop intuition for the quality, robustness, and reliability of these predictions.

## Methods

### Experiment overview

**Fig. 1:**
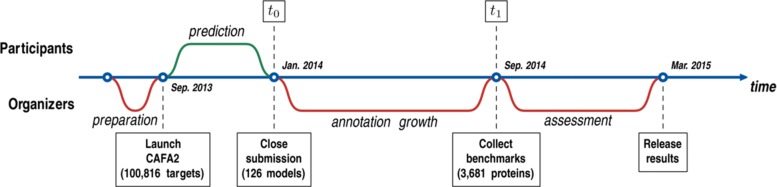
Time line for the CAFA2 experiment

The time line for the second CAFA experiment followed that of the first experiment and is illustrated in Fig. 1. Briefly, CAFA2 was announced in July 2013 and officially started in September 2013, when 100,816 *target sequences* from 27 species were made available to the community. Teams were required to submit prediction scores within the (0,1] range for each protein–term pair they chose to predict on. The submission deadline for depositing these predictions was set for January 2014 (time point *t*_0_). We then waited until September 2014 (time point *t*_1_) for new experimental annotations to accumulate on the target proteins and assessed the performance of the prediction methods. We will refer to the set of all experimentally annotated proteins available at *t*_0_ as the *training set* and to a subset of target proteins that accumulated experimental annotations during (*t*_0_,*t*_1_] and used for evaluation as the *benchmark set*. It is important to note that the benchmark proteins and the resulting analysis vary based on the selection of time point *t*_1_. For example, a preliminary analysis of the CAFA2 experiment was provided during the Automated Function Prediction Special Interest Group (AFP-SIG) meeting at the Intelligent Systems for Molecular Biology (ISMB) conference in July 2014.

The participating methods were evaluated according to their ability to predict terms in GO [3] and Human Phenotype Ontology (HPO) [8]. In contrast with CAFA1, where the evaluation was carried out only for the Molecular Function Ontology (MFO) and Biological Process Ontology (BPO), in CAFA2 we also assessed the performance for the prediction of Cellular Component Ontology (CCO) terms in GO. The set of human proteins was further used to evaluate methods according to their ability to associate these proteins with disease terms from HPO, which included all sub-classes of the term HP:0000118, “Phenotypic abnormality”.

In total, 56 groups submitting 126 methods participated in CAFA2. From those, 125 methods made valid predictions on a sufficient number of sequences. Further, 121 methods submitted predictions for at least one of the GO benchmarks, while 30 methods participated in the disease gene prediction tasks using HPO.

### Evaluation

The CAFA2 experiment expanded the assessment of computational function prediction compared with CAFA1. This includes the increased number of targets, benchmarks, ontologies, and method comparison metrics.

We distinguish between two major types of method evaluation. The first, *protein-centric evaluation*, assesses performance accuracy of methods that predict all ontological terms associated with a given protein sequence. The second type, *term-centric evaluation*, assesses performance accuracy of methods that predict if a single ontology term of interest is associated with a given protein sequence [2]. The protein-centric evaluation can be viewed as a multi-label or structured-output learning problem of predicting a set of terms or a directed acyclic graph (a subgraph of the ontology) for a given protein. Because the ontologies contain many terms, the output space in this setting is extremely large and the evaluation metrics must incorporate similarity functions between groups of mutually interdependent terms (directed acyclic graphs). In contrast, the term-centric evaluation is an example of binary classification, where a given ontology term is assigned (or not) to an input protein sequence. These methods are particularly common in disease gene prioritization [9]. Put otherwise, a protein-centric evaluation considers a ranking of ontology terms for a given protein, whereas the term-centric evaluation considers a ranking of protein sequences for a given ontology term.

Both types of evaluation have merits in assessing performance. This is partly due to the statistical dependency between ontology terms, the statistical dependency among protein sequences, and also the incomplete and biased nature of the experimental annotation of protein function [6]. In CAFA2, we provide both types of evaluation, but we emphasize the protein-centric scenario for easier comparisons with CAFA1. We also draw important conclusions regarding method assessment in these two scenarios.

#### No-knowledge and limited-knowledge benchmark sets

In CAFA1, a protein was eligible to be in the benchmark set if it had not had any experimentally verified annotations in any of the GO ontologies at time *t*_0_ but accumulated at least one functional term with an experimental evidence code between *t*_0_ and *t*_1_; we refer to such benchmark proteins as *no-knowledge* benchmarks. In CAFA2 we introduced proteins with *limited knowledge*, which are those that had been experimentally annotated in one or two GO ontologies (but not in all three) at time *t*_0_. For example, for the performance evaluation in MFO, a protein without any annotation in MFO prior to the submission deadline was allowed to have experimental annotations in BPO and CCO.

During the growth phase, the no-knowledge targets that have acquired experimental annotations in one or more ontologies became benchmarks in those ontologies. The limited-knowledge targets that have acquired additional annotations became benchmarks only for those ontologies for which there were no prior experimental annotations. The reason for using limited-knowledge targets was to identify whether the correlations between experimental annotations across ontologies can be exploited to improve function prediction.

The selection of benchmark proteins for evaluating HPO-term predictors was separated from the GO analyses. We created only a no-knowledge benchmark set in the HPO category.

#### Partial and full evaluation modes

Many function prediction methods apply only to certain types of proteins, such as proteins for which 3D structure data are available, proteins from certain taxa, or specific subcellular localizations. To accommodate these methods, CAFA2 provided predictors with an option of choosing a subset of the targets to predict on as long as they computationally annotated at least 5,000 targets, of which at least ten accumulated experimental terms. We refer to the assessment mode in which the predictions were evaluated only on those benchmarks for which a model made at least one prediction at any threshold as *partial evaluation mode*. In contrast, the *full evaluation mode* corresponds to the same type of assessment performed in CAFA1 where all benchmark proteins were used for the evaluation and methods were penalized for not making predictions.

In most cases, for each benchmark category, we have two types of benchmarks, no-knowledge and limited-knowledge, and two modes of evaluation, full mode and partial mode. Exceptions are all HPO categories that only have no-knowledge benchmarks. The full mode is appropriate for comparisons of general-purpose methods designed to make predictions on any protein, while the partial mode gives an idea of how well each method performs on a self-selected subset of targets.

#### Evaluation metrics

Precision–recall curves and remaining uncertainty–misinformation curves were used as the two chief metrics in the protein-centric mode [10]. We also provide a single measure for evaluation of both types of curves as a real-valued scalar to compare methods; however, we note that any choice of a single point on those curves may not match the intended application objectives for a given algorithm. Thus, a careful understanding of the evaluation metrics used in CAFA is necessary to properly interpret the results.

Precision (pr), recall (rc), and the resulting *F*_max_ are defined as

$$\begin{array}{@{}rcl@{}} \text{pr}(\tau) &=& \frac{1}{m(\tau)}\sum\limits_{i=1}^{m(\tau)} \frac{{\sum\nolimits}_{f} \mathbbm{1}\left(f \in P_{i}(\tau) \wedge f \in T_{i}\right)}{\sum_{f} \mathbbm{1}\left(f \in P_{i}(\tau) \right)},\\ \text{rc}(\tau) &=& \frac{1}{n_{e}}\sum\limits_{i=1}^{n_{e}} \frac{{\sum\nolimits}_{f} \mathbbm{1}\left(f \in {P}_{i}(\tau) \wedge f \in T_{i}\right)}{{\sum\nolimits}_{f} \mathbbm{1}\left(f \in T_{i} \right)}, \\ F_{\max} &=& \max_{\tau} \left\{ \frac{2\cdot \text{pr}(\tau)\cdot \text{rc}(\tau)}{\text{pr}(\tau) + \text{rc}(\tau)} \right\}, \end{array} $$

where *P*_*i*_(*τ*) denotes the set of terms that have predicted scores greater than or equal to *τ* for a protein sequence *i*, *T*_*i*_ denotes the corresponding ground-truth set of terms for that sequence, *m*(*τ*) is the number of sequences with at least one predicted score greater than or equal to *τ*, $\mathbbm {1}\left (\cdot \right)$ is an indicator function, and *n*_*e*_ is the number of targets used in a particular mode of evaluation. In the full evaluation mode *n*_*e*_=*n*, the number of benchmark proteins, whereas in the partial evaluation mode *n*_*e*_=*m*(0), i.e., the number of proteins that were chosen to be predicted using the particular method. For each method, we refer to *m*(0)/*n* as the *coverage* because it provides the fraction of benchmark proteins on which the method made any predictions.

The remaining uncertainty (ru), misinformation (mi), and the resulting minimum semantic distance (*S*_min_) are defined as

$$\begin{array}{@{}rcl@{}} \text{ru}(\tau) &=& \frac{1}{n_{e}}\sum\limits_{i=1}^{n_{e}} \sum\limits_{f} \text{ic}(f) \cdot \mathbbm{1}\left(f \notin P_{i}(\tau) \wedge f \in T_{i} \right),\\ \text{mi}(\tau) &=& \frac{1}{n_{e}}\sum\limits_{i=1}^{n_{e}} \sum\limits_{f} \text{ic}(f) \cdot \mathbbm{1}\left(f \in P_{i}(\tau) \wedge f \notin T_{i} \right), \\ S_{\min} &=& \min_{\tau}\left\{ \sqrt{\text{ru}(\tau)^{2} + \text{mi}(\tau)^{2}} \right\}, \end{array} $$

where ic(*f*) is the information content of the ontology term *f* [10]. It is estimated in a maximum likelihood manner as the negative binary logarithm of the conditional probability that the term *f* is present in a protein’s annotation given that all its parent terms are also present. Note that here, *n*_*e*_=*n* in the full evaluation mode and *n*_*e*_=*m*(0) in the partial evaluation mode applies to both ru and mi.

In addition to the main metrics, we used two secondary metrics. Those were the weighted version of the precision–recall curves and the version of the remaining uncertainty–misinformation curves normalized to the [ 0,1] interval. These metrics and the corresponding evaluation results are shown in Additional file 1.

For the term-centric evaluation we used the area under the receiver operating characteristic (ROC) curve (AUC). The AUCs were calculated for all terms that have acquired at least ten positively annotated sequences, whereas the remaining benchmarks were used as negatives. The term-centric evaluation was used both for ranking models and to differentiate well and poorly predictable terms. The performance of each model on each term is provided in Additional file 1.

As we required all methods to keep two significant figures for prediction scores, the threshold *τ* in all metrics used in this study was varied from 0.01 to 1.00 with a step size of 0.01.

### Data sets

Protein function annotations for the GO assessment were extracted, as a union, from three major protein databases that are available in the public domain: Swiss-Prot [11], UniProt-GOA [12] and the data from the GO consortium web site [3]. We used evidence codes EXP, IDA, IPI, IMP, IGI, IEP, TAS, and IC to build benchmark and ground-truth sets. Annotations for the HPO assessment were downloaded from the HPO database [8].

**Fig. 2:**
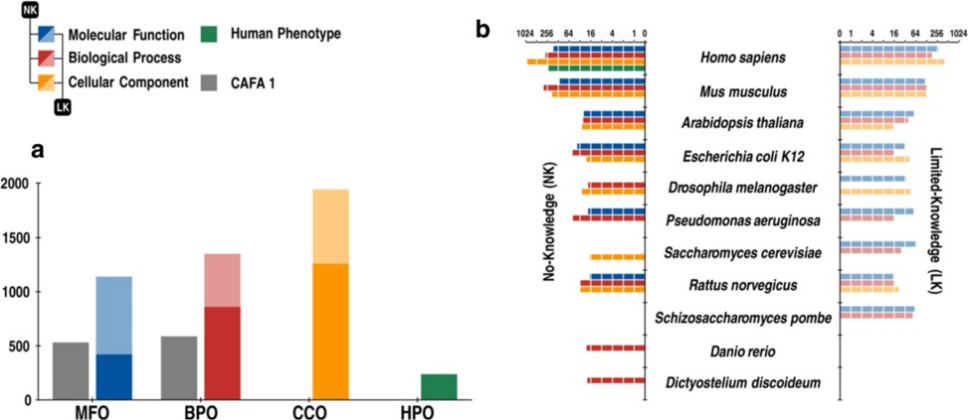
CAFA2 benchmark breakdown. **a** The benchmark size for each of the four ontologies. **b** Breakdown of benchmarks for both types over 11 species (with no less than 15 proteins) sorted according to the total number of benchmark proteins. For both panels, *dark colors* (*blue*, *red*, and *yellow*) correspond to no-knowledge (NK) types, while their *light color* counterparts correspond to limited-knowledge (LK) types. The distributions of information contents corresponding to the benchmark sets are shown in Additional file 1. The size of CAFA 1 benchmarks are shown in *gray*. *BPO* Biological Process Ontology, *CCO* Cellular Component Ontology, *HPO* Human Phenotype Ontology, *LK* limited-knowledge, *MFO* Molecular Function Ontology, *NK* no-knowledge

Figure 2 summarizes the benchmarks we used in this study. Figure 2a shows the benchmark sizes for each of the ontologies and compares these numbers to CAFA1. All species that have at least 15 proteins in any of the benchmark categories are listed in Fig. 2b.

### Comparison between CAFA1 and CAFA2 methods

We compared the results from CAFA1 and CAFA2 using a benchmark set that we created from CAFA1 targets and CAFA2 targets. More precisely, we used the stored predictions of the target proteins from CAFA1 and compared them with the new predictions from CAFA2 on the overlapping set of CAFA2 benchmarks and CAFA1 targets (a sequence had to be a no-knowledge target in both experiments to be eligible for this evaluation). For this analysis only, we used an artificial GO version by taking the intersection of the two GO snapshots (versions from January 2011 and June 2013) so as to mitigate the influence of ontology changes. We, thus, collected 357 benchmark proteins for MFO comparisons and 699 for BPO comparisons. The two baseline methods were trained on respective Swiss-Prot annotations for both ontologies so that they serve as controls for database change. In particular, SwissProt2011 (for CAFA1) contained 29,330 and 31,282 proteins for MFO and BPO, while SwissProt2014 (for CAFA2) contained 26,907 and 41,959 proteins for the two ontologies.

To conduct a head-to-head analysis between any two methods, we generated *B*=10,000 bootstrap samples and let methods compete on each such benchmark set. The performance improvement *δ* from CAFA1 to CAFA2 was calculated as

$$\begin{array}{@{}rcl@{}} \delta(m_{2}, m_{1}) = \frac{1}{B}\sum_{b=1}^{B} F_{\max}^{(b)}(m_{2}) - \frac{1}{B}\sum_{b=1}^{B} F_{\max}^{(b)}(m_{1}), \end{array} $$

where *m*_1_ and *m*_2_ stand for methods from CAFA1 and CAFA2, respectively, and $F_{\max }^{(b)}(\cdot)$ represents the *F*_max_ of a method evaluated on the *b*-th bootstrapped benchmark set.

### Baseline models

We built two baseline methods, Naïve and BLAST, and compared them with all participating methods. The Naïve method simply predicts the frequency of a term being annotated in a database [13]. BLAST was based on search results using the Basic Local Alignment Search Tool (BLAST) software against the training database [14]. A term will be predicted as the highest local alignment sequence identity among all BLAST hits annotated with the term. Both of these methods were trained on the experimentally annotated proteins available in Swiss-Prot at time *t*_0_, except for HPO where the two baseline models were trained using the annotations from the *t*_0_ release of the HPO.

## Results and discussion

### Top methods have improved since CAFA1

We conducted the second CAFA experiment 3 years after the first one. As our knowledge of protein function has increased since then, it was worthwhile to assess whether computational methods have also been improved and if so, to what extent. Therefore, to monitor the progress over time, we revisit some of the top methods in CAFA1 and compare them with their successors.

**Fig. 3:**
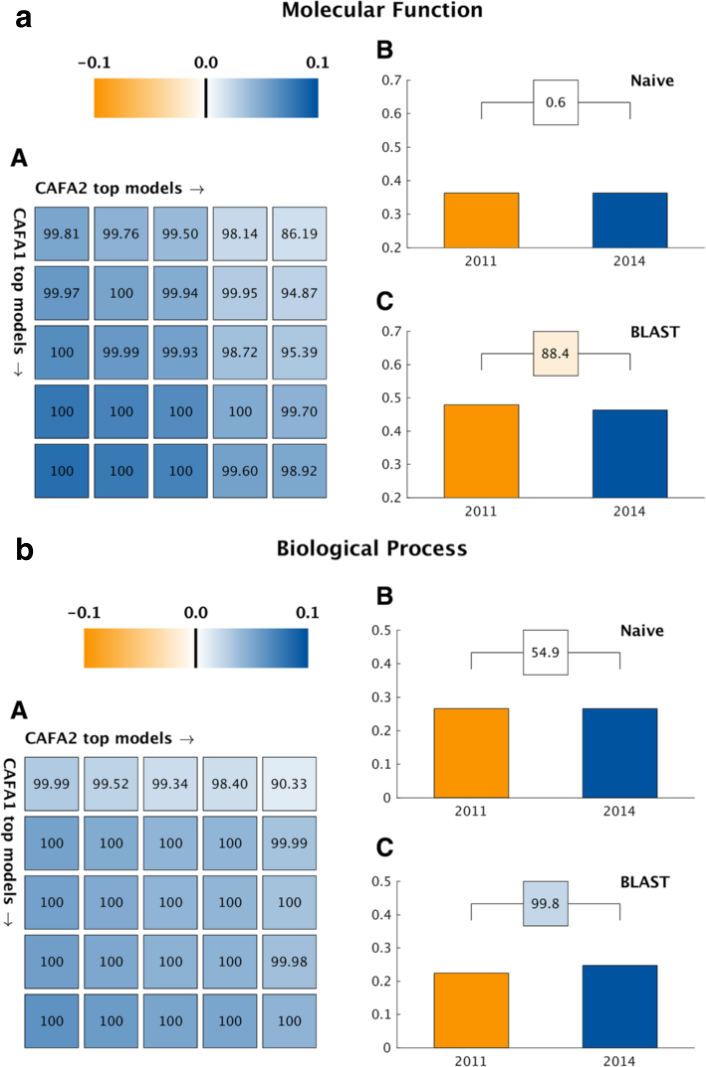
CAFA1 versus CAFA2 (*top methods*). A comparison in *F*_max_ between the top-five CAFA1 models against the top-five CAFA2 models. *Colored boxes* encode the results such that (1) the colors indicate margins of a CAFA2 method over a CAFA1 method in *F*_max_ and (2) the numbers in the *box* indicate the percentage of wins. For both the Molecular Function Ontology (**a**) and Biological Process Ontology (**b**) results: **A** CAFA1 top-five models (*rows, from top to bottom*) against CAFA2 top-five models (*columns, from left to right*). **B** Comparison of Naïve baselines trained respectively on SwissProt2011 and SwissProt2014. **C** Comparison of BLAST baselines trained on SwissProt2011 and SwissProt2014

For each benchmark set we carried out a bootstrap-based comparison between a pair of top-ranked methods (one from CAFA1 and another from CAFA2), as described in “Methods”. The average performance metric as well as the number of wins were recorded (in the case of identical performance, neither method was awarded a win). Figure 3 summarizes the results of this analysis. We use a color code from orange to blue to indicate the performance improvement *δ* from CAFA1 to CAFA2.

The selection of top methods for this study was based on their performance in each ontology on the entire benchmark sets. Panels B and C in Fig. 3 compare baseline methods trained on different data sets. We see no improvements of these baselines except for BLAST on BPO where it is slightly better to use the newer version of Swiss-Prot as the reference database for the search. On the other hand, all top methods in CAFA2 outperformed their counterparts in CAFA1. For predicting molecular functions, even though transferring functions from BLAST hits does not give better results, the top models still managed to perform better. It is possible that the newly acquired annotations since CAFA1 enhanced BLAST, which involves direct function transfer, and perhaps lead to better performances of those downstream methods that rely on sequence alignments. However, this effect does not completely explain the extent of the performance improvement achieved by those methods. This is promising evidence that top methods from the community have improved since CAFA1 and that improvements were not simply due to updates of curated databases.

### Protein-centric evaluation

**Fig. 4:**
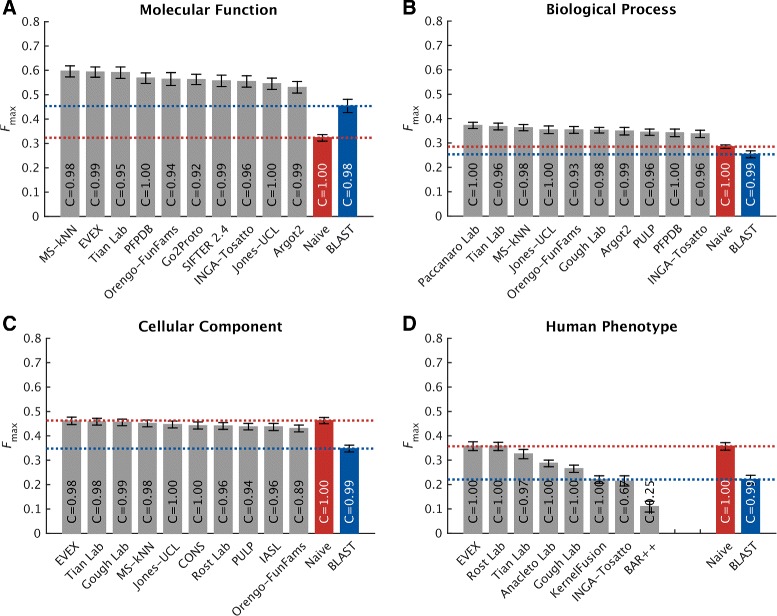
Overall evaluation using the maximum *F* measure, *F*_max_. Evaluation was carried out on no-knowledge benchmark sequences in the full mode. The coverage of each method is shown within its performance bar. A perfect predictor would be characterized with *F*_max_=1. Confidence intervals (95 %) were determined using bootstrapping with 10,000 iterations on the set of benchmark sequences. For cases in which a principal investigator participated in multiple teams, the results of only the best-scoring method are presented. Details for all methods are provided in Additional file 1

**Fig. 5:**
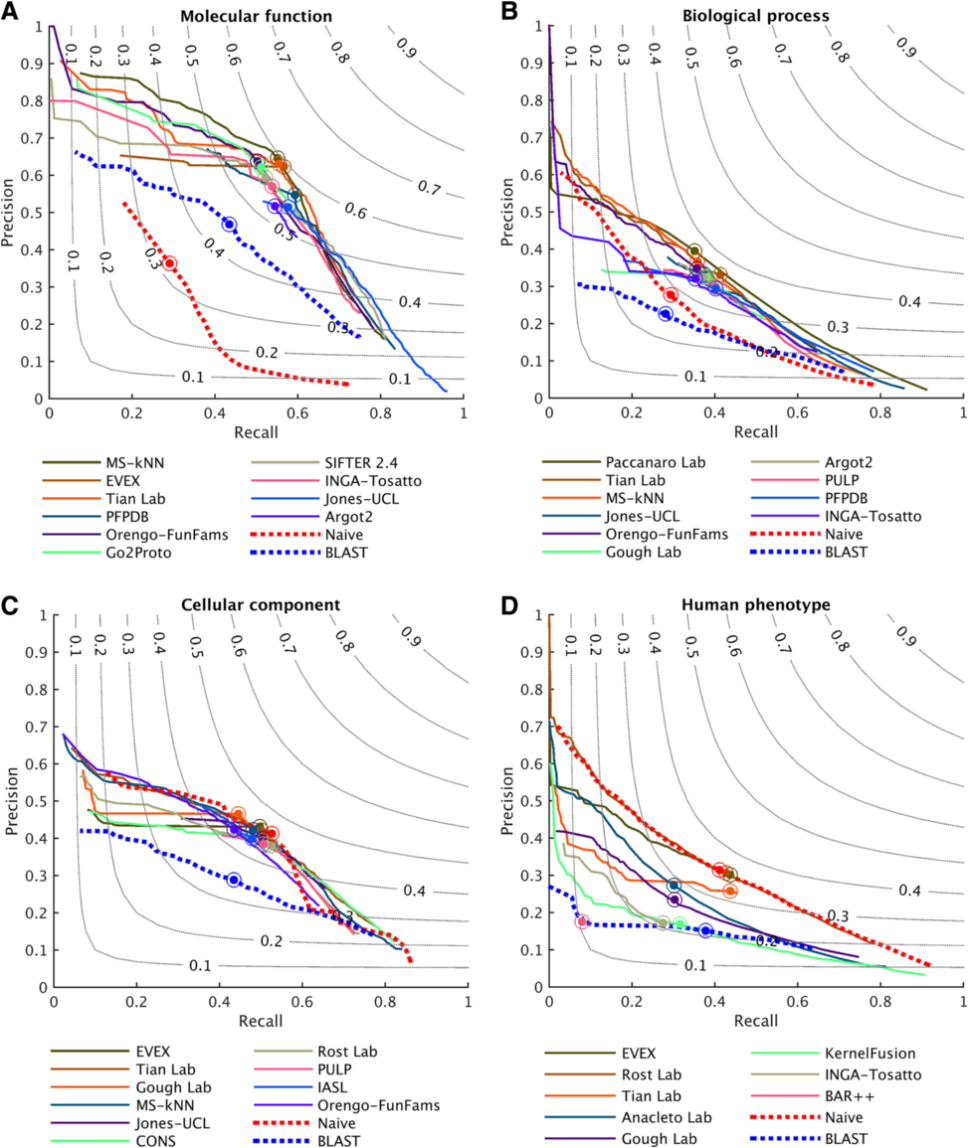
Precision–recall curves for top-performing methods. Evaluation was carried out on no-knowledge benchmark sequences in the full mode. A perfect predictor would be characterized with *F*_max_=1, which corresponds to the point (1,1) in the precision–recall plane. For cases in which a principal investigator participated in multiple teams, the results of only the best-scoring method are presented

**Fig. 6:**
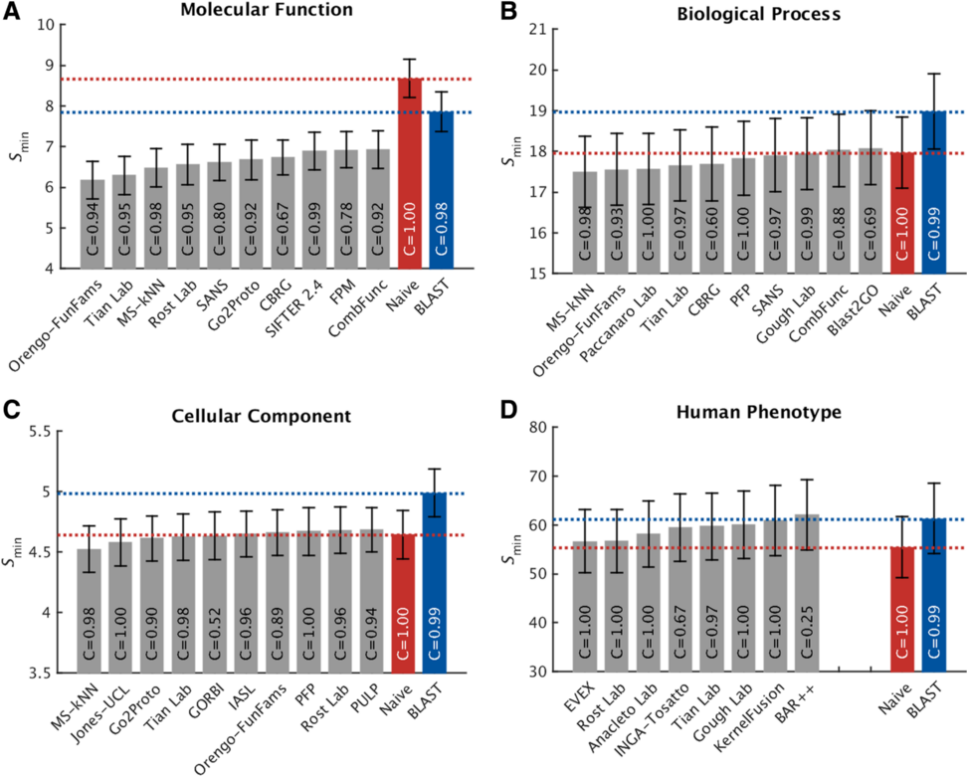
Overall evaluation using the minimum semantic distance, *S*_min_. Evaluation was carried out on no-knowledge benchmark sequences in the full mode. The coverage of each method is shown within its performance bar. A perfect predictor would be characterized with *S*_min_=0. Confidence intervals (95 %) were determined using bootstrapping with 10,000 iterations on the set of benchmark sequences. For cases in which a principal investigator participated in multiple teams, the results of only the best-scoring method are presented. Details for all methods are provided in Additional file 1

Protein-centric evaluation measures how accurately methods can assign functional terms to a protein. The protein-centric performance evaluation of the top-ten methods is shown in Figs. 4, 5, and 6. The 95 % confidence intervals were estimated using bootstrapping on the benchmark set with *B*=10,000 iterations [15]. The results provide a broad insight into the state of the art.

Predictors performed very differently across the four ontologies. Various reasons contribute to this effect including: (1) the topological properties of the ontology such as the size, depth, and branching factor; (2) term predictability; for example, the BPO terms are considered to be more abstract in nature than the MFO and CCO terms; (3) the annotation status, such as the size of the training set at *t*_0_, the annotation depth of benchmark proteins, as well as various annotation biases [6].

In general, CAFA2 methods perform better at predicting MFO terms than any other ontology. Top methods achieved *F*_max_ scores around 0.6 and considerably surpassed the two baseline models. Maintaining the pattern from CAFA1, the performance accuracies in the BPO category were not as good as in the MFO category. The best-performing method scored slightly below 0.4.

For the two newly added ontologies in CAFA2, we observed that the top predictors performed no better than the Naïve method under *F*_max_, whereas they slightly outperformed the Naïve method under *S*_min_ in CCO. One reason for the competitive performance of the Naïve method in the CCO category is that a small number of relatively general terms are frequently used, and those relative frequencies do not diffuse quickly enough with the depth of the graph. For instance, the annotation frequency of “organelle” (GO:0043226, level 2), “intracellular part” (GO:0044424, level 3), and “cytoplasm” (GO:0005737, level 4) are all above the best threshold for the Naïve method (*τ*_optimal_=0.32). Correctly predicting these terms increases the number of true positives and thus boosts the performance of the Naïve method under the *F*_max_ evaluation. However, once the less informative terms are down-weighted (using the *S*_min_ measure), the Naïve method becomes significantly penalized and degraded. Another reason for the comparatively good performance of Naïve is that the benchmark proteins were annotated with more general terms than the (training) proteins previously deposited in the UniProt database. This effect was most prominent in the CCO (Additional file 1: Figure S2) and has thus artificially boosted the performance of the Naïve method. The weighted *F*_max_ and normalized *S*_min_ evaluations can be found in Additional file 1.

Interestingly, generally shallower annotations of benchmark proteins do not seem to be the major reason for the observed performance in the HPO category. One possibility for the observed performance is that, unlike for GO terms, the HPO annotations are difficult to transfer from other species. Another possibility is the sparsity of experimental annotations. The current number of experimentally annotated proteins in HPO is 4794, i.e., 0.5 proteins per HPO term, which is at least an order of magnitude less than for other ontologies. Finally, the relatively high frequency of general terms may have also contributed to the good performance of Naïve. We originally hypothesized that a possible additional explanation for this effect might be that the average number of HPO terms associated with a human protein is considerably larger than in GO; i.e., the mean number of annotations per protein in HPO is 84, while for MFO, BPO, and CCO, the mean number of annotations per protein is 10, 39, and 14, respectively. However, we do not observe this effect in other ontologies when the benchmark proteins are split into those with a low or high number of terms. Overall, successfully predicting the HPO terms in the protein-centric mode is a difficult problem and further effort will be required to fully characterize the performance.

### Term-centric evaluation

The protein-centric view, despite its power in showing the strengths of a predictor, does not gauge a predictor’s performance for a specific function. In a term-centric evaluation, we assess the ability of each method to identify new proteins that have a particular function, participate in a process, are localized to a component, or affect a human phenotype. To assess this term-wise accuracy, we calculated AUCs in the prediction of individual terms. Averaging the AUC values over terms provides a metric for ranking predictors, whereas averaging predictor performance over terms provides insights into how well this term can be predicted computationally by the community.

**Fig. 7:**
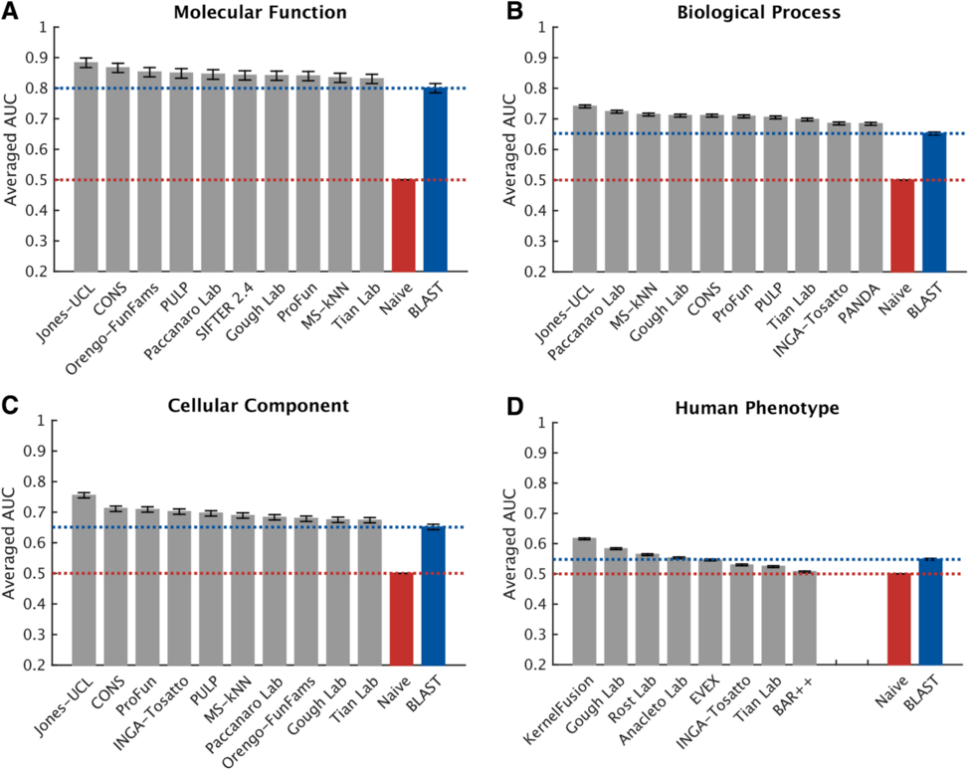
Overall evaluation using the averaged AUC over terms with no less than ten positive annotations. The evaluation was carried out on no-knowledge benchmark sequences in the full mode. Error bars indicate the standard error in averaging AUC over terms for each method. For cases in which a principal investigator participated in multiple teams, the results of only the best-scoring method are presented. Details for all methods are provided in Additional file 1. *AUC* receiver operating characteristic curve

**Fig. 8:**
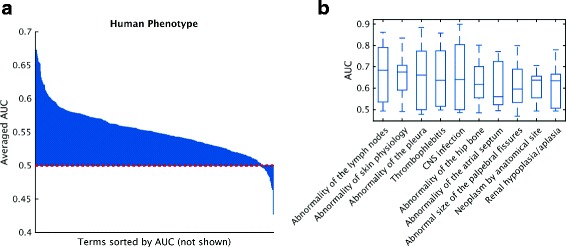
Averaged AUC per term for Human Phenotype Ontology. **a** Terms are sorted based on AUC. The *dashed red line* indicates the performance of the Naïve method. **b** The top-ten accurately predicted terms without overlapping ancestors (except for the root). *AUC* receiver operating characteristic curve

Figure 7 shows the performance evaluation where the AUCs for each method were averaged over all terms for which at least ten positive sequences were available. Proteins without predictions were counted as predictions with a score of 0. As shown in Figs. 4, 5, and 6, correctly predicting CCO and HPO terms for a protein might not be an easy task according to the protein-centric results. However, the overall poor performance could also result from the dominance of poorly predictable terms. Therefore, a term-centric view can help differentiate prediction quality across terms. As shown in Fig. 8, most of the terms in HPO obtain an AUC greater than the Naïve model, with some terms on average achieving reasonably well AUCs around 0.7. Depending on the training data available for participating methods, well-predicted phenotype terms range from mildly specific such as “Lymphadenopathy” and “Thrombophlebitis” to general ones such as “Abnormality of the Skin Physiology”.

### Performance on various categories of benchmarks

#### Easy versus difficult benchmarks

As in CAFA1, the no-knowledge GO benchmarks were divided into easy versus difficult categories based on their maximal global sequence identity with proteins in the training set. Since the distribution of sequence identities roughly forms a bimodal shape (Additional file 1), a cutoff of 60 % was manually chosen to define the two categories. The same cutoff was used in CAFA1. Unsurprisingly, across all three ontologies, the performance of the BLAST model was substantially impacted for the difficult category because of the lack of high sequence identity homologs and as a result, transferring annotations was relatively unreliable. However, we also observed that most top methods were insensitive to the types of benchmarks, which provides us with encouraging evidence that state-of-the-art protein function predictors can successfully combine multiple potentially unreliable hits, as well as multiple types of data, into a reliable prediction.

#### Species-specific categories

**Fig. 9:**
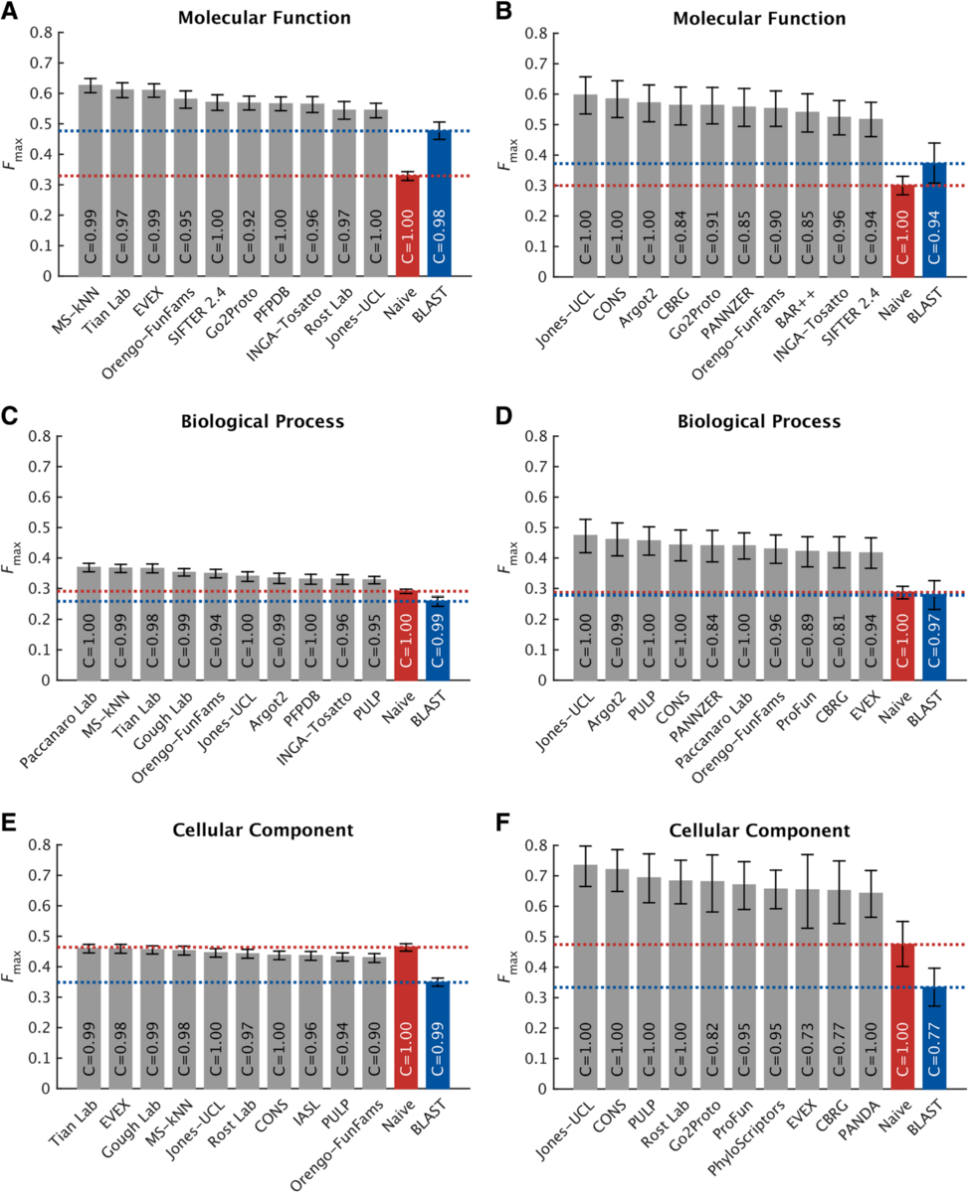
Performance evaluation using the maximum *F* measure, *F*_max_, on eukaryotic (*left*) versus prokaryotic (*right*) benchmark sequences. The evaluation was carried out on no-knowledge benchmark sequences in the full mode. The coverage of each method is shown within its performance bar. Confidence intervals (95 %) were determined using bootstrapping with 10,000 iterations on the set of benchmark sequences. For cases in which a principal investigator participated in multiple teams, the results of only the best-scoring method are presented. Details for all methods are provided in Additional file 1

The benchmark proteins were split into even smaller categories for each species as long as the resulting category contained at least 15 sequences. However, because of space limitations, in Fig. 9 we show the breakdown results on only eukarya and prokarya benchmarks; the species-specific results are provided in Additional file 1. It is worth noting that the performance accuracies on the entire benchmark sets were dominated by the targets from eukarya due to their larger proportion in the benchmark set and annotation preferences. The eukarya benchmark rankings therefore coincide with the overall rankings, but the smaller categories typically showed different rankings and may be informative to more specialized research groups.

For all three GO ontologies, no-knowledge prokarya benchmark sequences collected over the annotation growth phase mostly (over 80 %) came from two species: *Escherichia coli* and *Pseudomonas aeruginosa* (for CCO, 21 out of 22 proteins were from *E. coli*). Thus, one should keep in mind that the prokarya benchmarks essentially reflect the performance on proteins from these two species. Methods predicting the MFO terms for prokaryotes are slightly worse than those for eukaryotes. In addition, direct function transfer by homology for prokaryotes did not work well using this ontology. However, the performance was better using the other two ontologies, especially CCO. It is not very surprising that the top methods achieved good performance for *E. coli* as it is a well-studied model organism.

### Diversity of predictions

**Fig. 10:**
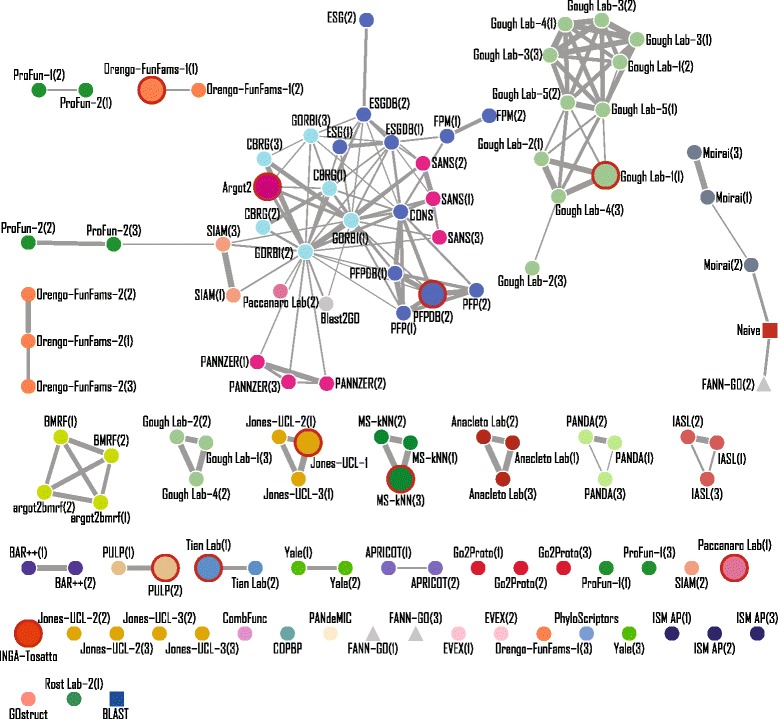
Similarity network of participating methods for BPO. Similarities are computed as Pearson’s correlation coefficient between methods, with a 0.75 cutoff for illustration purposes. A unique color is assigned to all methods submitted under the same principal investigator. Not evaluated (organizers’) methods are shown in *triangles*, while benchmark methods (Naïve and BLAST) are shown in *squares*. The top-ten methods are highlighted with enlarged nodes and *circled in red*. The edge width indicates the strength of similarity. Nodes are labeled with the name of the methods followed by “-team(model)” if multiple teams/models were submitted

Evaluation of the top methods revealed that performance was often statistically indistinguishable between the best methods. This could result from all top methods making the same predictions, or from different prediction sets resulting in the same summarized performance. To assess this, we analyzed the extent to which methods generated similar predictions within each ontology. Specifically, we calculated the pairwise Pearson correlation between methods on a common set of gene-concept pairs and then visualized these similarities as networks (for BPO, see Fig. 10; for MFO, CCO, and HPO, see Additional file 1).

In MFO, where we observed the highest overall performance of prediction methods, eight of the ten top methods were in the largest connected component. In addition, we observed a high connectivity between methods, suggesting that the participating methods are leveraging similar sources of data in similar ways. Predictions for BPO showed a contrasting pattern. In this ontology, the largest connected component contained only two of the top-ten methods. The other top methods were contained in components made up of other methods produced by the same lab. This suggests that the approaches that participating groups have taken generate more diverse predictions for this ontology and that there are many different paths to a top-performing biological process prediction method. Results for HPO were more similar to those for BPO, while results for cellular component were more similar in structure to molecular function.

Taken together, these results suggest that ensemble approaches that aim to include independent sources of high-quality predictions may benefit from leveraging the data and techniques used by different research groups and that such approaches that effectively weigh and integrate disparate methods may demonstrate more substantial improvements over existing methods in the process and phenotype ontologies where current prediction approaches share less similarity.

At the time that authors submitted predictions, we also asked them to select from a list of 30 keywords that best describe their methodology. We examined these author-assigned keywords for methods that ranked in the top ten to determine what approaches were used in currently high-performing methods (Additional file 1). Sequence alignment and machine-learning methods were in the top-three terms for all ontologies. For biological process, the other member of the top three is protein–protein interactions, while for cellular component and molecular function the third member is sequence properties. The broad sets of keywords among top-performing methods further suggest that these methods are diverse in their inputs and approach.

### Case study: ADAM-TS12

To illustrate some of the challenges and accomplishments of CAFA, we provide an in-depth examination of the prediction of the functional terms of one protein, human ADAM-TS12 [16]. ADAMs (a disintegrin and metalloproteinase) are a family of secreted metallopeptidases featuring a pro-domain, a metalloproteinase, a disintegrin, a cysteine-rich epidermal growth-factor-like domain, and a transmembrane domain [17]. The ADAM-TS subfamily include eight thrombospondin type-1 (TS-1) motifs; it is believed to play a role in fetal pulmonary development and may have a role as a tumor suppressor, specifically the negative regulation of the hepatocyte growth factor receptor signaling pathway [18].

**Fig. 11:**
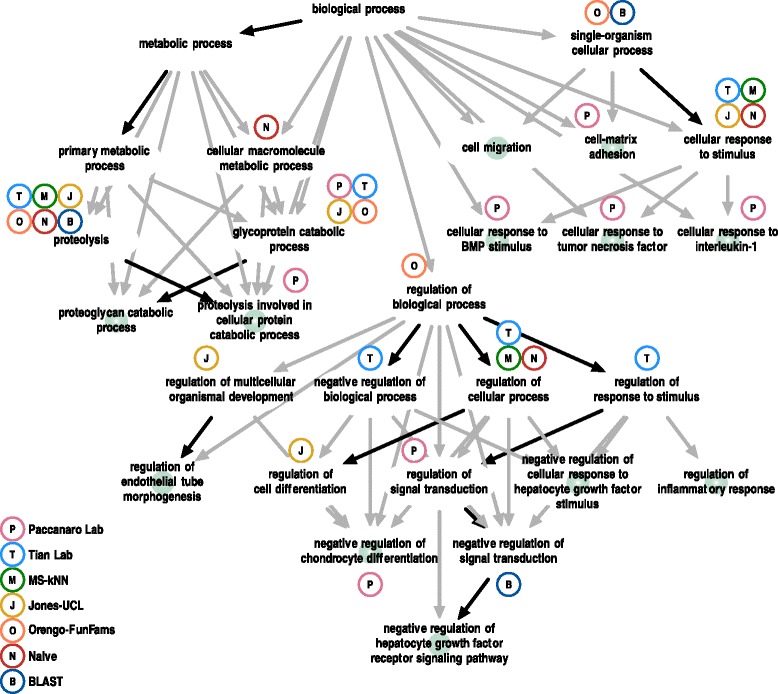
Case study on the human *ADAM-TS12* gene. Biological process terms associated with *ADAM-TS12* gene in the union of the three databases by September 2014. The entire functional annotation of *ADAM-TS12* consists of 89 terms, 28 of which are shown. Twelve terms, marked in *green*, are leaf terms. This directed acyclic graph was treated as ground truth in the CAFA2 assessment. *Solid black lines* provide direct “is a” or “part of” relationships between terms, while *gray lines* mark indirect relationships (that is, some terms were not drawn in this picture). Predicted terms of the top-five methods and two baseline methods were picked at their optimal *F*_max_ threshold. Over-predicted terms are not shown

We did not observe any experimental annotation by the time submission was closed. Annotations were later deposited to all three GO ontologies during the growth phase of CAFA2. Therefore, ADAM-TS12 was considered a no-knowledge benchmark protein for our assessment in all GO ontologies. The total number of leaf terms to predict for biological process was 12; these nodes induced a directed acyclic annotation graph consisting of 89 nodes. In Fig. 11 we show the performance of the top-five methods in predicting the BPO terms that are experimentally verified to be associated with ADAM-TS12.

As can be seen, most methods correctly discovered non-leaf nodes with a moderate amount of information content. “Glycoprotein Catabolic Process”, “Cellular Response to Stimulus”, and “Proteolysis” were the best discovered GO terms by the top-five performers. The Paccanaro Lab (P) discovered several additional correct leaf terms. It is interesting to note that only BLAST successfully predicted “Negative regulation of signal transduction” whereas the other methods did not. The reason for this is that we set the threshold for reporting a discovery when the confidence score for a term was equal to or exceeded the method’s *F*_max_. In this particular case, the Paccanaro Lab method did predict the term, but the confidence score was 0.01 below their *F*_max_ threshold.

This example illustrates both the success and the difficulty of correctly predicting highly specific terms in BPO, especially with a protein that is involved in four distinct cellular processes: in this case, regulation of cellular growth, proteolysis, cellular response to various cytokines, and cell-matrix adhesion. Additionally, this example shows that the choices that need to be made when assessing method performance may cause some loss of information with respect to the method’s actual performance. That is, the way we capture a method’s performance in CAFA may not be exactly the same as a user may employ. In this case, a user may choose to include lower confidence scores when running the Paccanaro Lab method, and include the term “Negative regulation of signal transduction” in the list of accepted predictions.

## Conclusions

Accurately annotating the function of biological macromolecules is difficult, and requires the concerted effort of experimental scientists, biocurators, and computational biologists. Though challenging, advances are valuable: accurate predictions allow biologists to rapidly generate testable hypotheses about how proteins fit into processes and pathways. We conducted the second CAFA challenge to assess the status of the computational function prediction of proteins and to quantify the progress in the field.

### The field has moved forward

Three years ago, in CAFA1, we concluded that the top methods for function prediction outperform straightforward function transfer by homology. In CAFA2, we observe that the methods for function prediction have improved compared to those from CAFA1. As part of the CAFA1 experiment, we stored all predictions from all methods on 48,298 target proteins from 18 species. We compared those stored predictions to the newly deposited predictions from CAFA2 on the overlapping set of benchmark proteins and CAFA1 targets. The head-to-head comparisons among the top-five CAFA1 methods against the top-five CAFA2 methods reveal that the top CAFA2 methods outperformed all top CAFA1 methods.

Our parallel evaluation using an unchanged BLAST algorithm with data from 2011 and data from 2014 showed little difference, strongly suggesting that the improvements observed are due to methodological advances. The lessons from CAFA1 and annual AFP-SIG during the ISMB conference, where new developments are rapidly disseminated, may have contributed to this outcome [19].

### Evaluation metrics

A universal performance assessment in protein function prediction is far from straightforward. Although various evaluation metrics have been proposed under the framework of multi-label and structured-output learning, the evaluation in this subfield also needs to be interpretable to a broad community of researchers as well as the public. To address this, we used several metrics in this study as each provides useful insights and complements the others. Understanding the strengths and weaknesses of current metrics and developing better metrics remain important.

One important observation with respect to metrics is that the protein-centric and term-centric views may give different perspectives to the same problem. For example, while in MFO and BPO we generally observe a positive correlation between the two, in CCO and HPO these different metrics may lead to entirely different interpretations of an experiment. Regardless of the underlying cause, as discussed in “Results and discussion”, it is clear that some ontological terms are predictable with high accuracy and can be reliably used in practice even in these ontologies. In the meantime, more effort will be needed to understand the problems associated with the statistical and computational aspects of method development.

### Well-performing methods

We observe that participating methods usually specialize in one or few categories of protein function prediction, and have been developed with their own application objectives in mind. Therefore, the performance rankings of methods often change from one benchmark set to another. There are complex factors that influence the final ranking including the selection of the ontology, types of benchmark sets and evaluation, as well as evaluation metrics, as discussed earlier. Most of our assessment results show that the performances of top-performing methods are generally comparable to each other. It is worth noting that performance is usually better in predicting molecular function than other ontologies.

Beyond simply showing diversity in inputs, our evaluation of prediction similarity revealed that many top-performing methods are reaching this status by generating distinct predictions, suggesting that there is additional room for continued performance improvement. Although a small group of methods could be considered as generally high performing, there is no single method that dominates over all benchmarks. Taken together, these results highlight the potential for ensemble learning approaches in this domain.

We also observed that when provided with a chance to select a reliable set of predictions, the methods generally perform better (partial evaluation mode versus full evaluation mode). This outcome is encouraging; it suggests that method developers can predict where their methods are particularly accurate and target them to that space.

Our keyword analysis showed that machine-learning methods are widely used by successful approaches. Protein interactions were more overrepresented in the best-performing methods for biological process prediction. This suggests that predicting membership in pathways and processes requires information on interacting partners in addition to a protein’s sequence features.

### Final notes

Automated functional annotation remains an exciting and challenging task, central to understanding genomic data, which are central to biomedical research. Three years after CAFA1, the top methods from the community have shown encouraging progress. However, in terms of raw scores, there is still significant room for improvement in all ontologies, and particularly in BPO, CCO, and HPO. There is also a need to develop an experiment-driven, as opposed to curation-driven, component of the evaluation to address limitations for term-centric evaluation. In the future CAFA experiments, we will continue to monitor the performance over time and invite a broad range of computational biologists, computer scientists, statisticians, and others to address these engaging problems of concept annotation for biological macromolecules through CAFA.

CAFA2 significantly expanded the number of protein targets, the number of biomedical ontologies used for annotation, the number of analysis scenarios, as well as the metrics used for evaluation. The results of the CAFA2 experiment detail the state of the art in protein function prediction, can guide the development of new concept annotation methods, and help molecular biologists assess the relative reliability of predictions. Understanding the function of biological macromolecules brings us closer to understanding life at the molecular level and improving human health.

## Additional file

Additional file 1 A document containing a subset of CAFA2 analyses that are equivalent to those provided about the CAFA1 experiment in the CAFA1 supplement. (PDF 11100 kb)
