# Supplementary material for: An expanded evaluation of protein function prediction methods shows an improvement in accuracy
Source: Genome Biol. 2016 Sep 7;17(1):184. doi: 10.1186/s13059-016-1037-6 (PMC5015320; doi:10.1186/s13059-016-1037-6)
Supplement: Additional file 1 — A document containing a subset of CAFA2 analyses that are equivalent to those provided about the CAFA1 experiment in the CAFA1 supplement. (PDF 11100 kb) [file 13059_2016_1037_MOESM1_ESM.pdf]

Supplementary Information

"An expanded evaluation of protein function prediction methods shows an improvement in accuracy" by Jiang Y. *et al*

Genome Biology, 2015

Content:

- Supplementary Figures.
  1. Benchmark annotation depth distribution.
  2. Benchmark information content distribution.
  3. Benchmark sequence identity distribution.
  4. Top predictors, precision-recall curves.
  5. Top predictors, easy vs. difficult, precision-recall curves.
  6. Top predictors, eukarya vs. prokarya, precision-recall curves.
  7. Top predictors, species breakdown, $F_{\max}$ bars.
  8. Top predictors, weighted precision-recall curves.
  9. Top predictors, normalized remaining uncertainty-misinformation.
  10. Similarity networks between methods.
  11. Keyword usage by top methods.
- Supplementary Table 1. Participating teams.

Additional supplementary data (297MB) provides all additional data, analyses and full prediction results for every method. It is available at:

`https://dx.doi.org/10.6084/m9.figshare.2059944.v1`

Code used in CAFA2 is available at:

`https://github.com/yuxjiang/CAFA2`

Supplementary Figure 1 Distribution of depths of the leaf annotations, over all benchmarks in (A) Molecular Function ontology, (B) Biological Process ontology, (C) Cellular Component ontology and (D) Human Phenotype ontology. A leaf term for a benchmark protein is defined as any term whose descendant nodes (more specific nodes) are not among the experimentally determined terms for that protein.

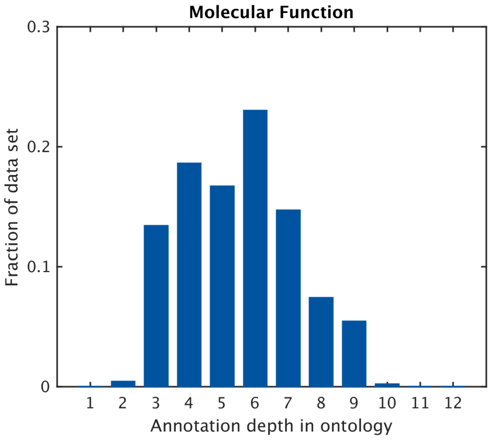

(A)

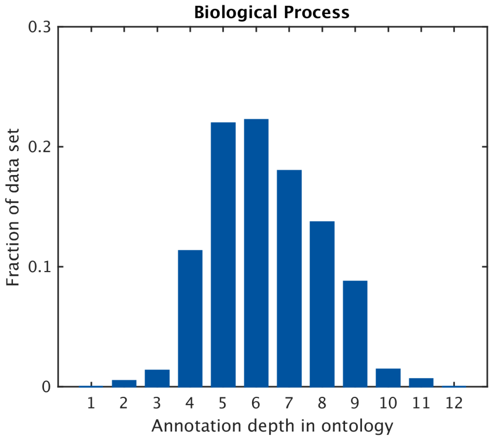

(B)

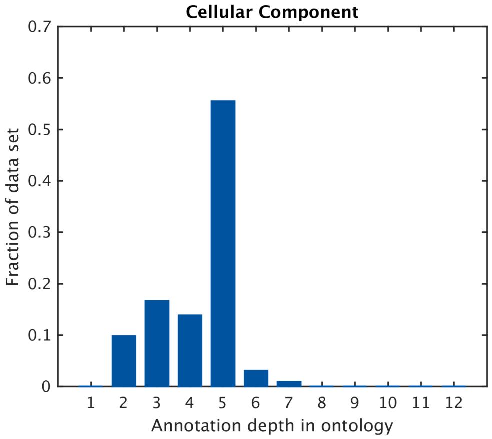

(C)

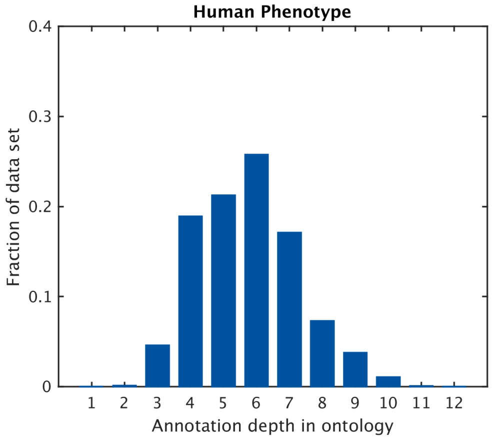

(D)

Supplementary Figure 2 The histogram and boxplot of total information content of benchmark proteins as well as all experimentally annotated proteins at time t_1; i.e., the point of benchmark collection: (A) Molecular Function ontology, (B) Biological Process ontology, (C) Cellular Component ontology, and (D) Human Phenotype ontology. The information content of each directed acyclic graph was calculated according to [9]. The red point in each plot indicates the value of information content for the predicted annotation corresponding to the Naive baseline model.

Supplementary Figure 2A:

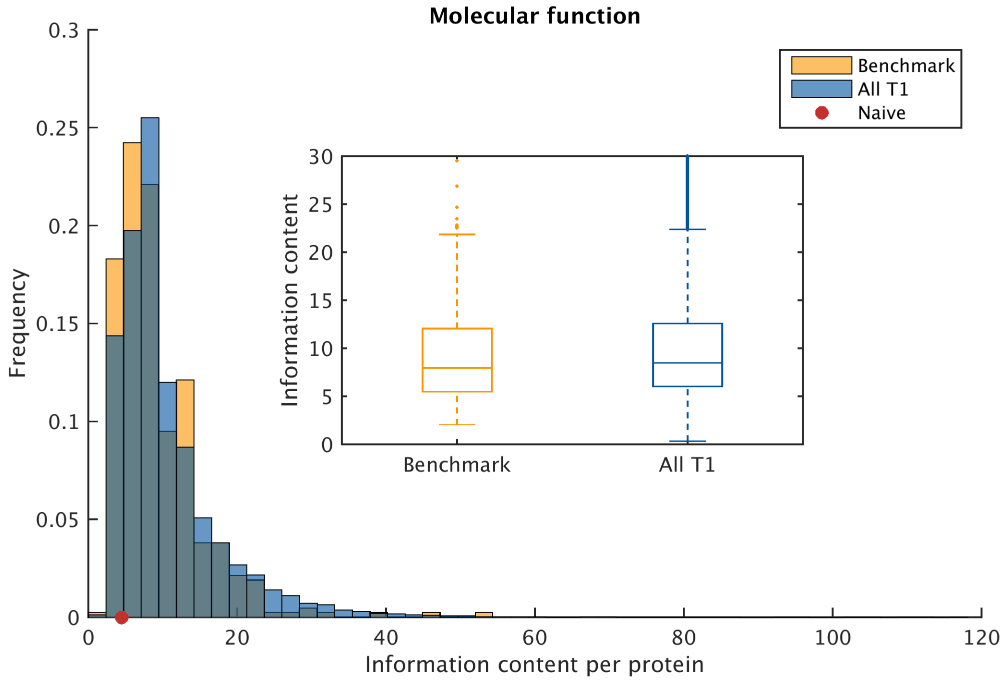

Supplementary Figure 2B:

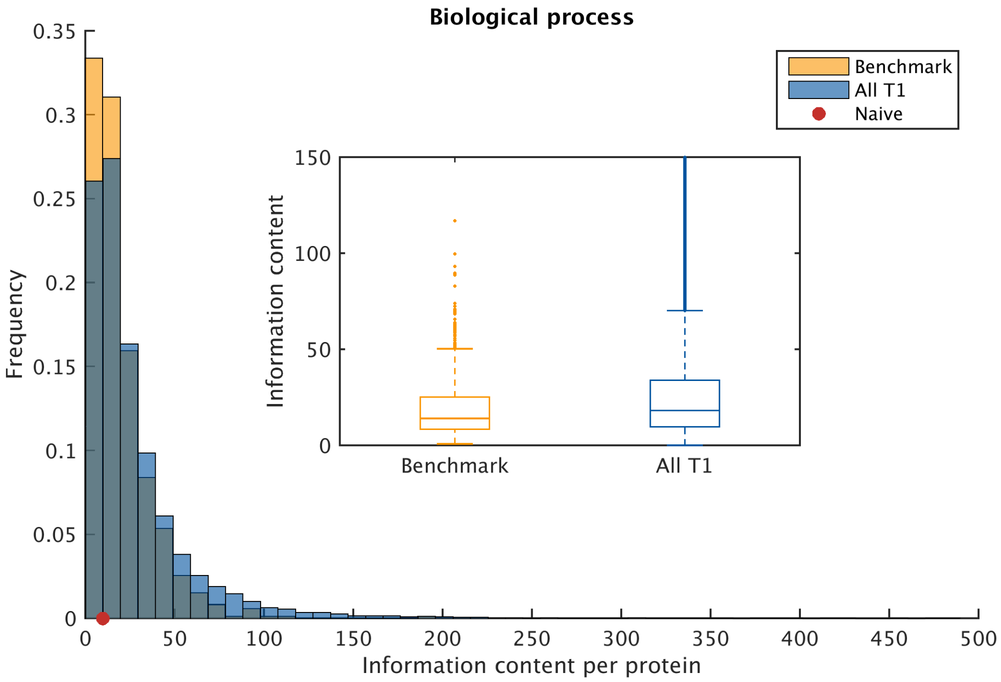

Supplementary Figure 2C:

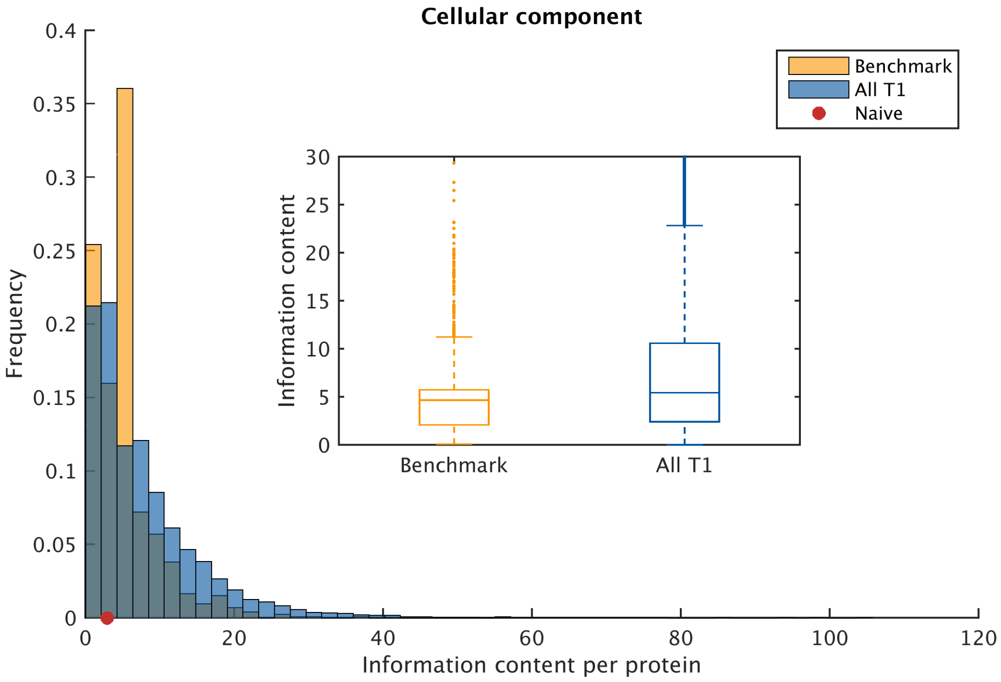

Supplementary Figure 2D:

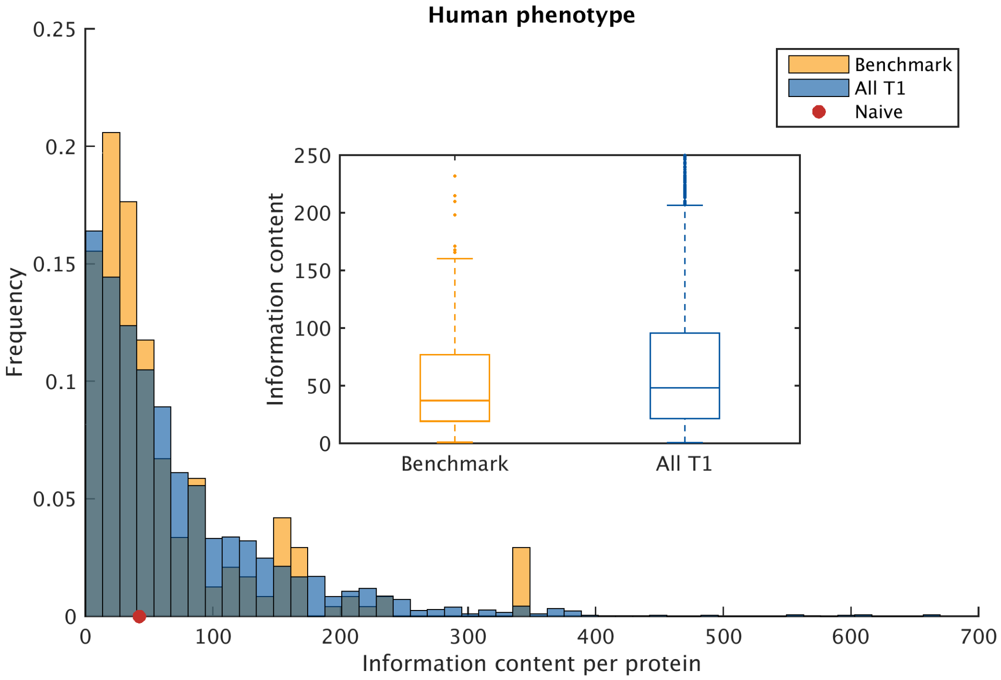

Supplementary Figure 3 The histogram of pairwise sequence identities between each benchmark proteins and the experimentally annotated template most similar to it: (A) Molecular Function ontology, (B) Biological Process ontology, and (C) Cellular Component ontology. The histograms roughly determine two groups of benchmarks: *easy* – with maximum global sequence identity greater than or equal to 60%, and *difficult* – with maximum global sequence identity below 60%.

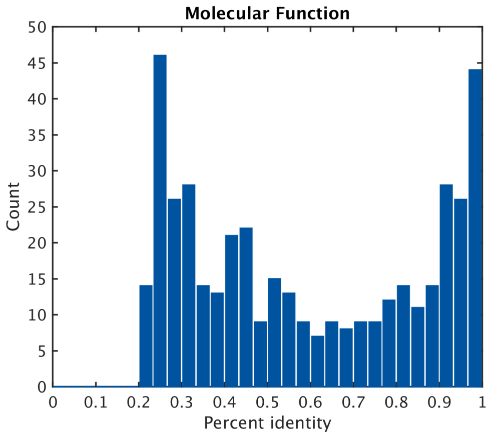

(A)

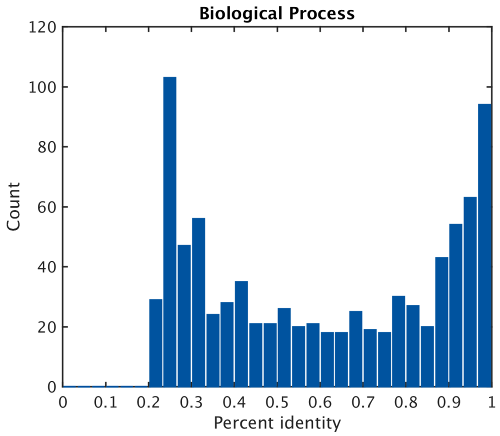

(B)

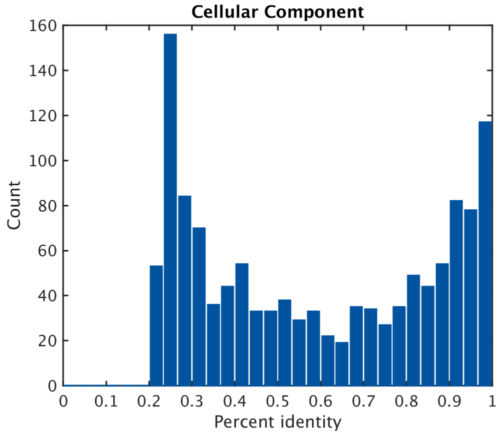

(C)

Supplementary Figure 4 Precision-recall curves for the top-performing methods for (A) Molecular Function ontology, (B) Biological Process ontology, (C) Cellular Component ontology and (D) Human Phenotype ontology. All panels show the top ten participating methods in each category, as well as the Naïve and BLAST baseline methods. Points corresponding to the maximum F-measure are marked in circles on each curve. The legend provides the maximum F-measure (F) and coverage (C) for all methods. In cases where a Principal Investigator (PI) participated with multiple teams, only the results of the best scoring method are presented.

Supplementary Figure 4A:

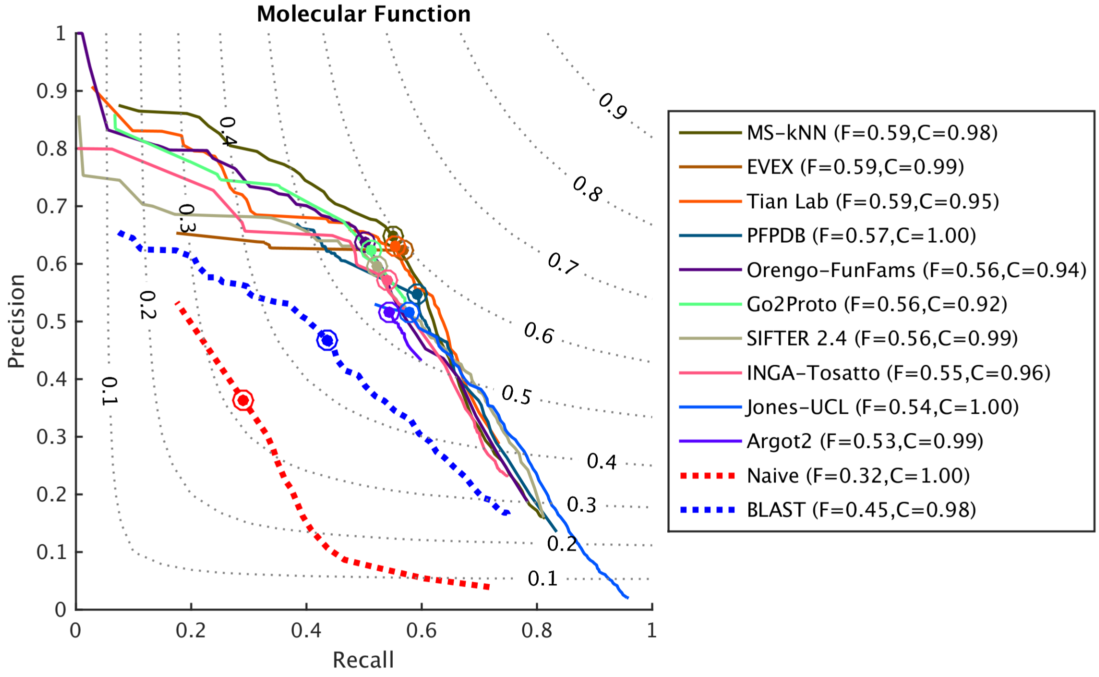

Supplementary Figure 4B:

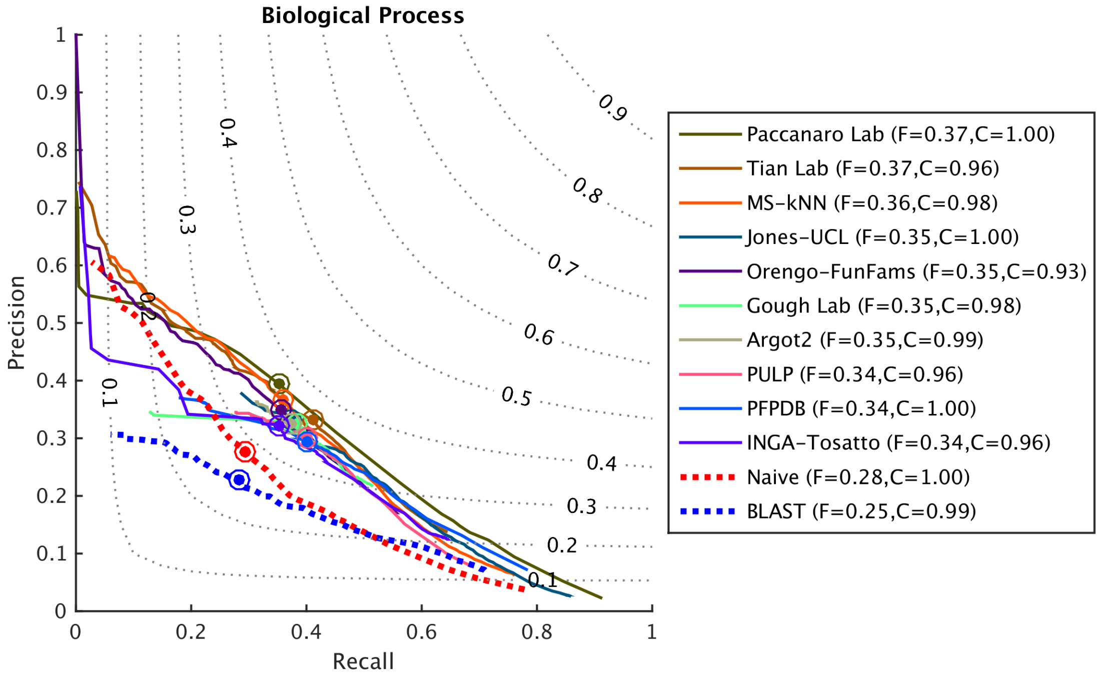

Supplementary Figure 4C:

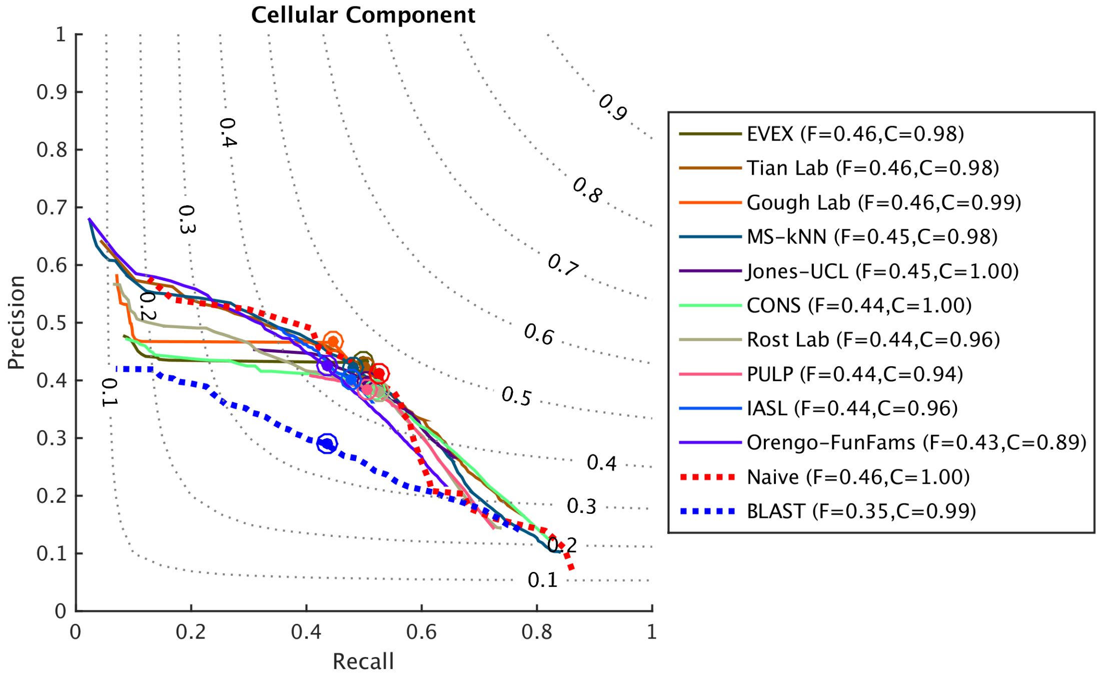

Supplementary Figure 4D:

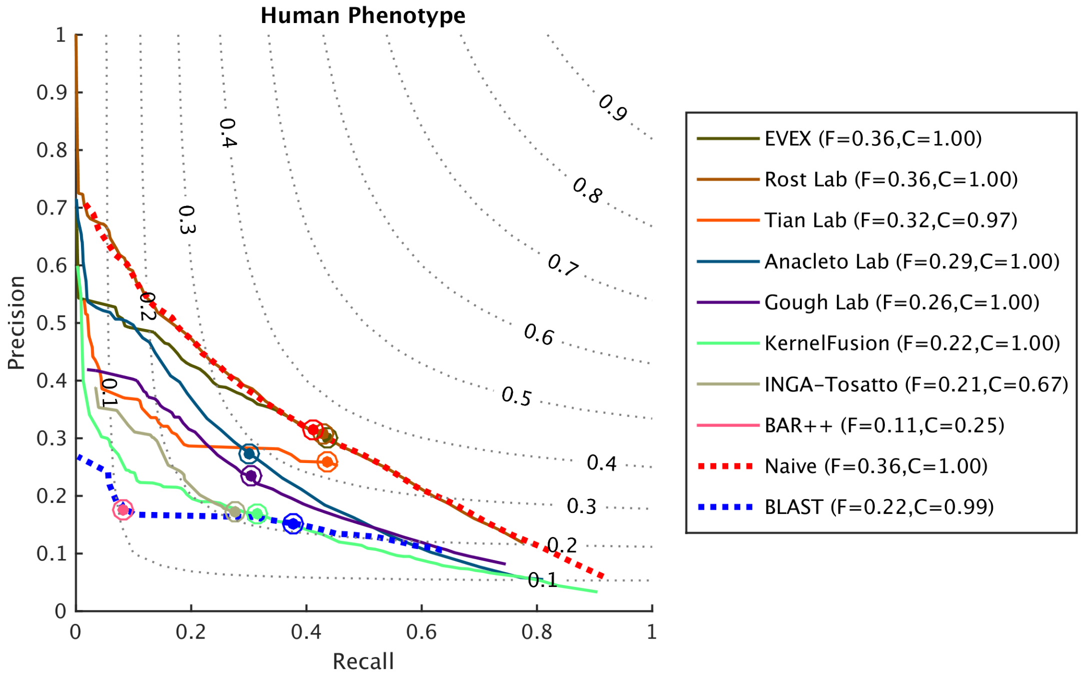

Supplementary Figure 5 Precision-recall curves for the top-performing methods for (A) easy benchmark category and Molecular Function ontology, (B) difficult benchmark category and Molecular Function ontology, (C) easy benchmark category and Biological Process ontology, (D) difficult benchmark category and Biological Process ontology, (E) easy benchmark category and Cellular Component ontology and (F) difficult benchmark category and Cellular Component ontology. All panels show the top ten participating methods in each category, as well as the Naïve and BLAST baseline methods. Points corresponding to the maximum F-measure are marked in circles on each curve. The legend provides the maximum F-measure (F) and coverage (C) for all methods. In cases where a Principal Investigator (PI) participated with multiple teams, only the results of the best scoring method are presented.

Supplementary Figure 5A (easy):

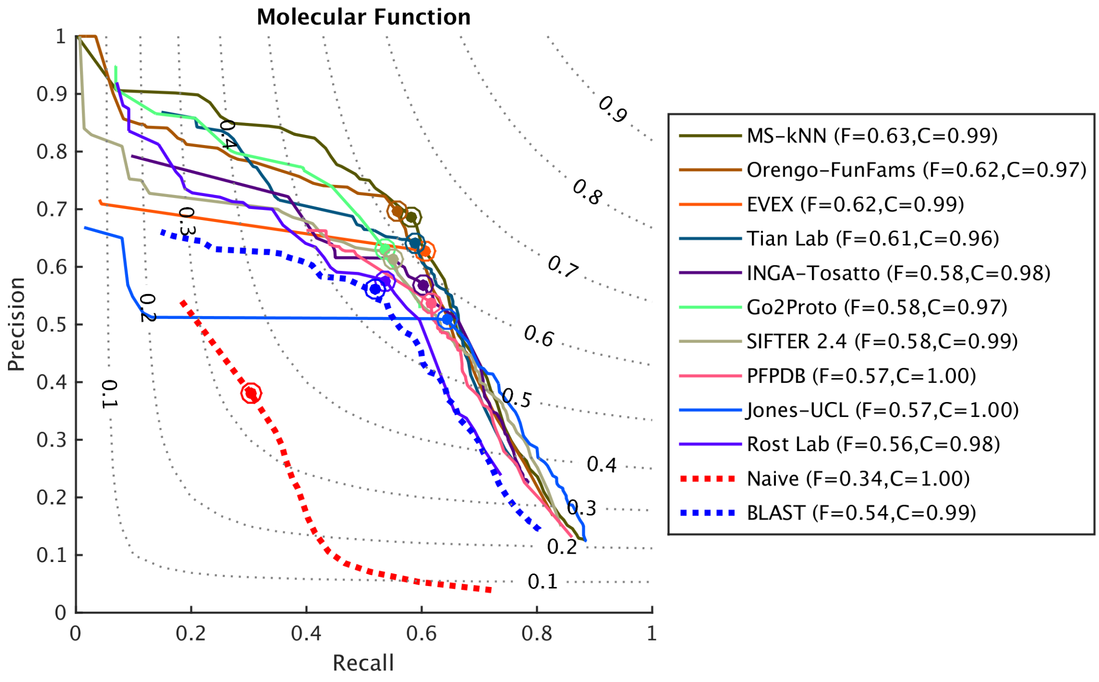

Supplementary Figure 5B (difficult):

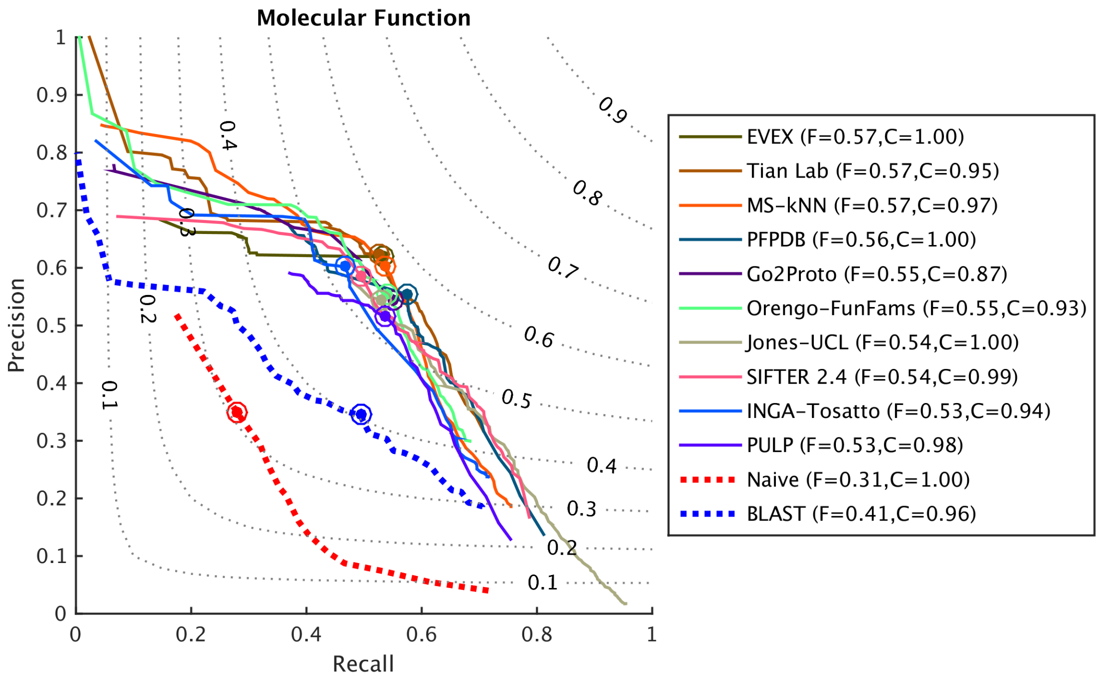

Supplementary Figure 5C (easy):

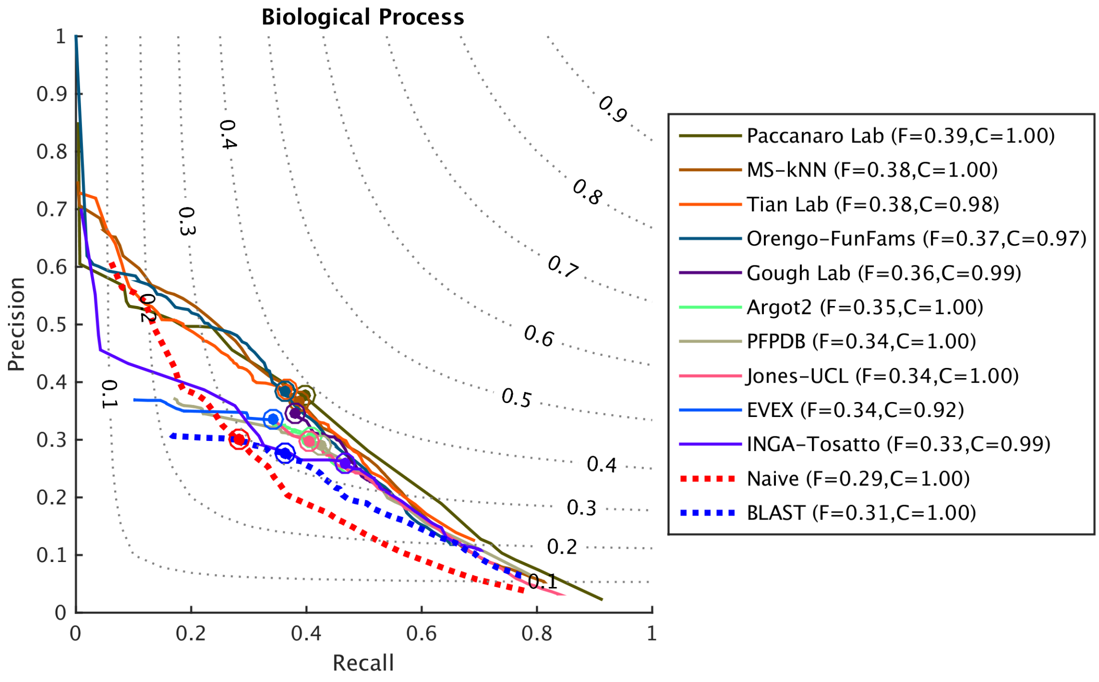

Supplementary Figure 5D (difficult):

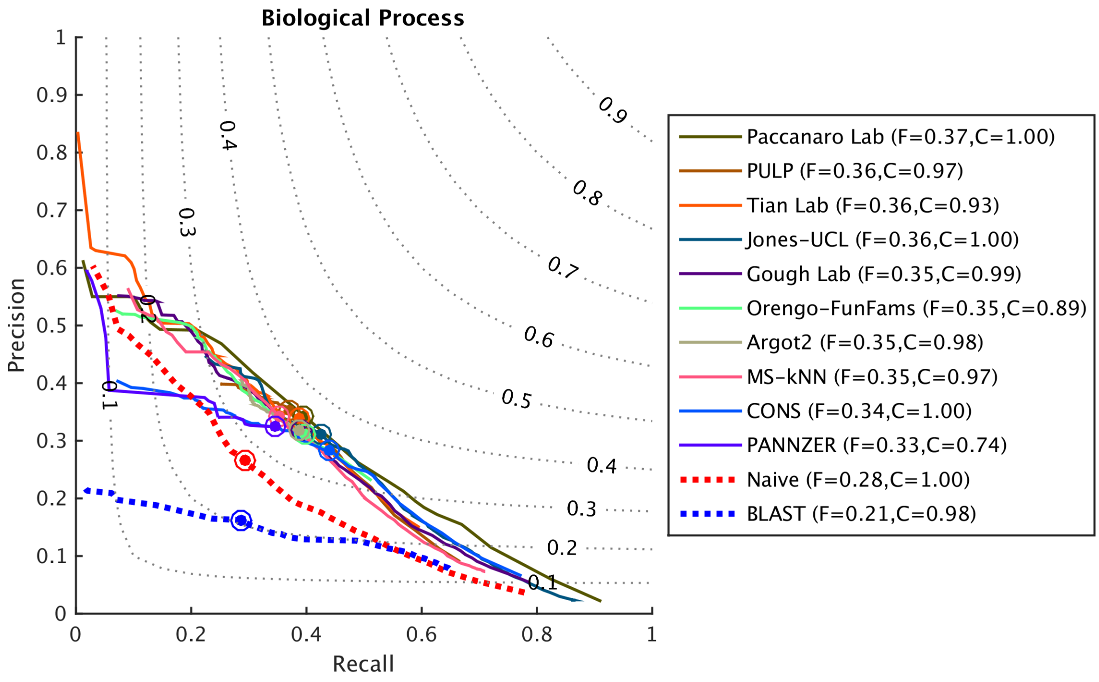

Supplementary Figure 5E (easy):

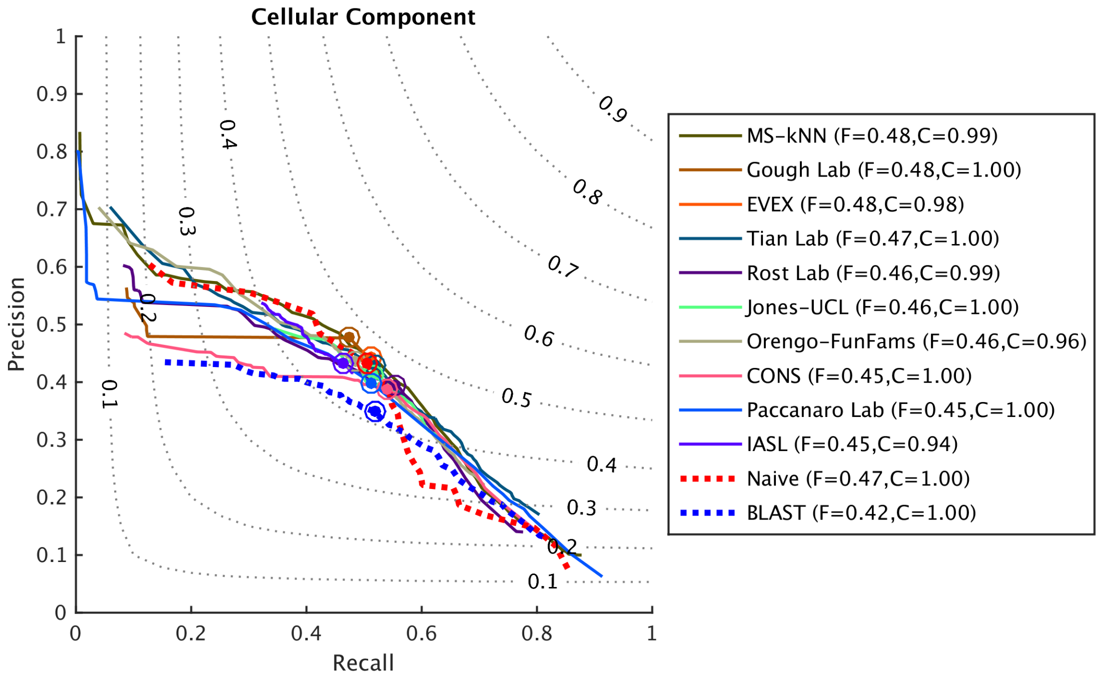

Supplementary Figure 5F (difficult):

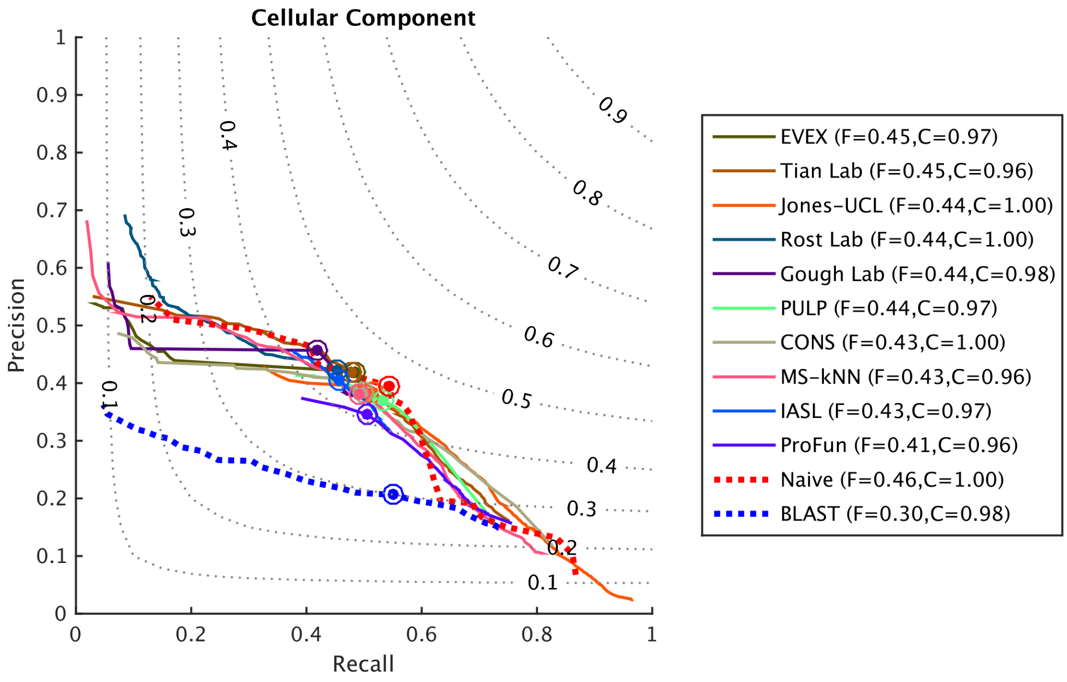

Supplementary Figure 6 Precision-recall curves for the top-performing methods for (A) eukaryotic benchmark category and Molecular Function ontology, (B) prokaryotic benchmark category and Molecular Function ontology, (C) eukaryotic benchmark category and Biological Process ontology, (D) prokaryotic benchmark category and Biological Process ontology, (E) eukaryotic benchmark category and Cellular Component ontology and (F) prokaryotic benchmark category and Cellular Component ontology. All panels show the top ten participating methods in each category, as well as the Naïve and BLAST baseline methods. Points corresponding to the maximum F-measure are marked in circles on each curve. The legend provides the maximum F-measure (F) and coverage (C) for all methods. In cases where a Principal Investigator (PI) participated with multiple teams, only the results of the best scoring method are presented.

Supplementary Figure 6A (eukarya):

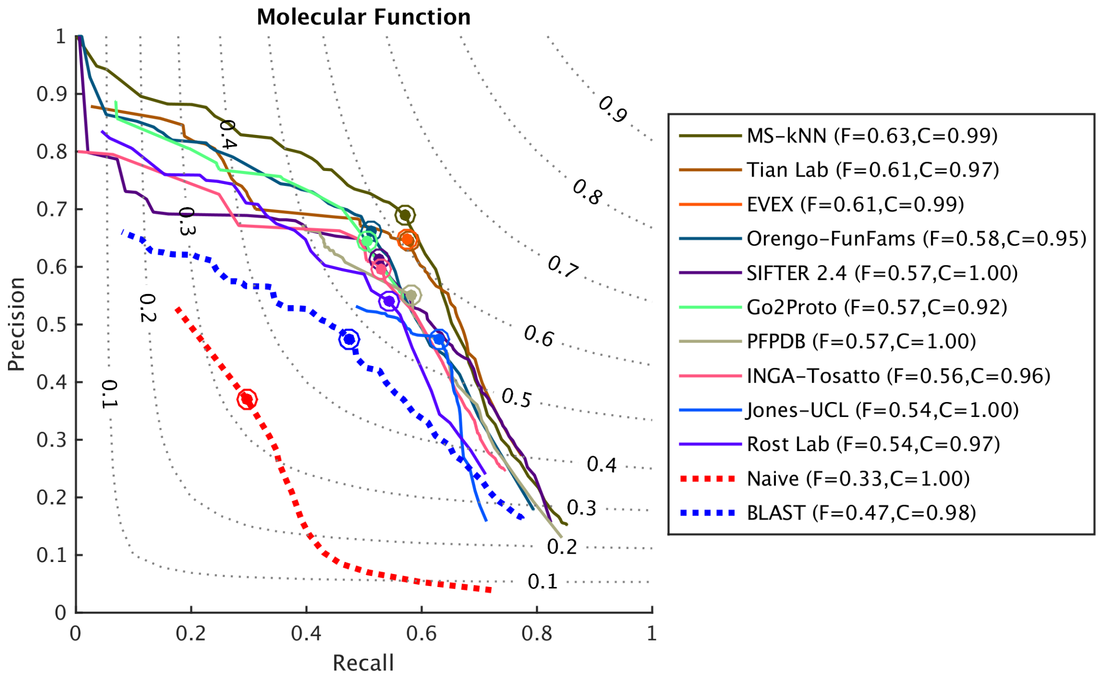

Supplementary Figure 6B (prokarya):

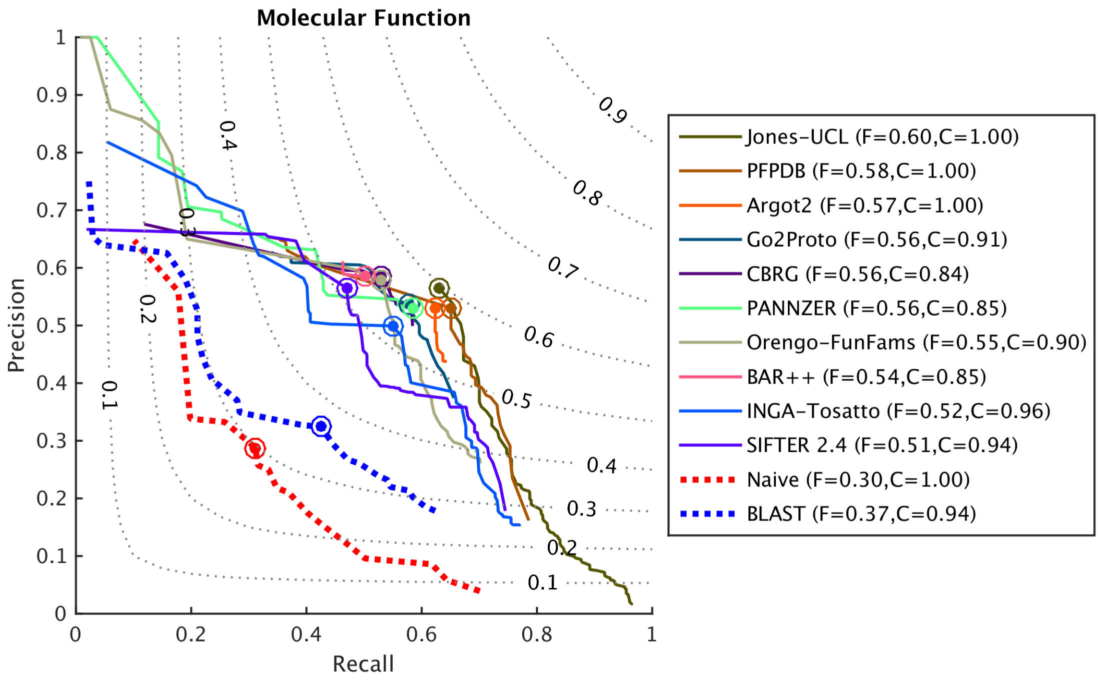

Supplementary Figure 6C (eukarya):

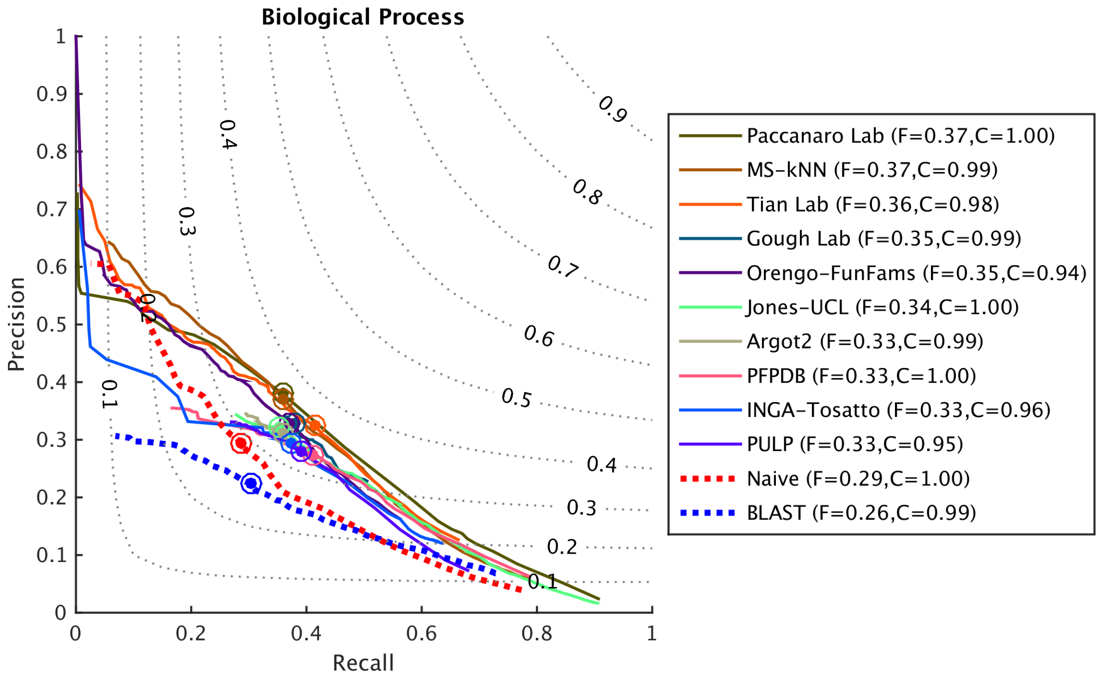

Supplementary Figure 6D (prokarya):

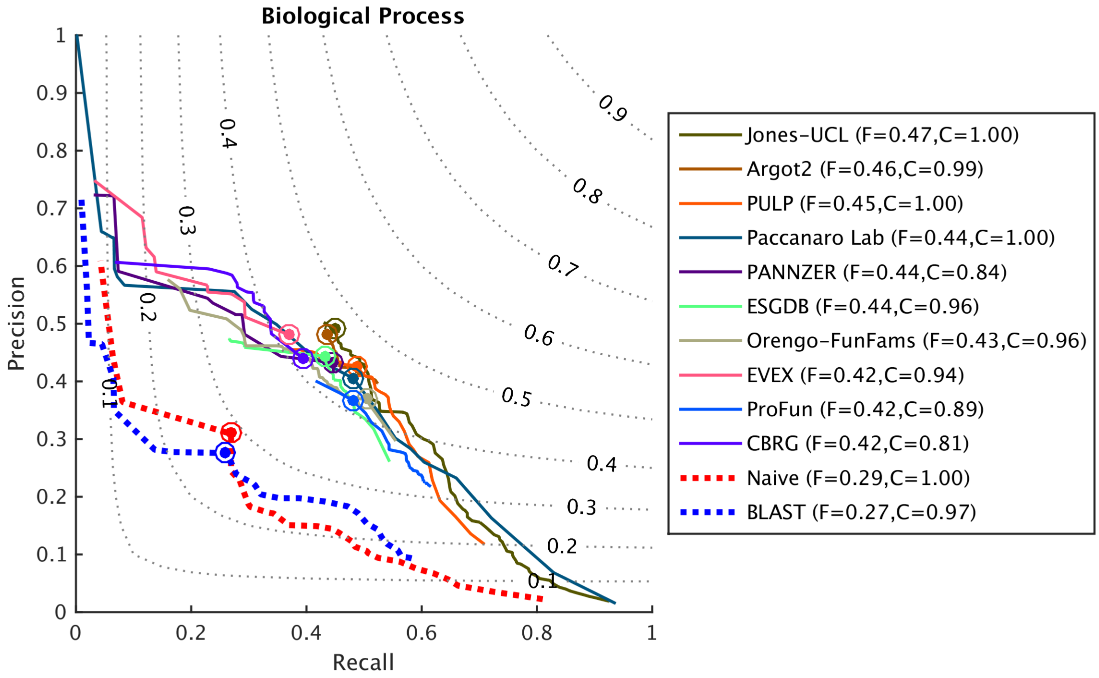

Supplementary Figure 6E (eukarya):

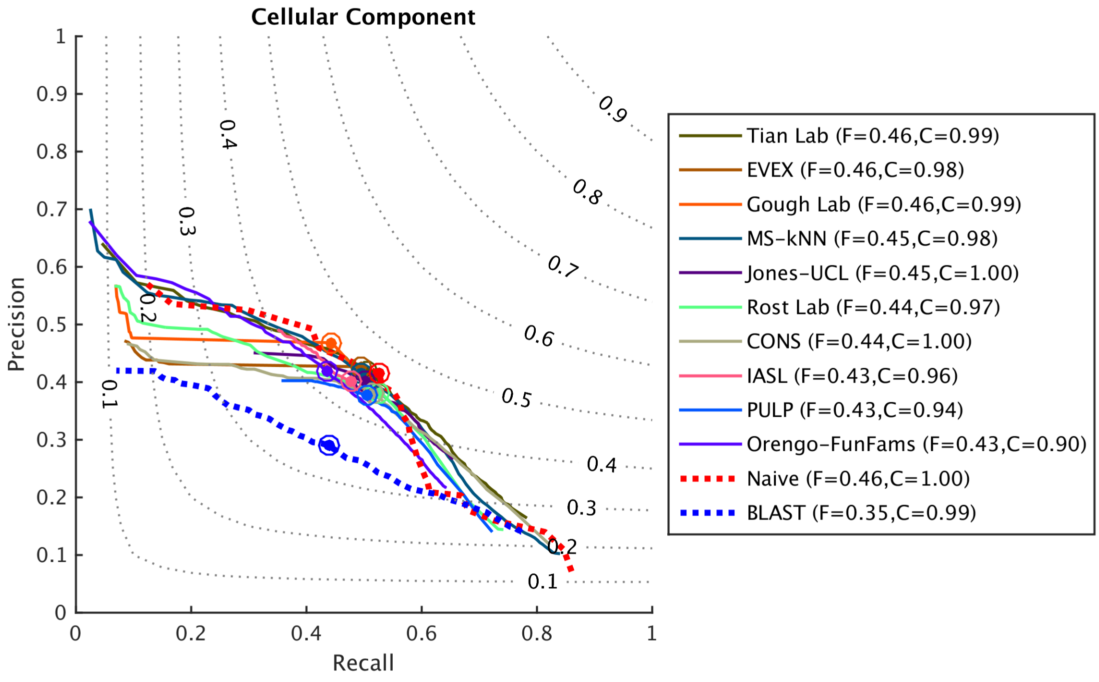

Supplementary Figure 6F (prokarya):

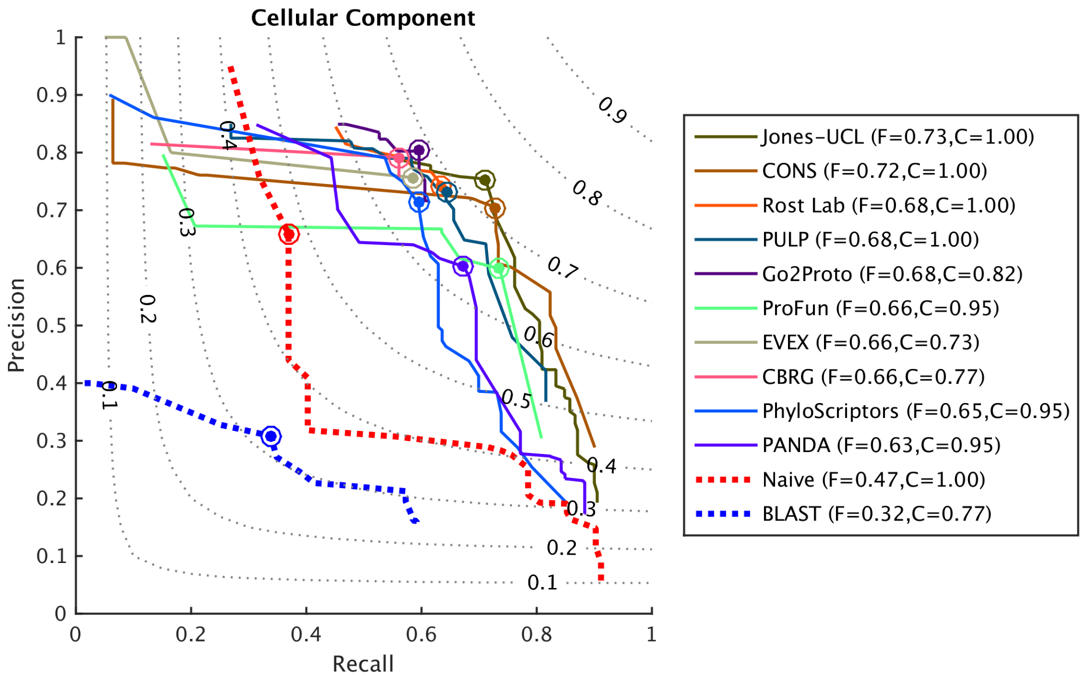

Supplementary Figure 7 Performance evaluation based on the maximum F-measure for the top-performing methods for the Molecular Function ontology (A–F), Biological Process ontology (G–O), and Cellular Component ontology (P–V). Only the species with 15 benchmark proteins or more are included. All bars show the top ten participating methods as well as the Naïve and BLAST baseline methods. A perfect predictor would be characterized with $F_{\max}$ of 1. Confidence interval (95%) were determined using bootstrapping with 10,000 iterations on the set of target sequences.

Supplementary Figure 7A (*Arabidopsis thaliana*):

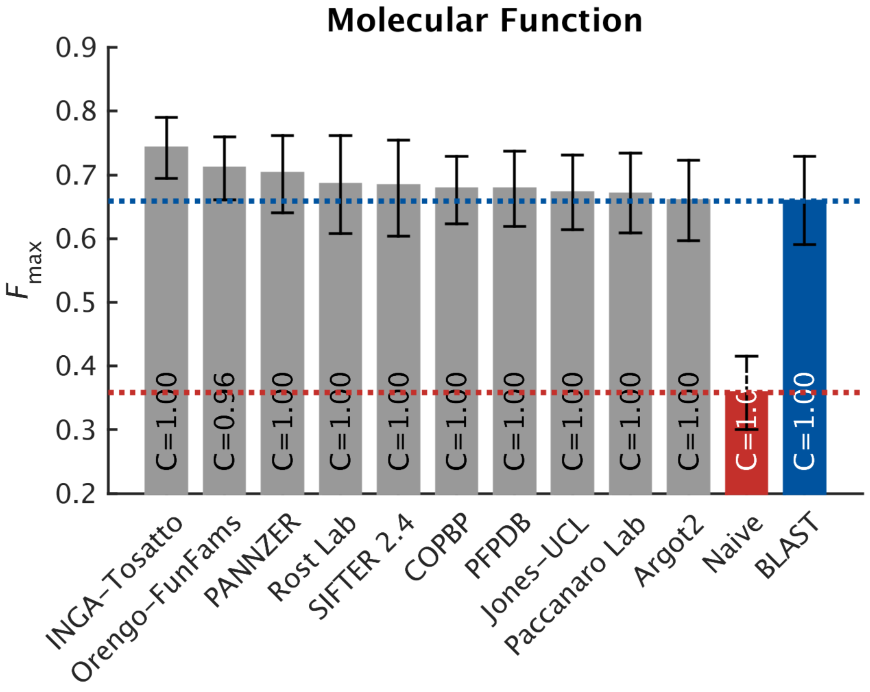

Supplementary Figure 7B (*Escherichia coli K12*):

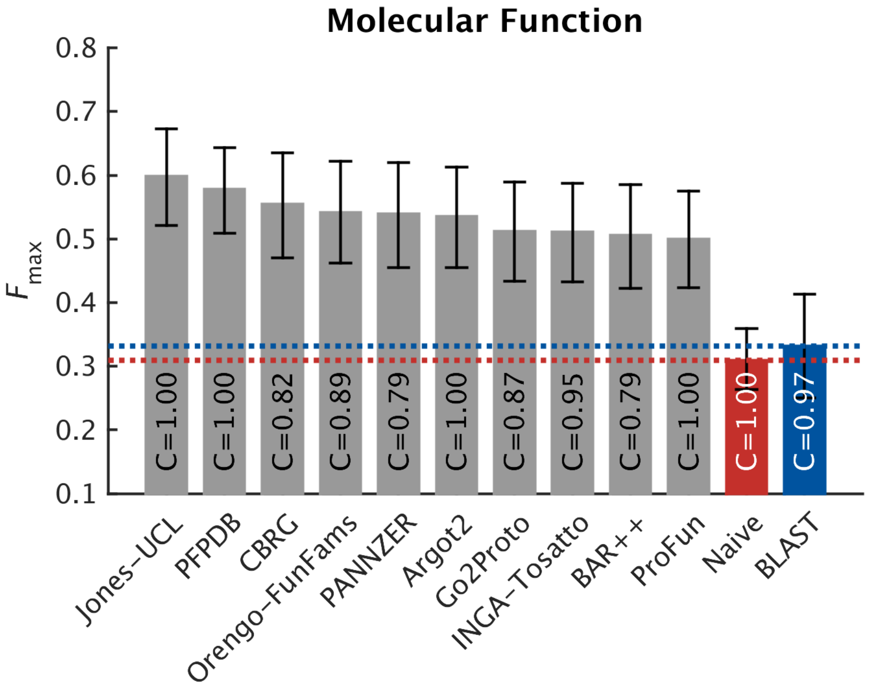

Supplementary Figure 7C (*Homo sapiens*):

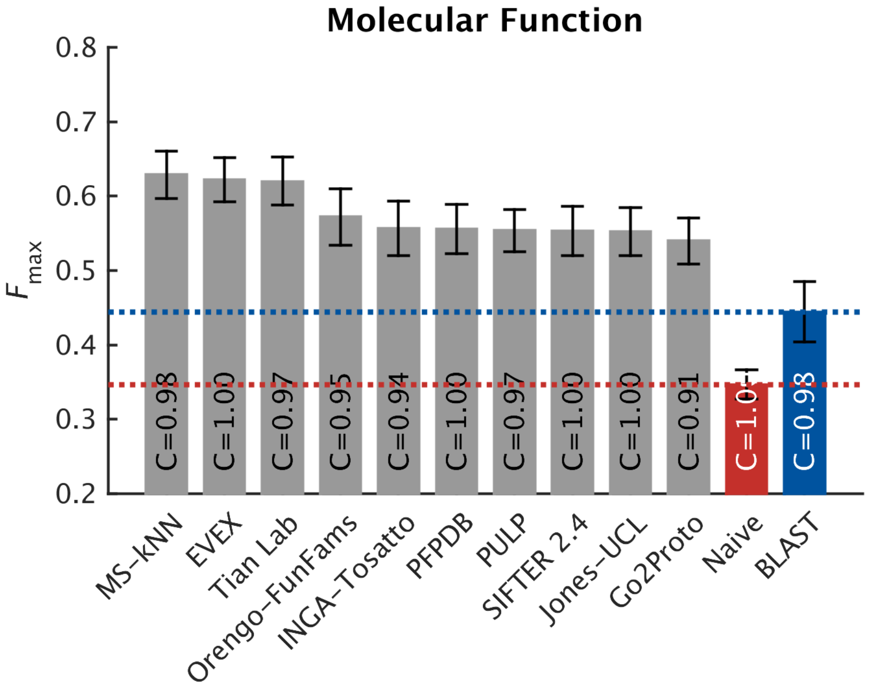

Supplementary Figure 7D (*Mus musculus*):

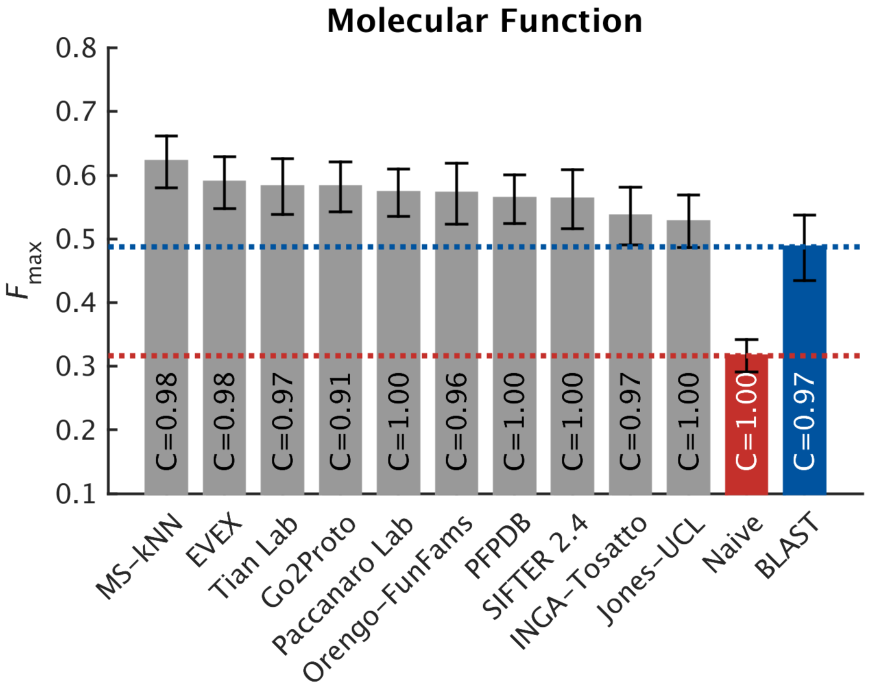

Supplementary Figure 7E (*Pseudomonas aeruginosa*):

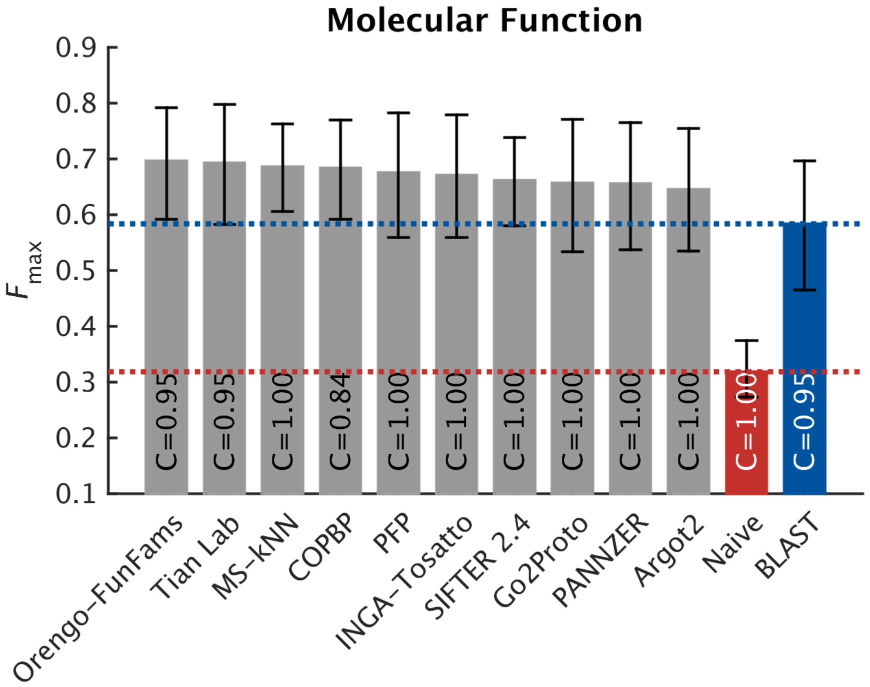

Supplementary Figure 7F (*Rattus norvegicus*):

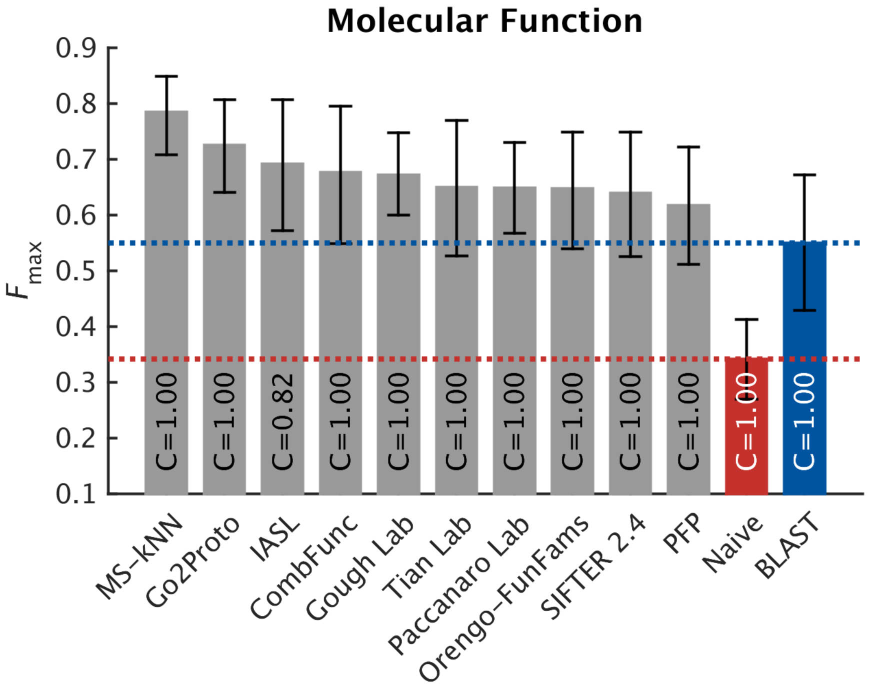

Supplementary Figure 7G (*Arabidopsis thaliana*):

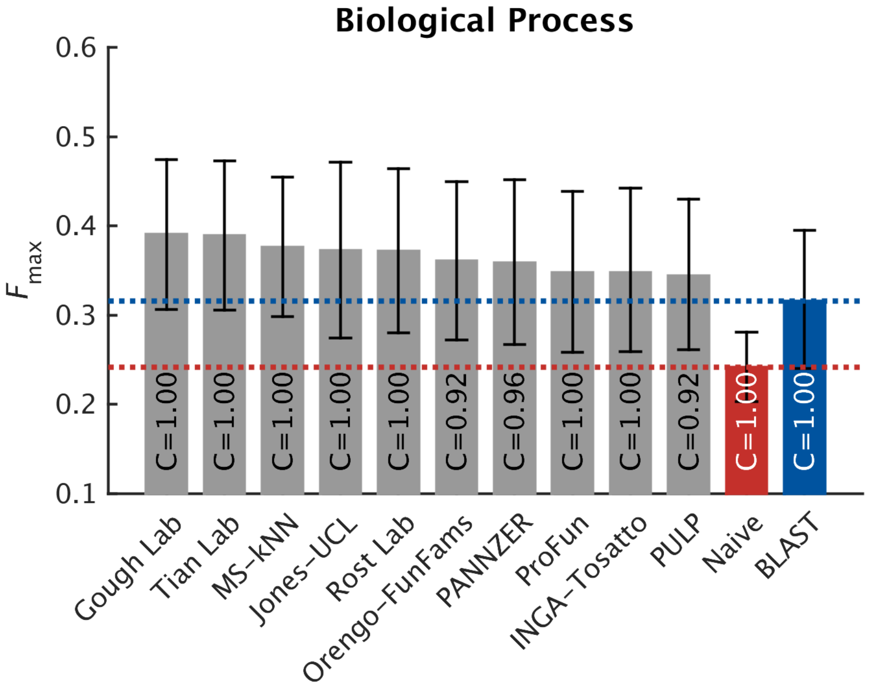

Supplementary Figure 7H (*Danio rerio*):

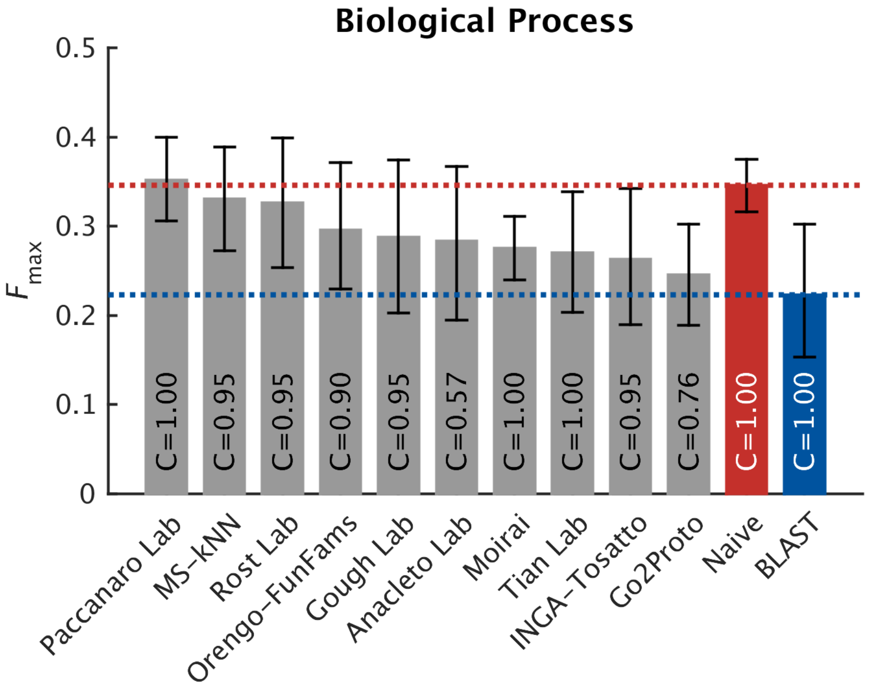

Supplementary Figure 7I (*Dictyostelium discoideum*):

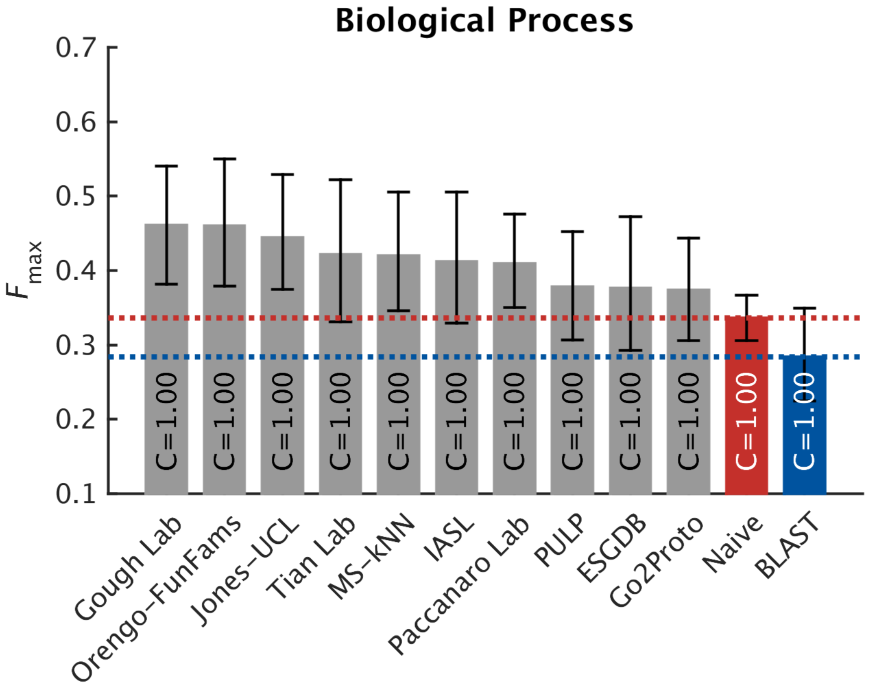

Supplementary Figure 7J (*Drosophila melanogaster*):

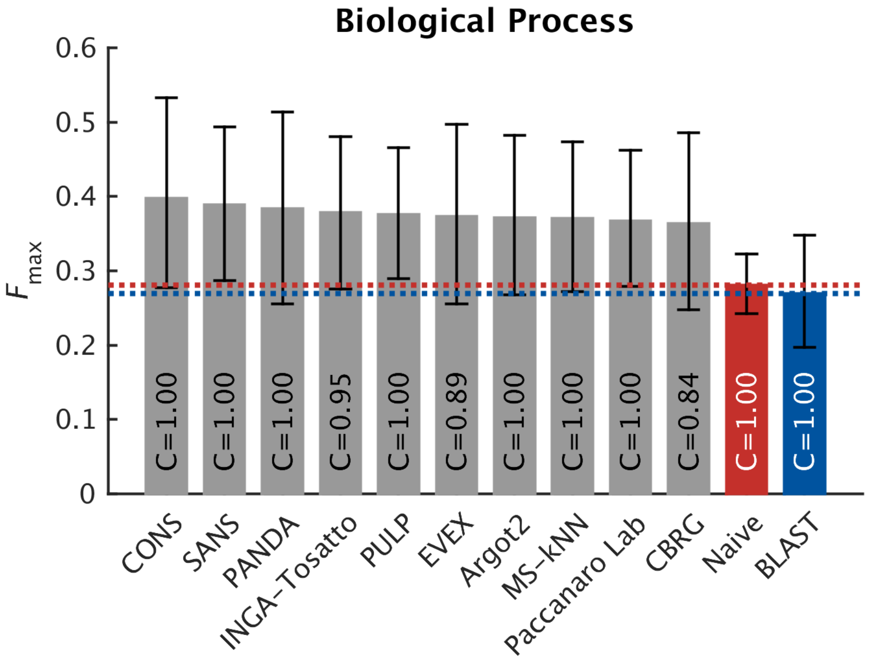

Supplementary Figure 7K (*Escherichia coli K12*):

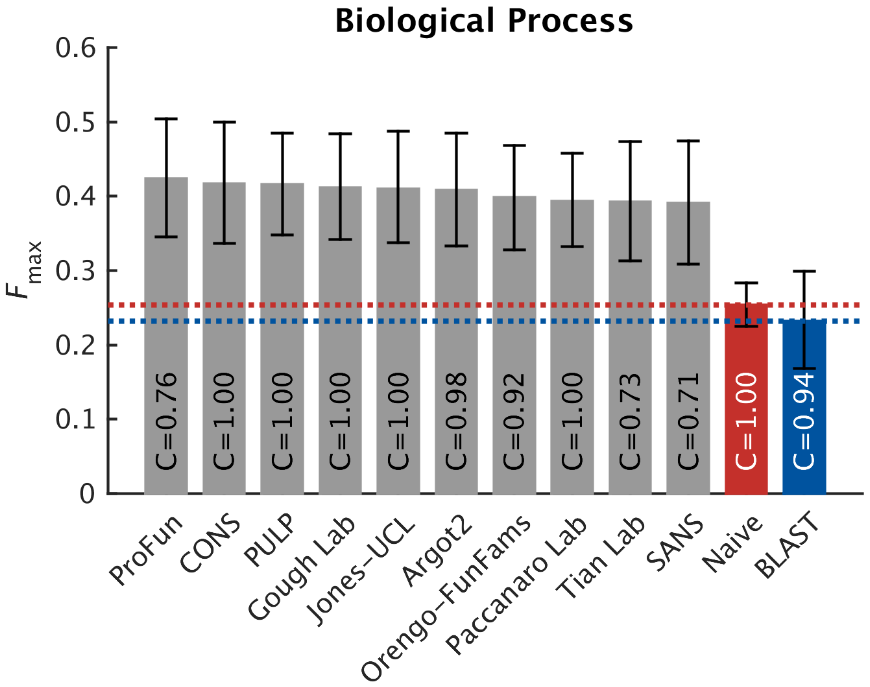

Supplementary Figure 7L (*Homo sapiens*):

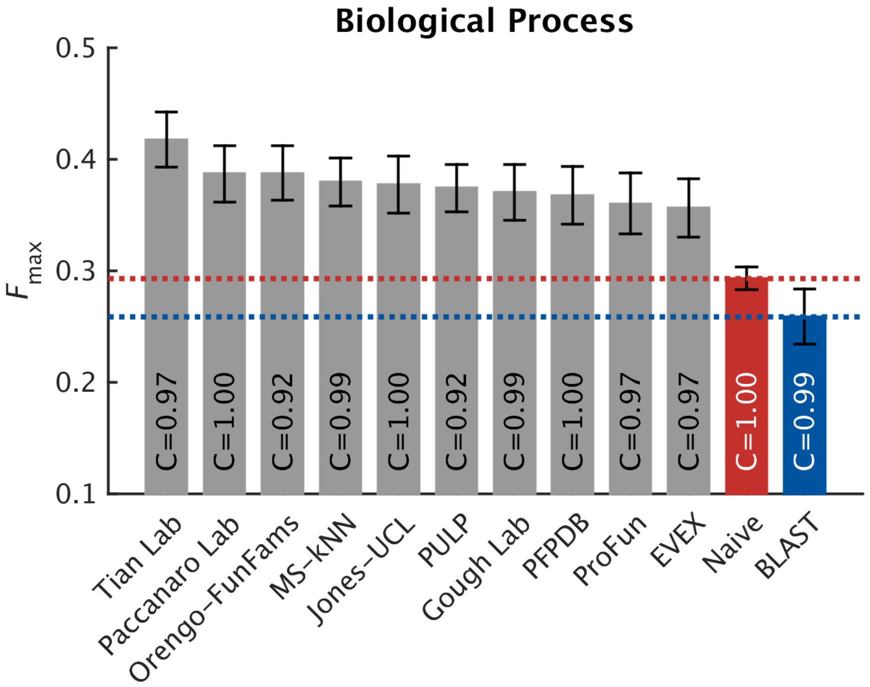

Supplementary Figure 7M (*Mus musculus*):

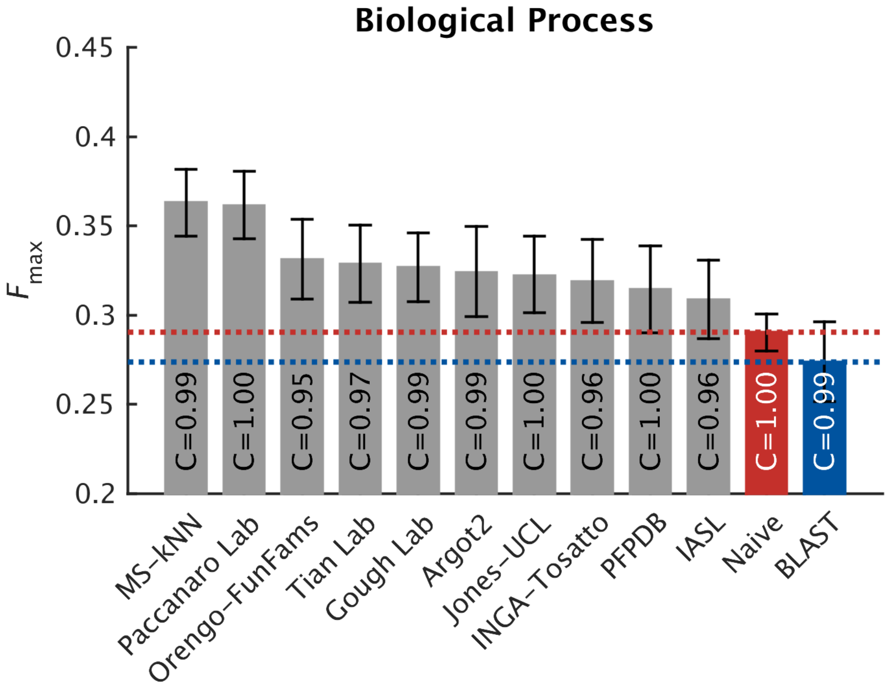

Supplementary Figure 7N (*Pseudomonas aeruginosa*):

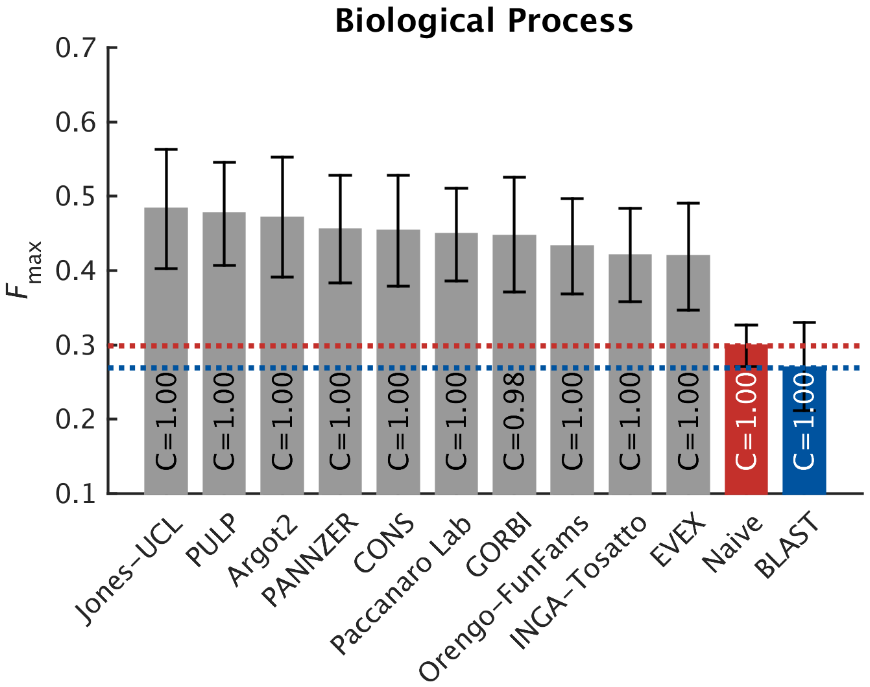

Supplementary Figure 7O (*Rattus norvegicus*):

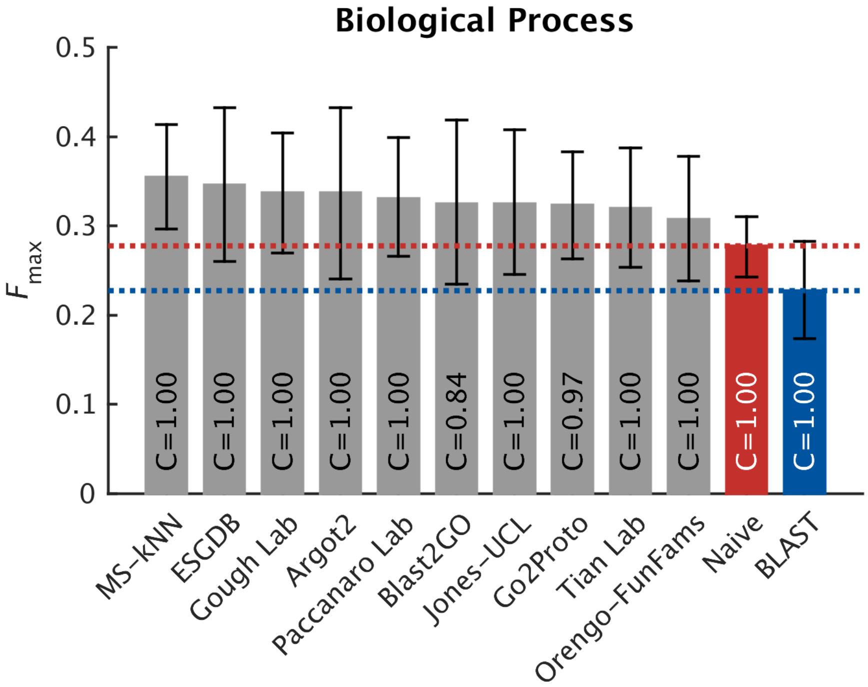

Supplementary Figure 7P (*Arabidopsis thaliana*):

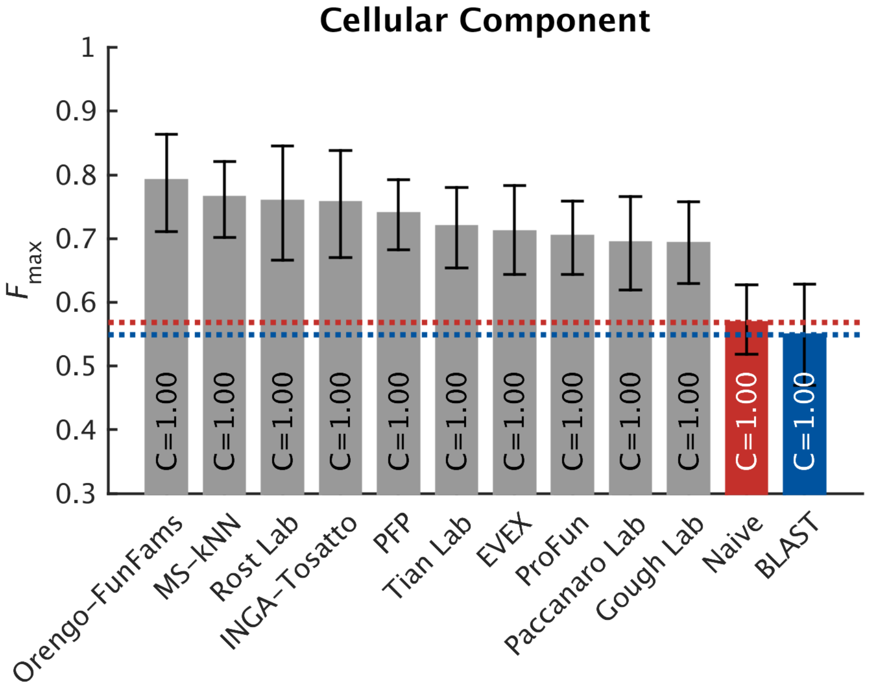

Supplementary Figure 7Q (*Drosophila melanogaster*):

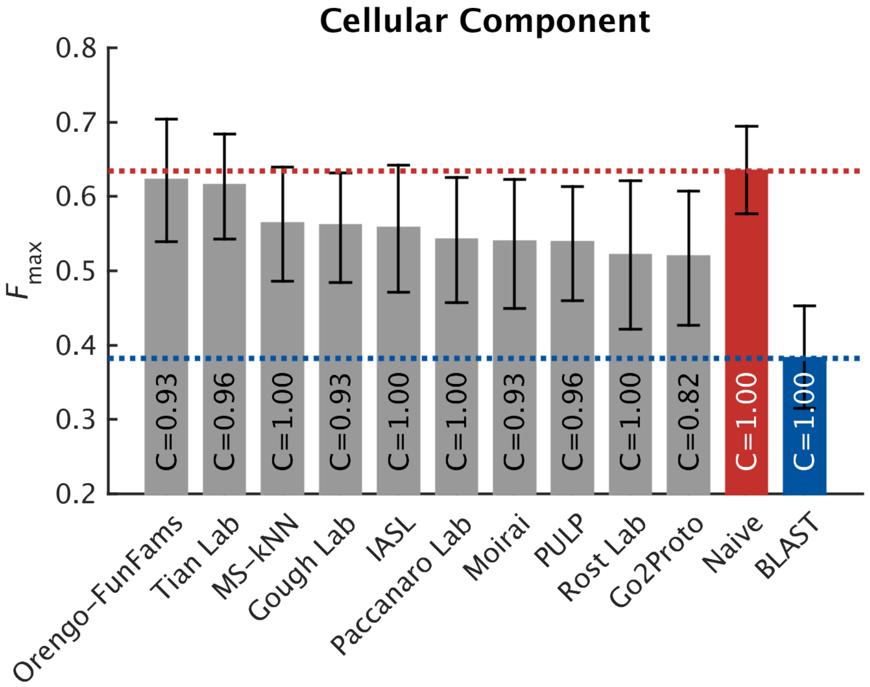

Supplementary Figure 7R (*Escherichia coli K12*):

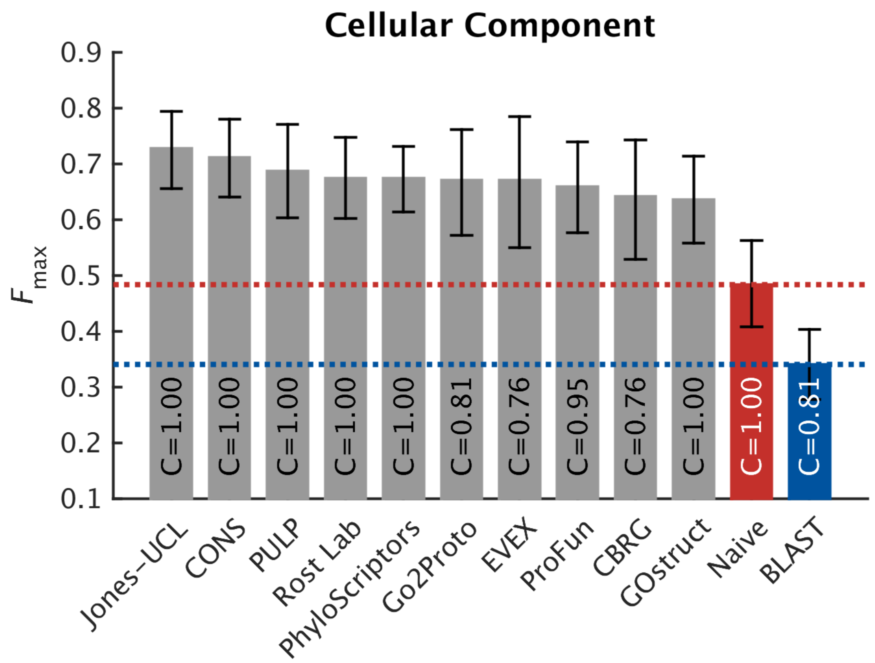

Supplementary Figure 7S (*Homo sapiens*):

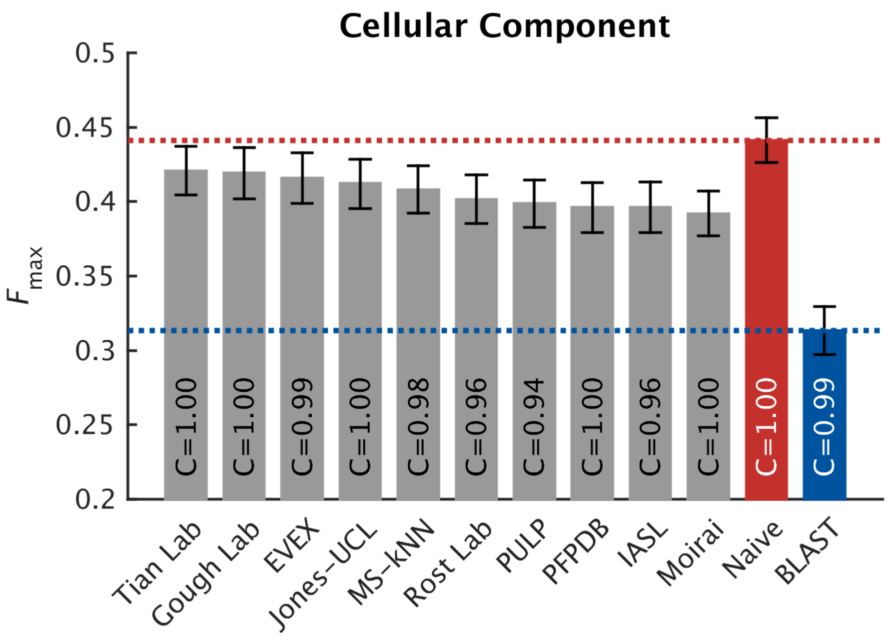

Supplementary Figure 7T (*Mus musculus*):

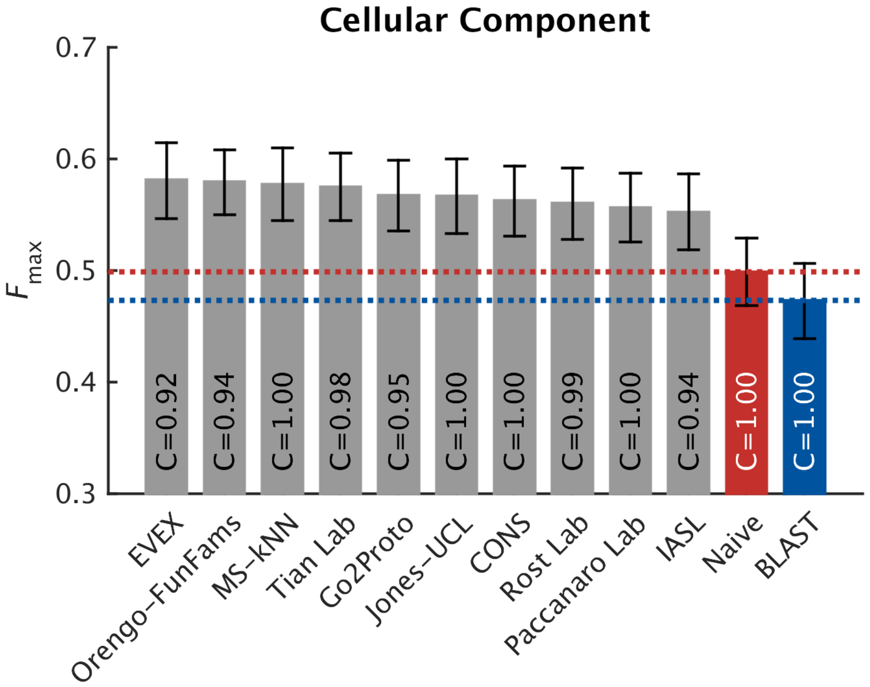

Supplementary Figure 7U (*Rattus norvegicus*):

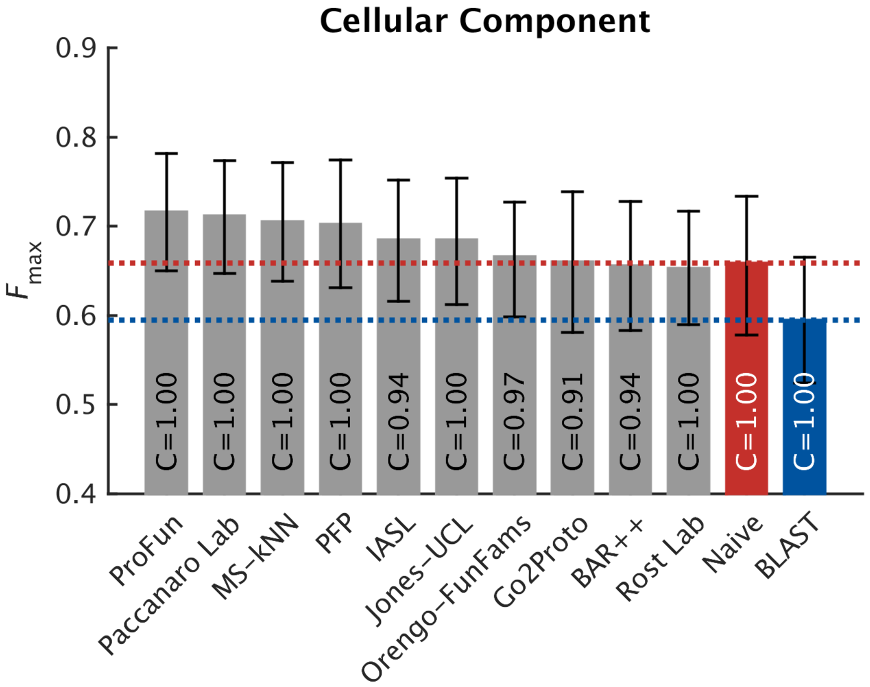

Supplementary Figure 7V (*Saccharomyces cerevisiae*):

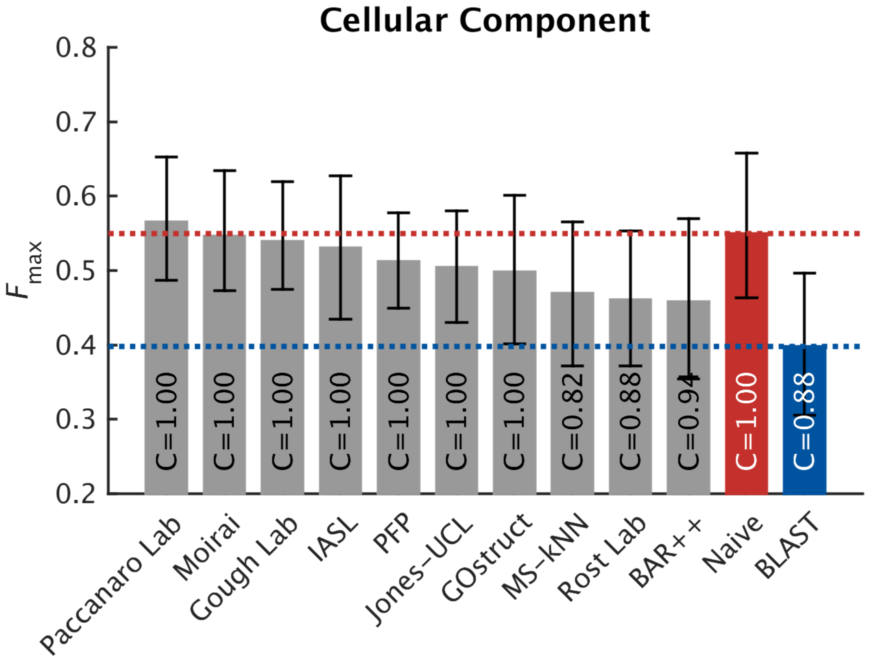

Supplementary Figure 8 Weighted precision-recall curves for the top-performing methods for (A) Molecular Function ontology, (B) Biological Process ontology, (C) Cellular Component ontology and (D) Human Phenotype ontology. All panels show the top ten participating methods in each category, as well as the Naïve and BLAST baseline methods. Points corresponding to the maximum weighted F-measure are marked in circles on each curve. The legend provides the maximum weighted F-measure (F) and coverage (C) for all methods. In cases where a Principal Investigator (PI) participated with multiple teams, only the results of the best scoring method are presented.

Calculation of the weighted precision-recall curve. Each term f in the ontology was weighted according to the information content of that term. The information content of the term f was calculated as

$$ic(f) = \log_2 \frac{1}{\Pr\left(f|\mathcal{P}(f)\right)},$$

where $\Pr\left(f|\mathcal{P}(f)\right)$ is the probability that the term f in the ontology is associated to a protein given that all of its parents are associated. (probabilities were determined based on the union of Swiss-Prot, UniProt-GOA and GO Consortium databases). Weighted precisions and recalls are calculated as

$$\begin{aligned} wpr(\tau) &= \frac{1}{m(\tau)} \sum_{i=1}^{m(\tau)} \frac{\sum_f ic(f) \cdot \mathbb{1}\left(f \in P_i(\tau) \wedge T_i(\tau)\right)}{\sum_f ic(f) \cdot \mathbb{1}\left(f \in P_i(\tau)\right)}, \quad \text{and} \\ wrc(\tau) &= \frac{1}{n_e} \sum_{i=1}^{n_e} \frac{\sum_f ic(f) \cdot \mathbb{1}\left(f \in P_i(\tau) \wedge T_i(\tau)\right)}{\sum_f ic(f) \cdot \mathbb{1}\left(f \in T_i(\tau)\right)}, \end{aligned}$$

where $P_i(\tau)$ is the set of predicted terms for protein i with score no less than threshold τ and T_i is the set of true terms for protein i, $m(\tau)$ is the number of sequences with at least one predicted score greater than or equal to τ, and n_e is the number of proteins used in a particular mode of evaluation. In the full evaluation mode $n_e = n$, the number of benchmark proteins, whereas in the partial evaluation mode $n_e = m(0)$.

Supplementary Figure 8A:

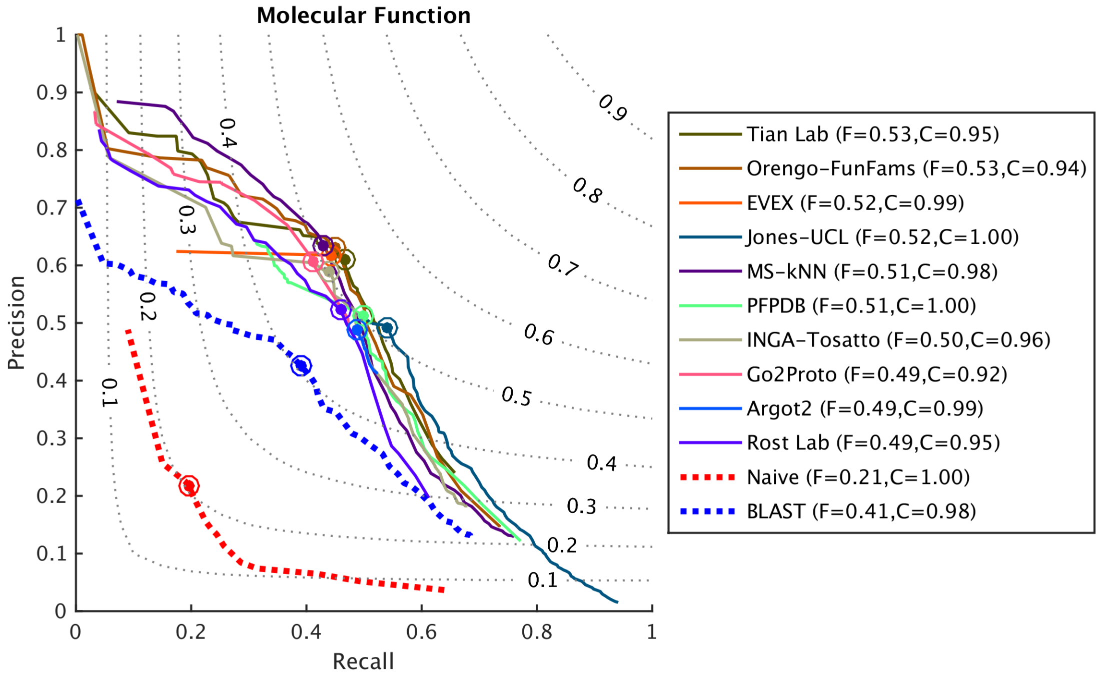

Supplementary Figure 8B:

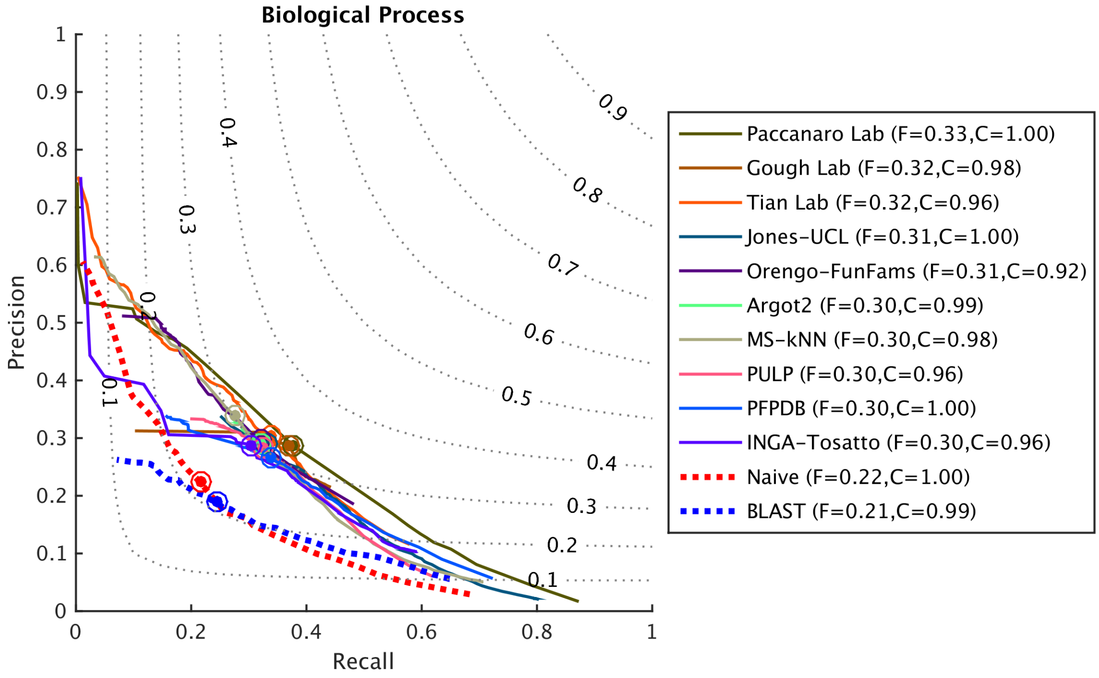

Supplementary Figure 8C:

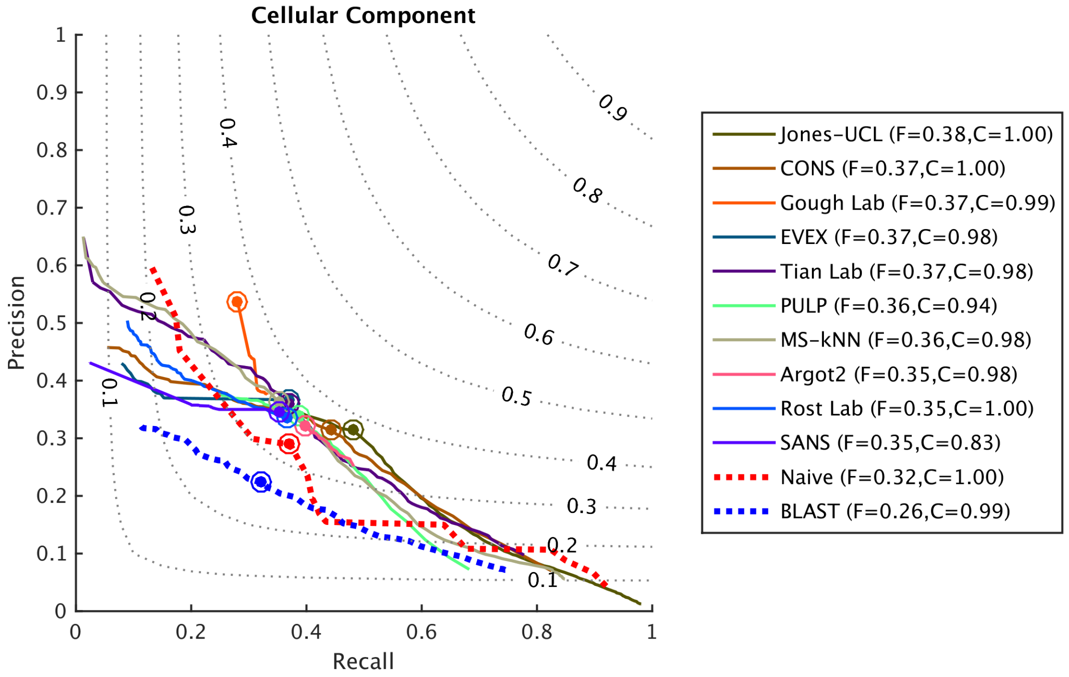

Supplementary Figure 8D:

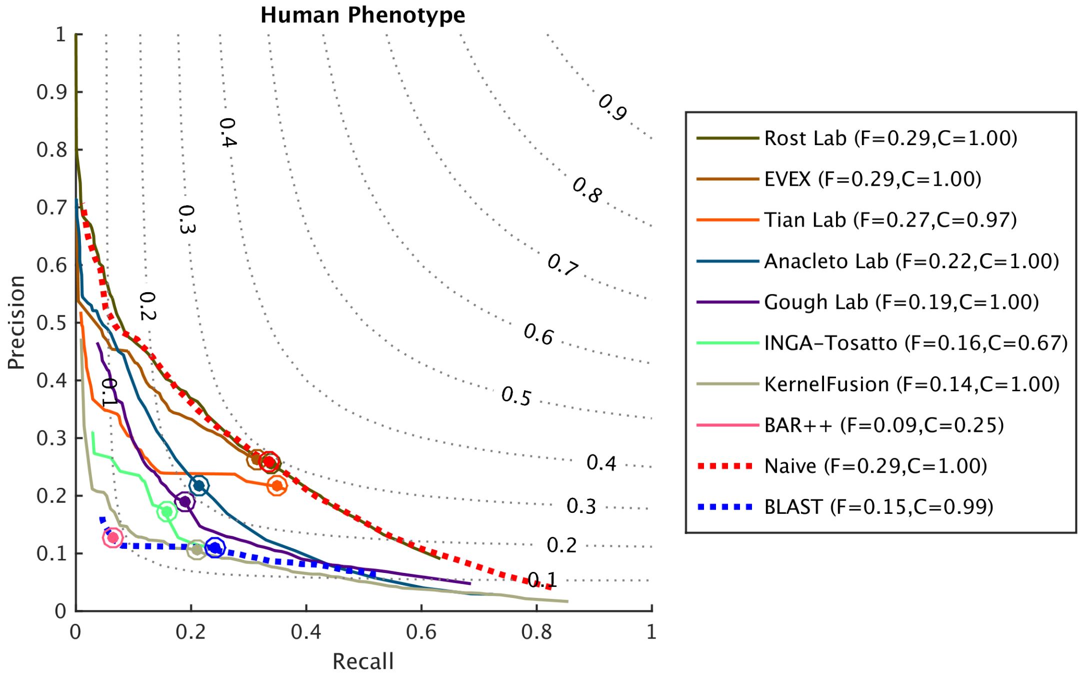

Supplementary Figure 9 Normalized remaining uncertainty-misinformation curves for the top-performing methods for (A) Molecular Function ontology, (B) Biological Process ontology, (C) Cellular Component ontology and (D) Human Phenotype ontology. All panels show the top ten participating methods in each category, as well as the Naïve and BLAST baseline methods. Points corresponding to the minimum normalized semantic distance [40] are marked in circles on each curve. The legend provides the minimum normalized semantic distance (S) and coverage (C) for all methods. In cases where a Principal Investigator (PI) participated with multiple teams, only the results of the best scoring method are presented.

Calculation of the normalized remaining uncertainty-misinformation curve.

$$
\begin{aligned}
nru(\tau) &= \frac{1}{n_e}\sum_{i=1}^{n_e}\frac{\sum_f ic(f)\cdot \mathbb{1}\left(f \notin P_i(\tau) \wedge f \in T_i\right)}{\sum_f ic(f)\cdot \mathbb{1}\left(f \in P_i(\tau) \vee f \in T_i\right)}, \quad \text{and} \\
nmi(\tau) &= \frac{1}{n_e}\sum_{i=1}^{n_e}\frac{\sum_f ic(f)\cdot \mathbb{1}\left(f \in P_i(\tau) \wedge f \notin T_i\right)}{\sum_f ic(f)\cdot \mathbb{1}\left(f \in P_i(\tau) \vee f \in T_i\right)},
\end{aligned}
$$

where $P_i(\tau)$ is the set of predicted terms for protein i with score no less than threshold τ and T_i is the set of true terms for protein i, and n_e is the number of proteins used in a particular mode of evaluation. In the full evaluation mode $n_e = n$, the number of benchmark proteins, whereas in the partial evaluation mode n_e is the number of proteins that have at least one positive predicted score.

Supplementary Figure 9A:

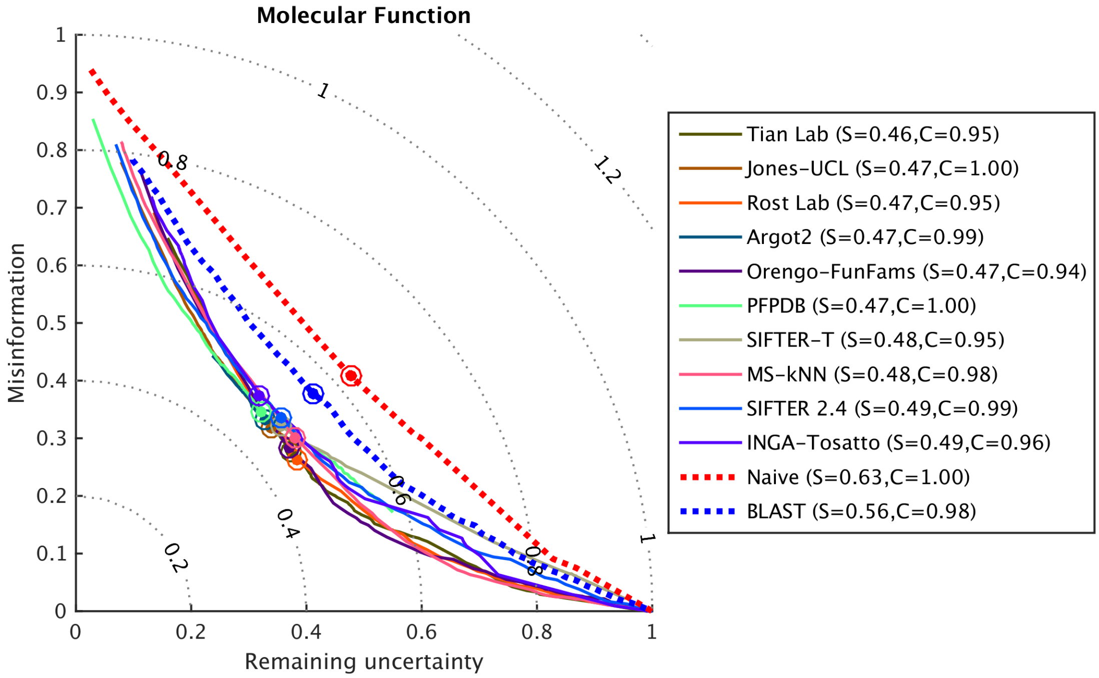

Supplementary Figure 9B:

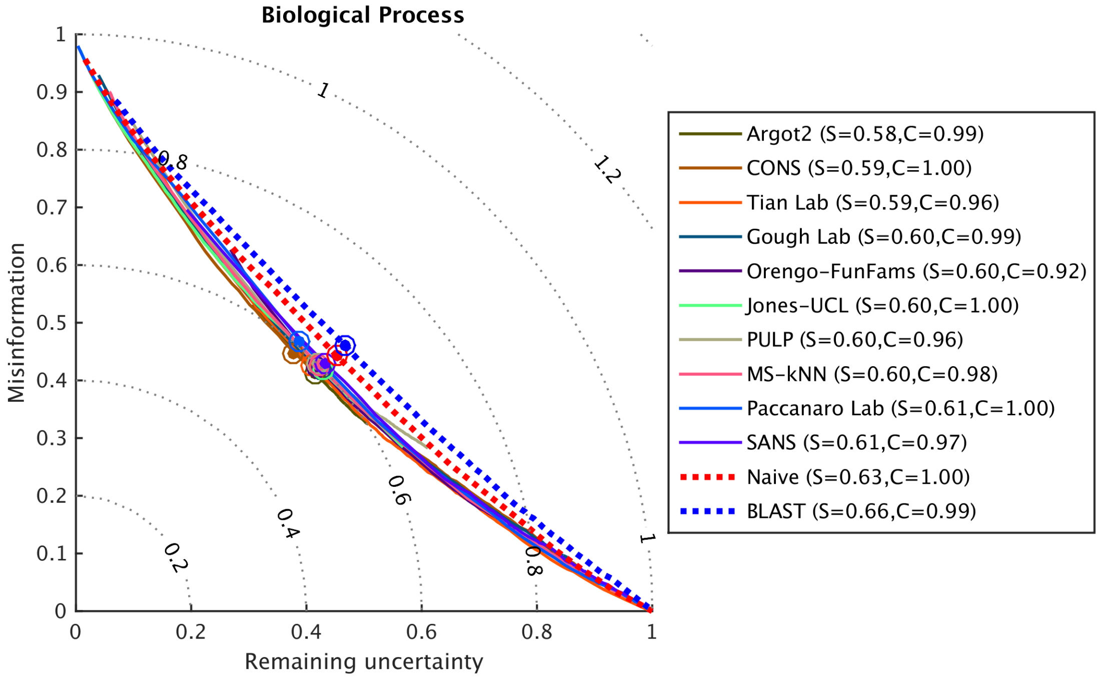

Supplementary Figure 9C:

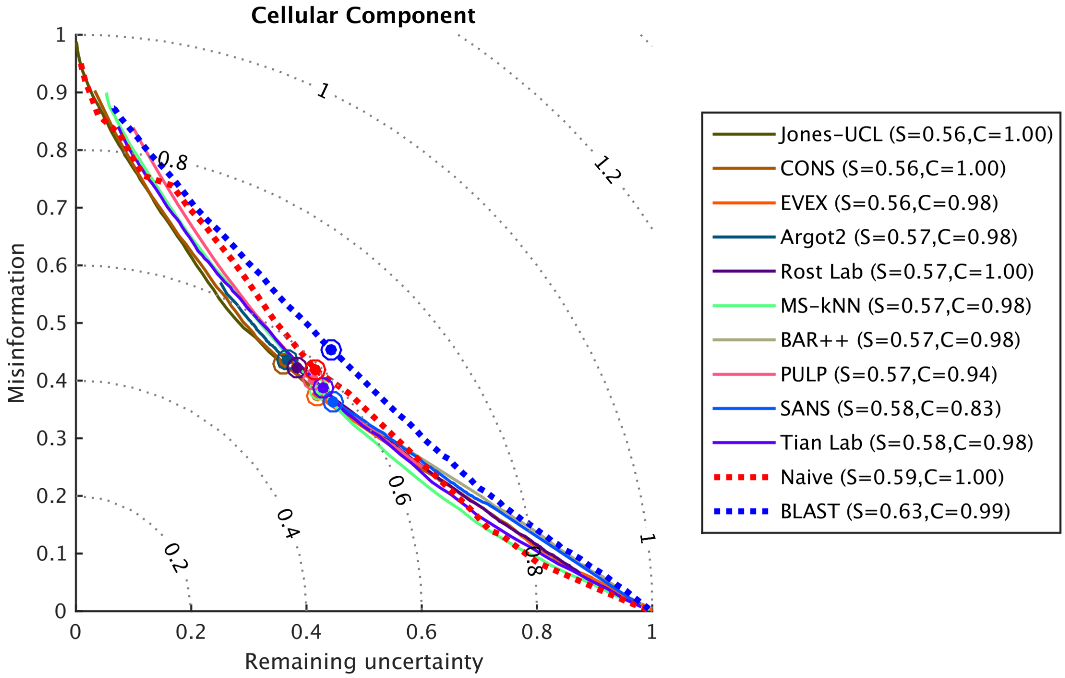

Supplementary Figure 9D:

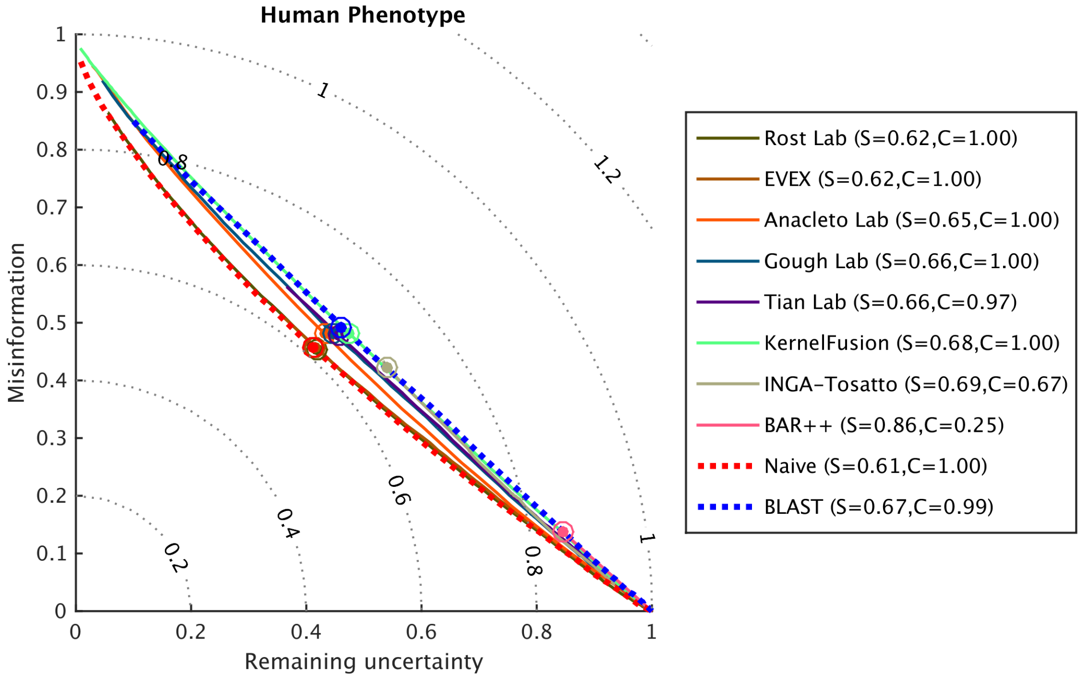

Supplementary Figure 10 Similarity network of participated methods for (A) Molecular Function ontology, (B) Biological Process ontology, (C) Cellular Component ontology and (D) Human Phenotype ontology. For all panels, similarities are computed as the Pearson's correlation coefficient between methods with a 0.75 cutoff for illustration purposes. A unique color is assigned to all methods submitted under the same principal investigator. Not evaluated (organizer's) methods are shown in triangles, while benchmark methods (Naïve and BLAST) are shown in squares. Top 10 methods are highlighted with enlarged nodes and circled in red. Edge width indicates the strength of similarity. Nodes are labelled with the name of methods followed by "team-model" if multiple teams/models are submitted.

Supplementary Figure 10A:

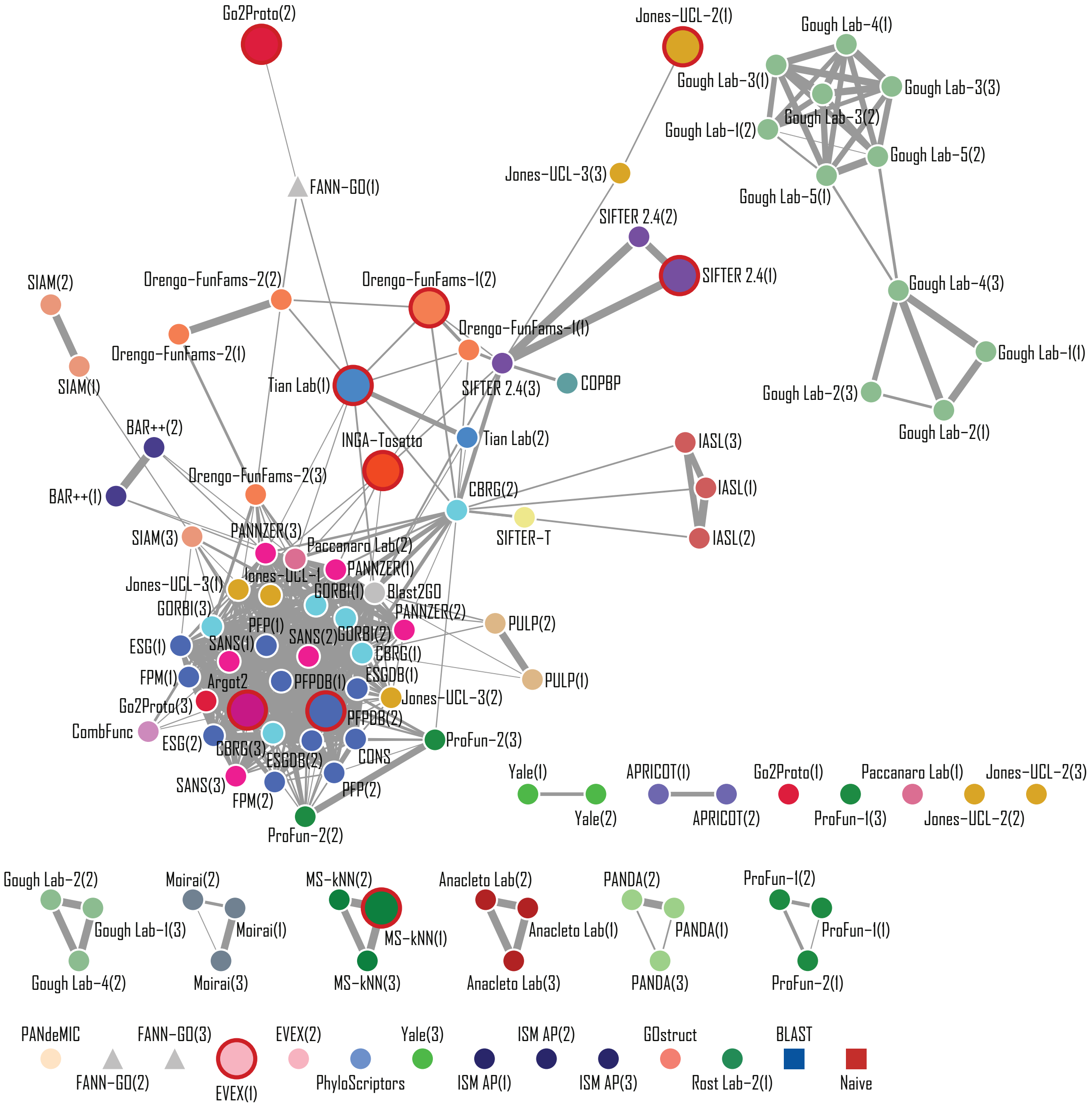

Supplementary Figure 10B:

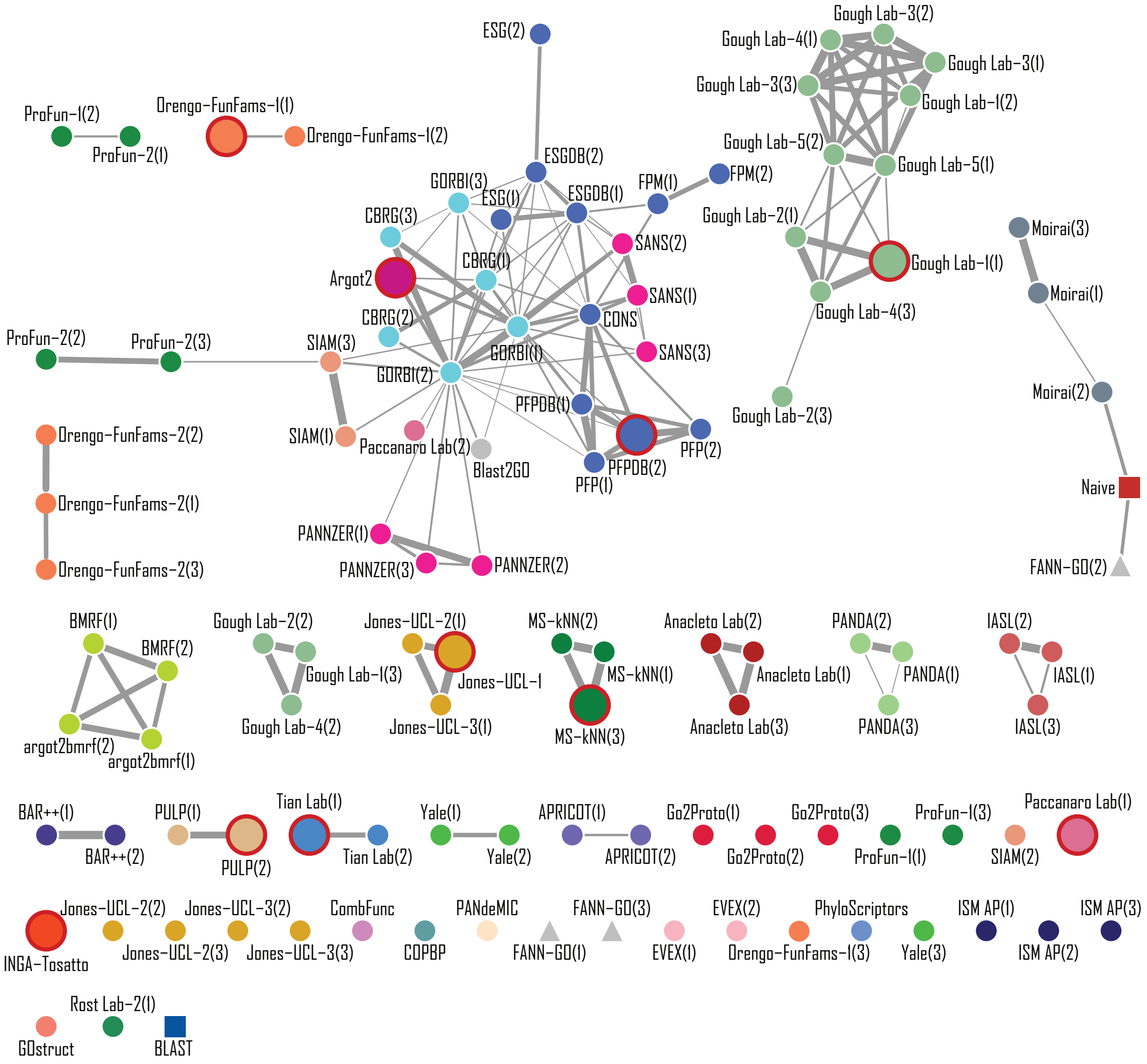

Supplementary Figure 10C:

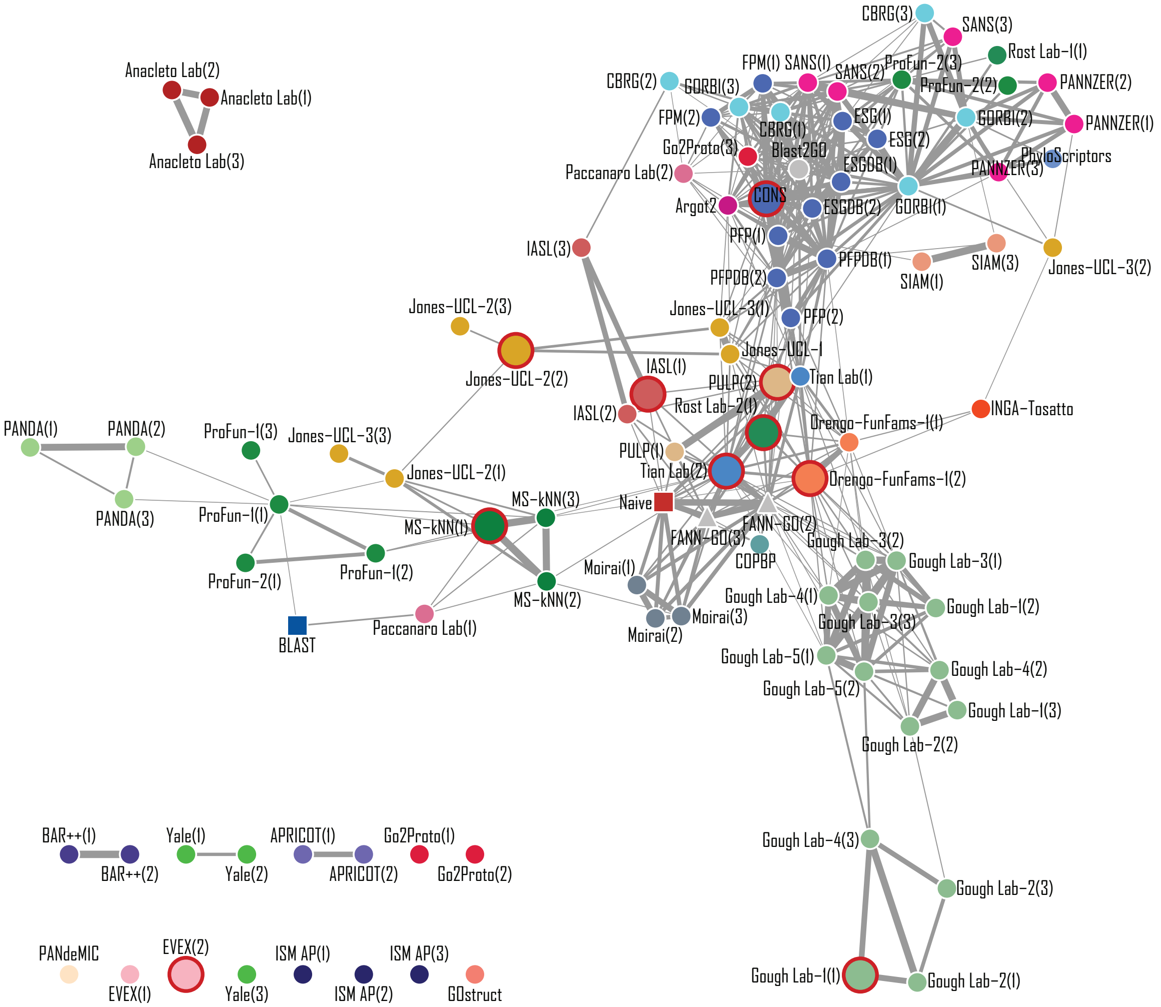

Supplementary Figure 10D:

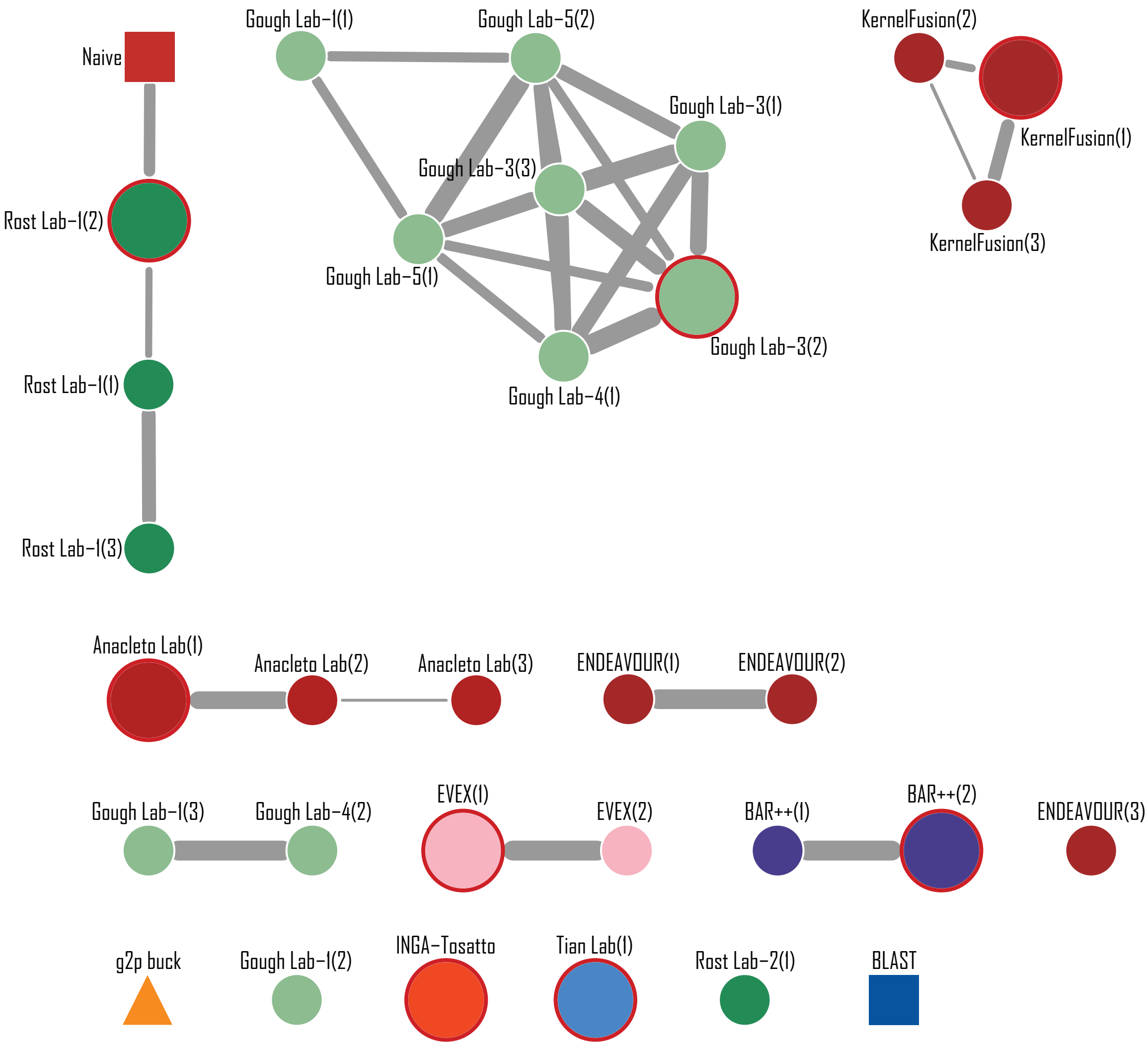

Supplementary Figure 11 The barplot of keyword frequency self-annotated by CAFA2 top 10 methods of (A) Molecular Function ontology, (B) Biological Process ontology, and (C) Cellular Component ontology. The barplot of keyword enrichment self-annotated by CAFA2 top 10 methods against all submitted methods of (D) Molecular Function ontology, (E) Biological Process ontology, and (F) Cellular Component ontology. Keyword enrichment was calculated as log-ratio of:

$$e(k) = \log \frac{\frac{1}{10}\sum_{i=1}^{10} \mathbb{1}(k \in \mathcal{K}_i)}{\frac{1}{n}\sum_{i=1}^{n} \mathbb{1}(k \in \mathcal{K}_i)},$$

where we assume methods are in descending order of their $F_{\max}$ measure and $\mathcal{K}_i$ indicates the set of self-annotated keywords by model i.

Supplementary Figure 11A:

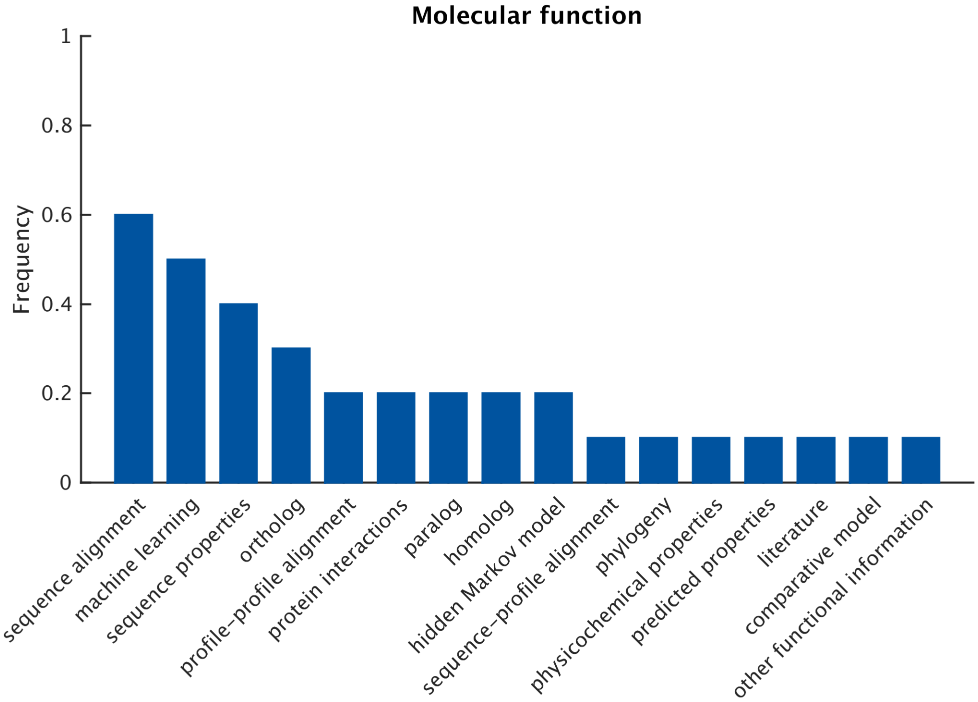

Supplementary Figure 11B:

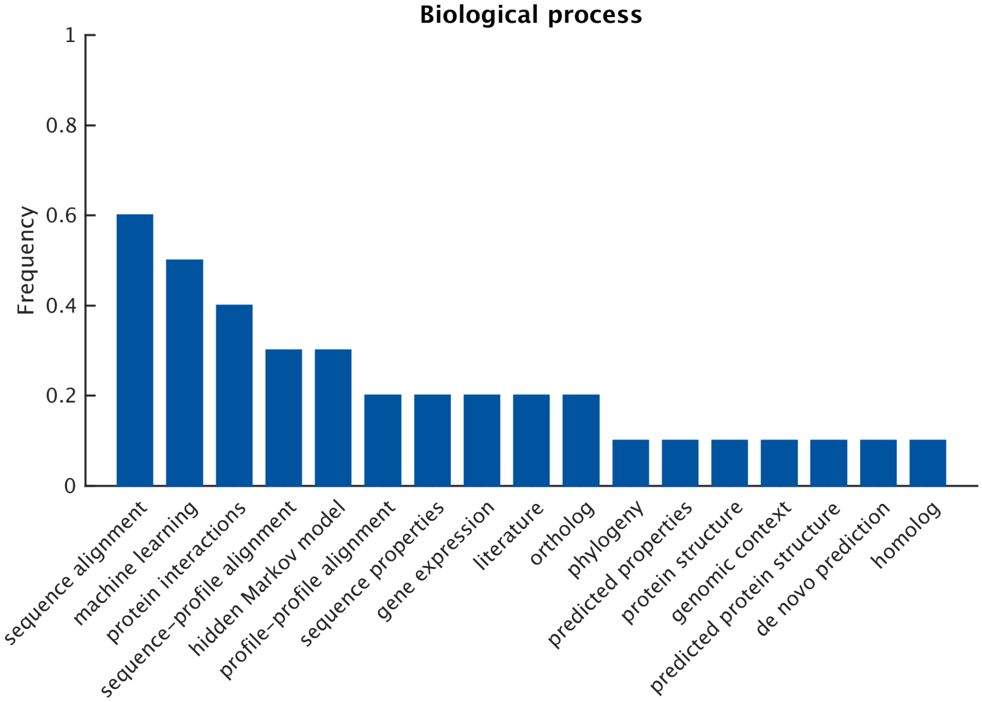

Supplementary Figure 11C:

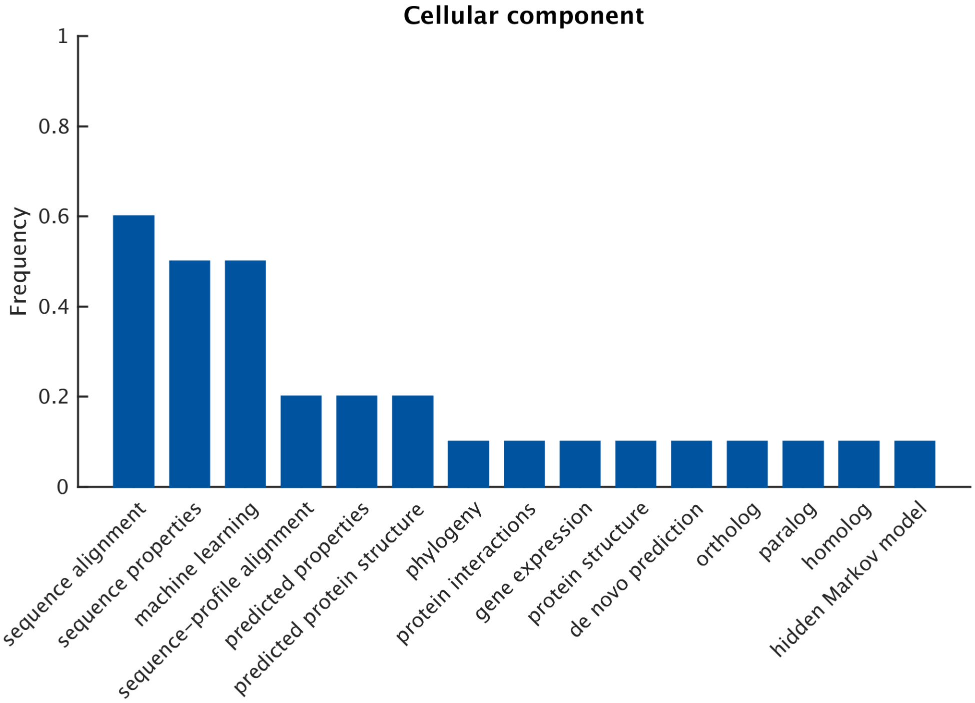

Supplementary Figure 11D:

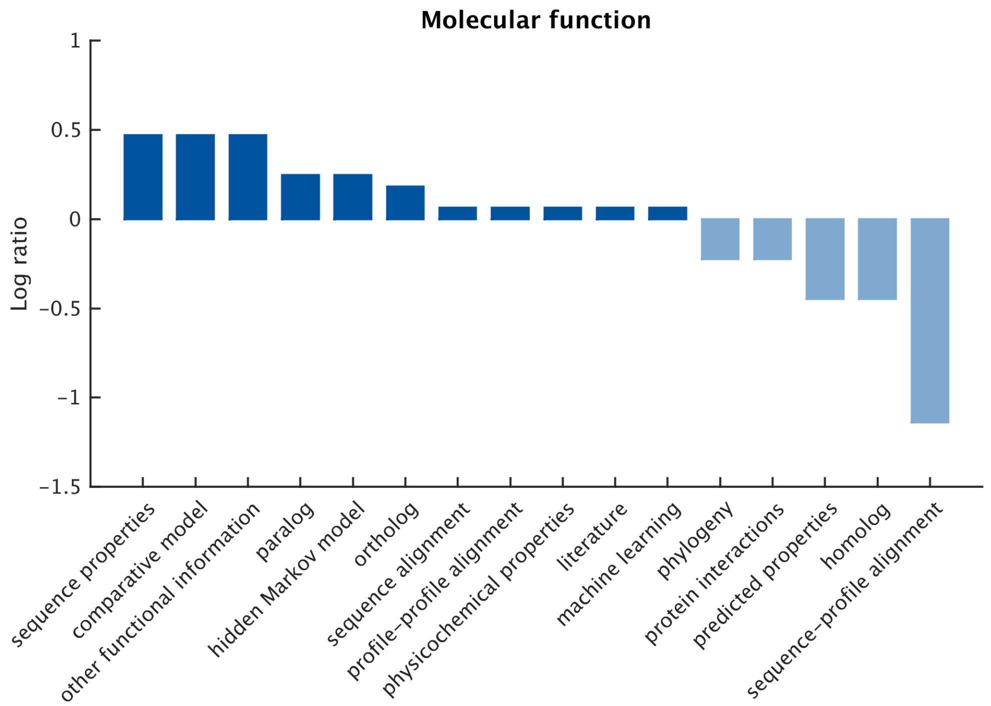

Supplementary Figure 11E:

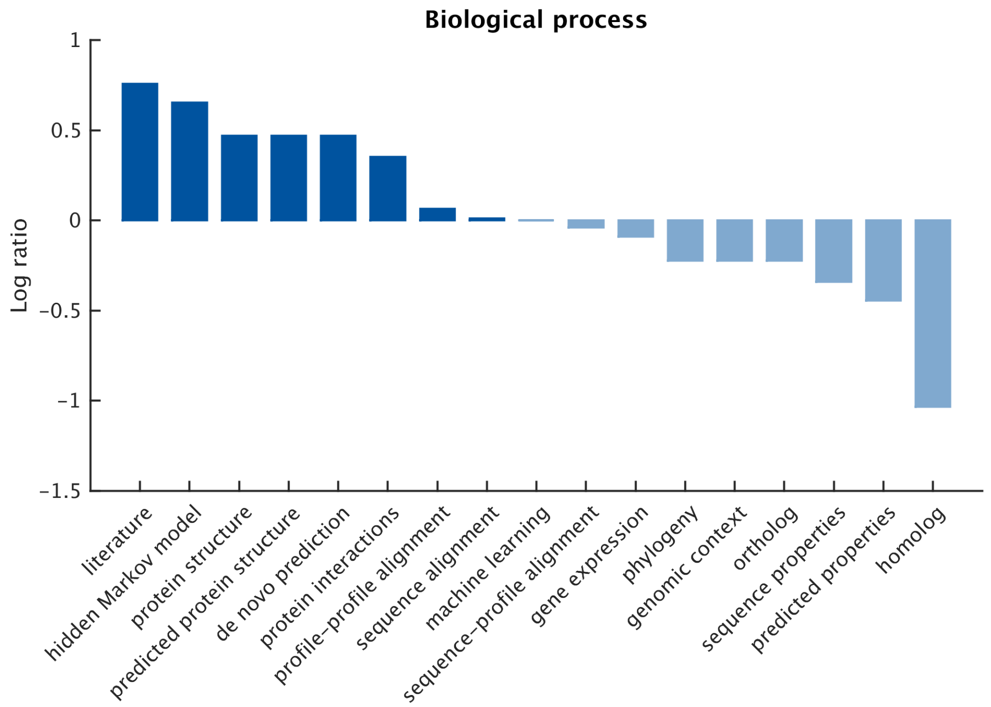

Supplementary Figure 11F:

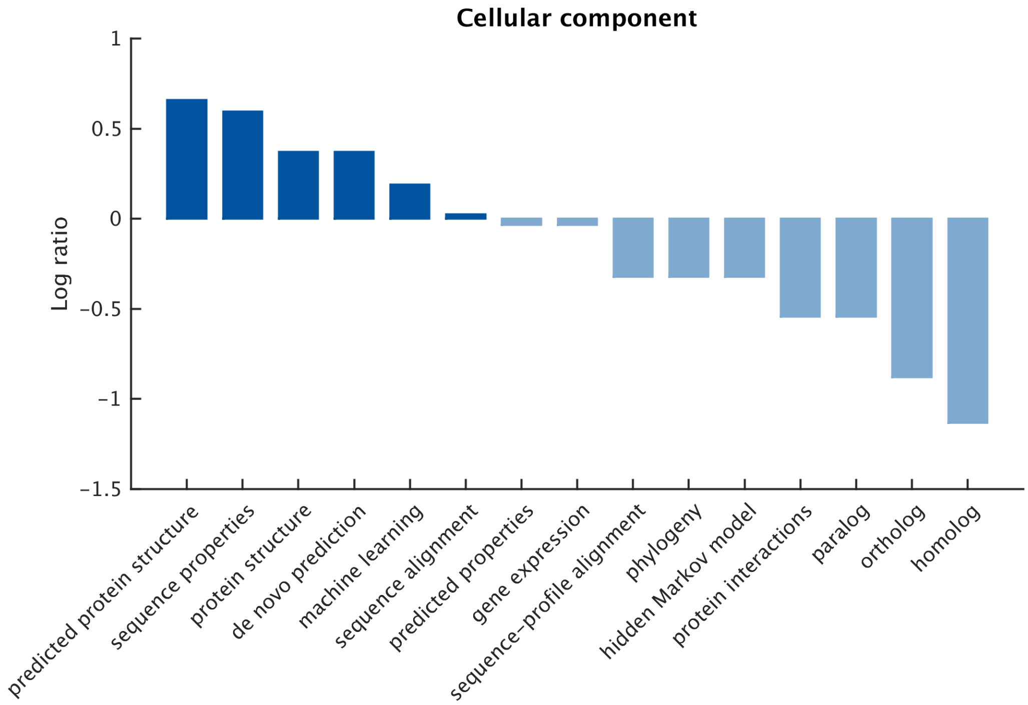

Supplementary Table 1. (Part 1) Participating methods grouped according to Principal Investigators (PIs)

Principal Investigator	Method Name	Model (keyword)	Publications
Asa Ben-Hur	GOstruct	Model 1 (sa,sp,pp,pi,ge,gi,lt,gc,ml,nlp)	[36]
Richard Bonneau	PULP	Model 1 (ph,sp,pp,pi,ge,ps,pps,dp,ml,or)	[43, 42, 41]
		Model 2 (ph,sp,pp,pi,ge,ps,pps,dp,ml)	
Steven Brenner	SIFTER 2.4 †	Model 1 (ph,ml,or,pa,ho)	[34, 15]
		Model 2 (ph,ml,or,pa,ho)	
		Model 3 (ph,ml,or,pa,ho)	
Rita Casadio	BAR++	Model 1 (sa,spa,pp,pps,ml,ho,hmm)	[4, 32]
		Model 2 (sa,spa,pp,pps,ml,ho,hmm)	
Jianlin Cheng	ProFun	Model 1 (spa,sp,gi,gc,dp,gd)	[6]
		Model 2 (spa,dp)	
		Model 3 (spa,gi,gc,dp,gd)	
	ProFun/donet	Model 1 (ppa,spa)	[38]
		Model 2 (ppa,spa)	
		Model 3 (ppa,spa)	
Wyatt Clark	Yale	Model 1 (pi)	
		Model 2 (pi)	
		Model 3 (pi)	
Christophe Dessimoz	GORBI	Model 1 (ml,or,pa,ho,gc)	[35]
		Model 2 (ml,or,pa,ho,gc)	
		Model 3 (or,pa,ho,sa,spa,ppa,ph,hmm)	
	CBRG	Model 1 (or,pa,ho)	[3]
		Model 2 (or,pa,ho)	
		Model 3 (or)	
Tunca Dogan	PANdeMIC	Model 1 (sa,ml,ho)	
Filip Ginter	EVEX	Model 1 (sa,ml,sp)	[37]
		Model 2 (sa,ml,sp)	
Julian Gough	Gough Lab/GoughGroup	Model 1 (sa,spa,hmm)	
		Model 2 (pps,hmm)	
		Model 3 (pi)	
	Gough Lab/D2P2	Model 1 (pp,sa,spa,hmm)	[30]
		Model 2 (pp,pi)	
		Model 3 (pp)	
	Gough Lab/dcGO	Model 1 (pps,pp,sa,spa,hmm,pi)	[17]
		Model 2 (pps,pp,sa,spa,hmm,pi)	
		Model 3 (pps,pp,sa,spa,hmm,pi)	
	Gough Lab/SUPERFAMILY	Model 1 (pps,pp,sa,spa,hmm,pi)	[14]
		Model 2 (pi)	
		Model 3 (pp,sa,spa,hmm)	
	Gough Lab/dcGOpredictor	Model 1 (pps,sa,spa,hmm,pi)	
		Model 2 (pps,sa,spa,hmm,pi)	
Liisa Holm	SANS	Model 1 (sa)	[24]
		Model 2 (sa)	
		Model 3 (sa)	
	PANNZER	Model 1 (sa,ph,or,pa,ho,nlp,ofi)	[25]
		Model 2 (sa,ph,or,pa,ho,nlp,ofi)	
		Model 3 (sa,ph,or,pa,ho,nlp,ofi)	
Wen-Lian Hsu	IASL	Model 1 (sa,spa,sp)	
		Model 2 (sa,spa,sp)	
		Model 3 (sa,spa,sp)	
David Jones	Jones-UCL/jfpred-RF	Model 1 (hmm,ppa,sp,pi,or,lt,ml)	[11]
	Jones-UCL/jfpred-FP	Model 1 (hmm,ppa,sp,pi,or,lt,ml)	
		Model 2 (sp,pp,pps,ml)	
		Model 3 (sp,pp,pps,ml)	
	Jones-UCL/jfpred-PB	Model 1 (hmm,ppa,sp,pi,or,lt,ml)	
		Model 2 (sa,spa)	
		Model 3 (hmm,ppa)	

†SIFTER is expected to work well on microbial proteins.

Supplementary Table 1. (Part 2)

Principal Investigator	Method Name	Model (keyword)	Publications
Daisuke Kihara	ESG	Model 1 (sa)<br>Model 2 (sa)	[7]
	CONS	Model 1 (sa)	[23]
	FPM	Model 1 (sa)<br>Model 2 (sa)	
	PFPDB	Model 1 (sa)<br>Model 2 (sa)	
	ESGDB	Model 1 (sa)<br>Model 2 (sa)	
	PFP	Model 1 (sa)<br>Model 2 (sa)	[22, 21]
Sean Mooney	g2p buck (not evaluated)	Model 1 (N/A)	
Michal Linial	Go2Proto	Model 1 (sa,sp,php,pp,cm,ml,or,pa,ho,ofi)<br>Model 2 (sa,sp,php,pp,cm,ml,or,pa,ho,ofi)<br>Model 3 (sa,sp,php,pp,cm,ml,or,pa,ho,ofi)	
Yves Moreau	ENDEAVOUR	Model 1 (sa,ph,pi,ge,lt,ml,ofi)<br>Model 2 (sa,ph,pi,ge,lt,ml,ofi)<br>Model 3 (sa,ph,pi,ge,lt,ml,ofi)	[1]
	KernelFusion	Model 1 (sa,pi,ge,lt,ml,ofi)<br>Model 2 (sa,pi,ge,lt,ml,ofi)<br>Model 3 (sa,pi,ge,lt,ml,ofi)	[44, 13]
Christine Orengo	Orengo-FunFams/MDA	Model 1 (ml)<br>Model 2 (sp)<br>Model 3 (pi)	[12]
	Orengo-FunFams	Model 1 (spa,ppa,ho,hmm)<br>Model 2 (spa,ppa,ho,hmm)<br>Model 3 (spa,ppa,ho,hmm)	
Alberto Paccanaro	Paccanaro Lab	Model 1 (sa,spa,pi,ge,lt,gc,ml,or.ho)<br>Model 2 (spa,hmm,ml)	
Paul Pavlidis	Moirai	Model 1 (ofi)<br>Model 2 (ofi)<br>Model 3 (ofi)	
Predrag Radivojac	FANN-GO (not evaluated)	Model 1 (sa,ml)<br>Model 2 (sa,ml)<br>Model 3 (sa,ml)	[8]
Burkhard Rost	Rost Lab	Model 1 (sa,spa,ppa,sp,dp,ml)<br>Model 2 (sa,spa,ppa,sp,dp,ml)<br>Model 3 (sa,spa,ppa,sp,dp,ml)	[18]
	Rost Lab/metastudent2	Model 1 (sa,ml,or,pa,ho)	[20]
Asaf Salamov	COPBP	Model 1 (N/A)	
Fran Supek	PhyloScriptors	Model 1 (ph,gc,ml,pa,or)	
Weidong Tian	Tian Lab	Model 1 (sa)<br>Model 2 (sa)	[19]
Stefano Toppo	Argot2	Model 1 (sa,spa)	[16]
Toppo/van Dijk *	argot2bmrf	Model 1 (sp,pi,ge,gi,ml,sa,spa)<br>Model 2 (sp,pi,ge,gi,ml,sa,spa)	
Silvio Tosatto	INGA-Tosatto	Model 1 (hmm,ppa,sa,pi)	[31]
Michael Tress	SIAM	Model 1 (sa,ho,sp,ps,php,spa,ppa,sta,cm)<br>Model 2 (ps,php,spa,ppa,sta,cm)<br>Model 3 (sa,ho,sp)	[29]
Hafeez Ur Rehman	PFPPipeLine	Model 1 (sa,pi,ml,ho,ofi)	[5]
Giorgio Valentini	Anacleto Lab	Model 1 (ml,sa)<br>Model 2 (ml,sa)<br>Model 3 (ml,sa)	[33]
Aalt-Jan van Dijk	BMRF	Model 1 (sp,pi,ge,gi,ml)<br>Model 2 (sp,pi,ge,gi,ml)	[26, 27]
Nevena Veljkovic	ISM AP	Model 1 (ppa,php)<br>Model 2 (ppa,php,ge)<br>Model 3 (ppa,php,ge)	

* This is a joint group of Stefano Toppo and Aalt-Jan van Dijk.

Supplementary Table 1. (Part 3)

Principal Investigator	Method Name	Model (keyword)	Publications
Ricardo Vencio	SIFTER-T	Model 1 (spa,ml,ho)	[2]
Jörg Vogel	APRICOT	Model 1 (ho,hmm,ppa,pp)<br>Model 2 (ho,hmm,ppa,pp)	
Slobodan Vucetic	MS-kNN	Model 1 (ml,sa,ge)<br>Model 2 (ml,sa,ge)<br>Model 3 (ml,sa,ge)	[28]
Zheng Wang	PANDA	Model 1 (spa,ppa,ph,or,pa,ho)<br>Model 2 (spa,ppa,ph,or,pa,ho)<br>Model 3 (spa,ppa,ph,or,pa,ho)	
Mark Wass	CombFunc	Model 1 (spa,sa,ml,ge,pi)	[39]
N/A ‡	Blast2GO	Model 1 (sa)	[10]

‡Blast2GO predictions were downloaded from the website https://www.blast2go.com one week before the prediction deadline and converted into appropriate submission format by the CAFA organizers.

Supplementary Table 1. (Part 4) Keyword table.

Code	Keyword	Code	Keyword
sa	sequence alignment	sta	structure alignment
spa	sequence-profile alignment	cm	comparative model
ppa	profile-profile alignment	pps	predicted protein structure
ph	phylogeny	dp	*de novo* prediction
sp	sequence properties	ml	machine learning
php	physicochemical properties	gne	genome environment
pp	predicted properties	op	operon
pi	protein interactions	or	ortholog
ge	gene expression	pa	paralog
ms	mass spectrometry	ho	homolog
gi	genetic interactions	hmm	hidden Markov model
ps	protein structure	cd	clinical data
lt	literature	gd	genetic data
gc	genomic context	nlp	natural language processing
sy	synteny	ofi	other functional information

References

[1] S. Aerts, D. Lambrechts, S. Maity, P. Van Loo, B. Coessens, F. De Smet, L. C. Tranchevent, B. De Moor, P. Marynen, B. Hassan, P. Carmeliet, and Y. Moreau. Gene prioritization through genomic data fusion. *Nat Biotechnol*, 24(5):537–544, 2006.

[2] D. C. Almeida-e Silva and R. Z. Vencio. SIFTER-T: a scalable and optimized framework for the SIFTER phylogenomic method of probabilistic protein domain annotation. *Biotechniques*, 58(3):140–142, 2015.

[3] A. M. Altenhoff, N. Skunca, N. Glover, C. M. Train, A. Sueki, I. Pilizota, K. Gori, B. Tomiczek, S. Muller, H. Redestig, G. H. Gonnet, and C. Dessimoz. The OMA orthology database in 2015: function predictions, better plant support, synteny view and other improvements. *Nucleic Acids Res*, 43(Database issue):D240–249, 2015.

[4] L. Bartoli, L. Montanucci, R. Fronza, P. L. Martelli, P. Fariselli, L. Carota, G. Donvito, G. P. Maggi, and R. Casadio. The Bologna Annotation Resource: a non hierarchical method for the functional and structural annotation of protein sequences relying on a comparative large-scale genome analysis. *J Proteome Res*, 8(9):4362–4371, 2009.

[5] A. Benso, S. Di Carlo, H. Ur Rehman, G. Politano, A. Savino, and P. Suravajhala. A combined approach for genome wide protein function annotation/prediction. *Proteome Sci*, 11(Suppl 1):S1, 2013.

[6] R. Cao and J. Cheng. Integrated protein function prediction by mining function associations, sequences, and protein-protein and gene-gene interaction networks. *Methods*, 2015.

[7] M. Chitale, T. Hawkins, C. Park, and D. Kihara. ESG: extended similarity group method for automated protein function prediction. *Bioinformatics*, 25(14):1739–1745, 2009.

[8] W. T. Clark and P. Radivojac. Analysis of protein function and its prediction from amino acid sequence. *Proteins*, 79(7):2086–2096, 2011.

[9] W. T. Clark and P. Radivojac. Information-theoretic evaluation of predicted ontological annotations. *Bioinformatics*, 29(13):i53–i61, 2013.

[10] A. Conesa, S. Gotz, J. M. Garcia-Gomez, J. Terol, M. Talon, and M. Robles. Blast2GO: a universal tool for annotation, visualization and analysis in functional genomics research. *Bioinformatics*, 21(18):3674–3676, 2005.

[11] D. Cozzetto, D. W. Buchan, K. Bryson, and D. T. Jones. Protein function prediction by massive integration of evolutionary analyses and multiple data sources. *BMC Bioinformatics*, 14 Suppl 3:S1, 2013.

[12] S. Das, D. Lee, I. Sillitoe, N. L. Dawson, J. G. Lees, and C. A. Orengo. Functional classification of CATH superfamilies: a domain-based approach for protein function annotation. *Bioinformatics*, 2015.

[13] T. De Bie, L. C. Tranchevent, L. M. van Oeffelen, and Y. Moreau. Kernel-based data fusion for gene prioritization. *Bioinformatics*, 23(13):i125–132, 2007.

[14] D. A. de Lima Morais, H. Fang, O. J. Rackham, D. Wilson, R. Pethica, C. Chothia, and J. Gough. Superfamily 1.75 including a domain-centric gene ontology method. *Nucleic Acids Res*, 39(Database issue):D427–434, 2011.

[15] B. E. Engelhardt, M. I. Jordan, J. R. Srouji, and S. E. Brenner. Genome-scale phylogenetic function annotation of large and diverse protein families. *Genome Res*, 21(11):1969–1980, 2011.

[16] M. Falda, S. Toppo, A. Pescarolo, E. Lavezzo, B. Di Camillo, A. Facchinetti, E. Cilia, R. Velasco, and P. Fontana. Argot2: a large scale function prediction tool relying on semantic similarity of weighted gene ontology terms. *BMC Bioinformatics*, 13(Suppl 4):S14, 2012.

[17] H. Fang and J. Gough. A domain-centric solution to functional genomics via dcGO predictor. *BMC Bioinformatics*, 14 Suppl 3:S9, 2013.

[18] T. Goldberg, M. Hecht, T. Hamp, T. Karl, G. Yachdav, N. Ahmed, U. Altermann, P. Angerer, S. Ansorge, K. Balasz, M. Bernhofer, A. Betz, L. Cizmadija, K. T. Do, J. Gerke, R. Greil, V. Joerdens, M. Hastreiter, K. Hembach, M. Herzog, M. Kalemanov, M. Kluge, A. Meier, H. Nasir, U. Neumaier, V. Prade, J. Reeb, A. Sorokoumov, I. Troshani, S. Vorberg, S. Waldraff, J. Zierer, H. Nielsen, and B. Rost. LocTree3 prediction of localization. *Nucleic Acids Res*, 42(Web Server issue):W350–355, 2014.

[19] Q. Gong, W. Ning, and W. Tian. GoFDR: a sequence alignment based method for predicting protein functions. *Methods*, 2015.

[20] T. Hamp, R. Kassner, S. Seemayer, E. Vicedo, C. Schaefer, D. Achten, F. Auer, A. Boehm, T. Braun, M. Hecht, M. Heron, P. Honigschmid, T. A. Hopf, S. Kaufmann, M. Kiening, D. Krompass, C. Landerer, Y. Mahlich, M. Roos, and B. Rost. Homology-based inference sets the bar high for protein function prediction. *BMC Bioinformatics*, 14 Suppl 3:S7, 2013.

[21] T. Hawkins, M. Chitale, S. Luban, and D. Kihara. PFP: Automated prediction of gene ontology functional annotations with confidence scores using protein sequence data. *Proteins*, 74(3):566–582, 2009.

[22] T. Hawkins, S. Luban, and D. Kihara. Enhanced automated function prediction using distantly related sequences and contextual association by PFP. *Protein Sci*, 15(6):1550–1556, 2006.

[23] I. K. Khan, Q. Wei, S. Chapman, D. B. Kc, and D. Kihara. The PFP and ESG protein function prediction methods in 2014: effect of database updates and ensemble approaches. *Gigascience*, 4:43, 2015.

[24] J. P. Koskinen and L. Holm. SANS: high-throughput retrieval of protein sequences allowing 50% mismatches. *Bioinformatics*, 28(18):i438–i443, 2012.

[25] P. Koskinen, P. Toronen, J. Nokso-Koivisto, and L. Holm. PANNZER: high-throughput functional annotation of uncharacterized proteins in an error-prone environment. *Bioinformatics*, 31(10):1544–1552, 2015.

[26] Y. A. Kourmpetis, A. D. van Dijk, M. C. Bink, R. C. van Ham, and C. J. ter Braak. Bayesian Markov Random Field analysis for protein function prediction based on network data. *PLoS One*, 5(2):e9293, 2010.

[27] Y. A. Kourmpetis, A. D. van Dijk, R. C. van Ham, and C. J. ter Braak. Genome-wide computational function prediction of arabidopsis proteins by integration of multiple data sources. *Plant Physiol*, 155(1):271–281, 2011.

[28] L. Lan, N. Djuric, Y. Guo, and S. Vucetic. MS-kNN: protein function prediction by integrating multiple data sources. *BMC Bioinformatics*, 14 Suppl 3:S8, 2013.

[29] P. Maietta, G. Lopez, A. Carro, B. J. Pingilley, L. G. Leon, A. Valencia, and M. L. Tress. FireDB: a compendium of biological and pharmacologically relevant ligands. *Nucleic Acids Res*, 42(Database issue):D267–272, 2014.

[30] M. E. Oates, P. Romero, T. Ishida, M. Ghalwash, M. J. Mizianty, B. Xue, Z. Dosztanyi, V. N. Uversky, Z. Obradovic, L. Kurgan, A. K. Dunker, and J. Gough. D(2)P(2): database of disordered protein predictions. *Nucleic Acids Res*, 41(Database issue):D508–516, 2013.

[31] D. Piovesan, M. Giollo, E. Leonardi, C. Ferrari, and S. C. Tosatto. INGA: protein function prediction combining interaction networks, domain assignments and sequence similarity. *Nucleic Acids Res*, 43(W1):W134–140, 2015.

[32] D. Piovesan, P. L. Martelli, P. Fariselli, A. Zauli, I. Rossi, and R. Casadio. BAR-PLUS: the Bologna Annotation Resource Plus for functional and structural annotation of protein sequences. *Nucleic Acids Res*, 39(Web Server issue):W197–202, 2011.

[33] M. Re, M. Mesiti, and G. Valentini. A fast ranking algorithm for predicting gene functions in biomolecular networks. *IEEE/ACM Trans Comput Biol Bioinform*, 9(6):1812–1818, 2012.

[34] S. M. Sahraeian, K. R. Luo, and S. E. Brenner. SIFTER search: a web server for accurate phylogeny-based protein function prediction. *Nucleic Acids Res*, 43(W1):W141–147, 2015.

[35] N. Skunca, M. Bosnjak, A. Krisko, P. Panov, S. Dzeroski, T. Smuc, and F. Supek. Phyletic profiling with cliques of orthologs is enhanced by signatures of paralogy relationships. *PLoS Comput Biol*, 9(1):e1002852, 2013.

[36] A. Sokolov and A. Ben-Hur. Hierarchical classification of gene ontology terms using the gostruct method. *J Bioinform Comput Biol*, 8(2):357–376, 2010.

[37] S. Van Landeghem, K. Hakala, S. Ronnqvist, T. Salakoski, Y. Van de Peer, and F. Ginter. Exploring biomolecular literature with EVEX: connecting genes through events, homology, and indirect associations. *Adv Bioinformatics*, 2012:582765, 2012.

[38] Z. Wang, R. Cao, and J. Cheng. Three-level prediction of protein function by combining profile-sequence search, profile-profile search, and domain co-occurrence networks. *BMC Bioinformatics*, 14 Suppl 3:S3, 2013.

[39] M. N. Wass, G. Barton, and M. J. Sternberg. CombFunc: predicting protein function using heterogeneous data sources. *Nucleic Acids Res*, 40(Web Server issue):W466–470, 2012.

[40] R. Yang, Y. Jiang, M. W. Hahn, E. A. Housworth, and P. Radivojac. New metrics for learning and inference on sets, ontologies, and functions. *arXiv preprint arXiv:1603.06846*, 2016.

[41] N. Youngs. *Positive-unlabeled learning in the context of protein function prediction.* Ph.d. thesis, New York University, 2014.

[42] N. Youngs, D. Penfold-Brown, R. Bonneau, and D. Shasha. Negative example selection for protein function prediction: the NoGO database. *PLoS Comput Biol*, 10(6):e1003644, 2014.

[43] N. Youngs, D. Penfold-Brown, K. Drew, D. Shasha, and R. Bonneau. Parametric Bayesian priors and better choice of negative examples improve protein function prediction. *Bioinformatics*, 29(9):1190–1198, 2013.

[44] P. Zakeri, B. Moshiri, and M. Sadeghi. Prediction of protein submitochondria locations based on data fusion of various features of sequences. *J Theor Biol*, 269(1):208–216, 2011.
